# Humans experienced abrupt environmental changes at White Sands during the Last Glacial Maximum

**DOI:** 10.1126/sciadv.aeh0707

**Published:** 2026-10-14

**Authors:** Kathleen B. Springer, Jeffrey S. Pigati, Jessica E. Tierney, Jeffrey S. Honke, Gonzalo Jiménez-Moreno, David Bustos, Thomas M. Urban, Daniel Odess, Edward A. Jolie, Alison J. Smith, Abigail J. Hunt, Patrick J. Martinez, Vincent L. Santucci, Sally C. Reynolds, Matthew R. Bennett

**Affiliations:** ^1^ US Geological Survey, Denver Federal Center, Box 25046, MS 980, Denver, CO 80225, USA.; ^2^ Department of Geosciences, The University of Arizona, 1040 E 4th St, Tucson, AZ 85721, USA.; ^3^ Departamento de Estratigrafía y Paleontología, Universidad de Granada, Granada, Spain.; ^4^ National Park Service, White Sands National Park, P.O. Box 1086, Holloman AFB, NM 88330, USA.; ^5^ Department of Classics and Cornell Tree Ring Laboratory, Cornell University, Ithaca, NY 14853, USA.; ^6^ University of Alaska Museum of the North, Fairbanks, AK 99775, USA.; ^7^ Arizona State Museum and School of Anthropology, University of Arizona, Tucson, AZ 85721, USA.; ^8^ Department of Earth Sciences, Kent State University, 325 South Lincoln Street, Kent, OH 44242, USA.; ^9^ School of Life and Environmental Sciences, Bournemouth University, Poole BH12 5BB, UK.; ^10^ Geologic Resources Division, National Park Service, 1849 C Street NW, Washington, DC 20240, USA.

## Abstract

Expanded excavations at White Sands National Park have unearthed 20 stratigraphic horizons containing in situ human footprints, megafaunal trackways, and linear features created by travois. Advances in radiocarbon dating of conifer pollen, combined with ages of aquatic seeds, refines the chronology of the footprint-bearing sequence to between 23.5 and 21.7 thousand years ago (ka). Biomarker, pollen, and stratigraphic evidence indicate a complete overturning of the environment from a perennial lake to a playa and capture a large aridification episode coincident with Dansgaard-Oeschger event 2 at 23.2 ka, which shrank pluvial Lake Otero and allowed people to traverse the former shoreline area for nearly two millennia. This well-dated and highly resolved paleoclimate record provides compelling evidence that humans in North America experienced marked environmental changes during the Last Glacial Maximum.

## INTRODUCTION

At White Sands National Park (WHSA) Locality 2, human footprints and evidence for the earliest use of travois for conveyance provide insight into human activity in North America during the Late Pleistocene (*1*, *2*). Initially constrained to between ∼23 and 21 thousand years ago (ka) (*1*, *3*), the footprint-bearing sediments are firmly within the temporal bounds of the Last Glacial Maximum (LGM) (*4*). Although the veracity of the age estimates has been challenged (*5*–*11*), the chronology and supporting evidence remain conclusive (*12*–*15*). Renewed excavations at White Sands offer an opportunity to refine the chronologic framework of the site and establish a deeper understanding of the stratigraphic relationships, hydrologic setting, and depositional environments. They also allow for the development of highly resolved paleoenvironmental and paleoclimatic records in a terrestrial setting, including a detailed account of an abrupt drying event that occurred during the LGM. Reconstructions of temperature, hydroclimate, and plant biomes reveal the changes in environmental conditions faced by ancient humans in the Tularosa Basin of south-central New Mexico, USA, at this time.

## RESULTS AND DISCUSSION

### An extended stratigraphic sequence at WHSA Locality 2

The original trench at WHSA Locality 2 was 11 m long and exposed ∼1.3 vertical m of sediment, including ∼30 cm that encompassed seven distinct horizons containing human footprints (*1*). The trench was enlarged to a total length of 25 m (fig. S1), exposing ∼2.7 vertical m of stratigraphy consisting of dark gray gypsiferous clay overlain by fine-grained, gypsum-rich alluvium intercalated with clay and silt. This sequence represents a shallow lacustrine setting of pluvial Lake Otero that transitioned to a lake margin/wet playa environment (Fig. 1). Sedimentary units within the trench contain numerous beds and laminations ranging in thickness from a few millimeters to several centimeters and can be traced laterally with a slight (∼1° to 2°) dip from east to west. Most of the units exposed in the trench are moderately cemented with gypsum, but those enriched in dolomite, most prominently at track horizon 2 (TH2), are indurated (Fig. 1). Several horizons exhibit soft sediment deformation apparent in both planar view and cross section resulting from loading associated with megafaunal tracks or humans dragging sticks or travois consisting of a single pole or crossed poles, presumably during the transport of resources (*2*) (denoted as linear features in Fig. 1).

**Fig. 1.**
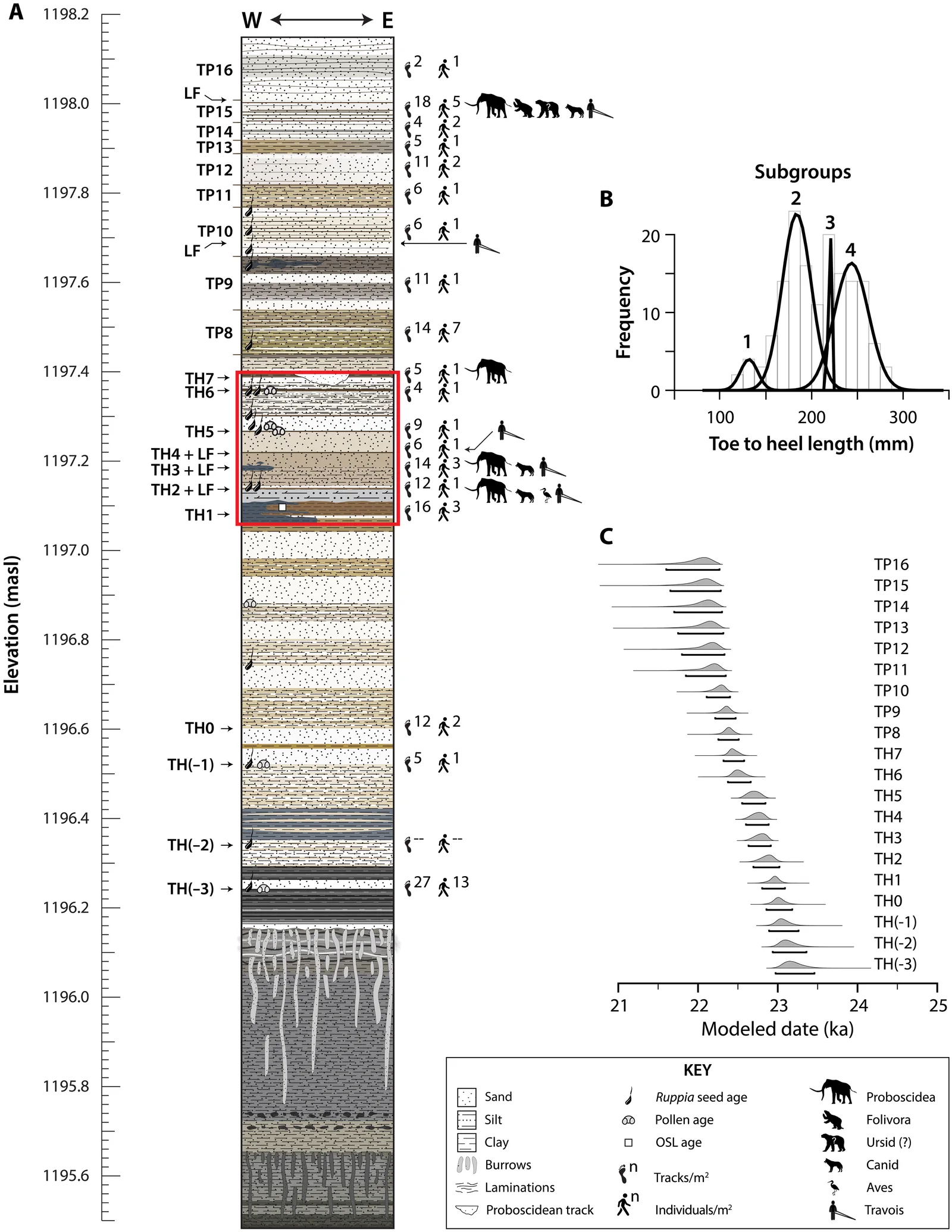
Geologic context and ichnological summary. (**A**) Composite stratigraphy, track horizons (THs) and track packages (TPs), linear features (LFs), and chronologic samples (*Ruppia* seeds, pollen, and OSL) at WHSA Locality 2. Numbers of human tracks and individuals per square meter; megafaunal tracks of Proboscidea, Folivora (ground sloth), Carnivora (canid and possible ursid), and Aves; and travois pulled by humans are also shown. The red box depicts the extent of the stratigraphy at WHSA Locality 2 reported previously (*1*, *3*). Low-quality tracks in TH(−2) were noted but not counted. (**B**) Size frequency distribution of human footprints from tracked surfaces of all ages. The four subgroups correspond to ages of (1) <4 years (*n* = 8), (2) 6.3 ± 0.1 years (*n* = 72), (3) 11.8 ± 0.2 years (*n* = 12), and (4) at least 13.8 ± 0.2 years (male) or 14.6 ± 0.2 years (female) (*n* = 60 total) (table S1). (**C**) Modeled dates (using model 4) for all THs and TPs based on the corrected ^14^C ages of the *Ruppia* seeds. This figure was generated using the Sequence function in OxCal v.4.4.4 (*57*) and the IntCal20 calibration curve (*58*).

Human footprints have been identified and documented on 20 discrete stratigraphic horizons and sedimentary packages containing multiple footprint-bearing horizons [track packages (TPs); Figs. 1 and 2]. The occurrence of footprint-bearing horizons appears to increase upward through the stratigraphic section, which is likely a function of the size and accessibility of the excavated areas. A total of 152 human footprints were measured in the gypsum-rich environment that provided ideal conditions for their preservation. Tracks of Pleistocene megafauna are also present, including Proboscidea, Folivora (ground sloth), Carnivora (canids and possible ursids), and rare Aves, as are the linear features noted above (Figs. 1 and 2).

**Fig. 2.**
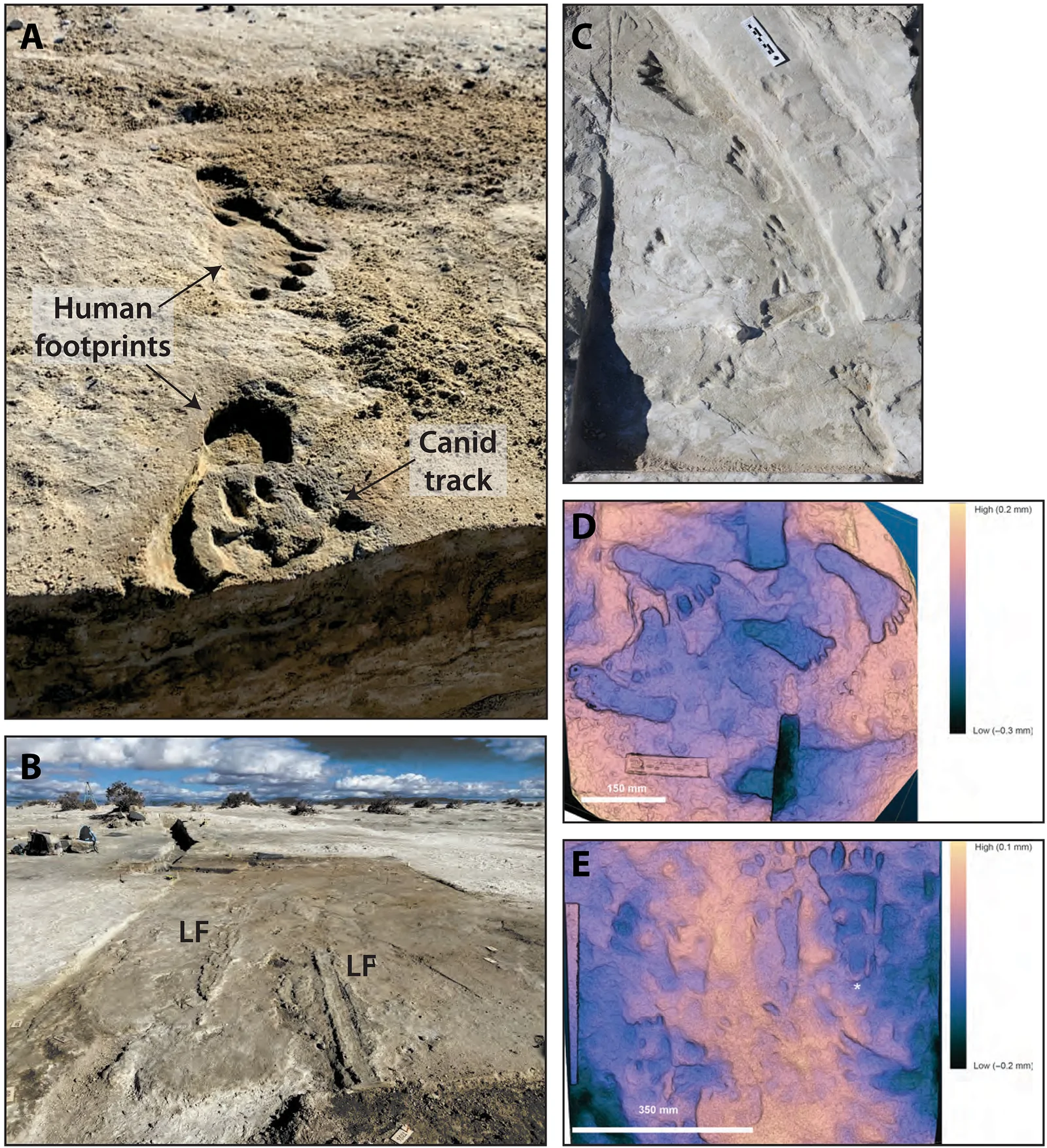
Ancient human footprints and other ichnological features. (**A**) A large canid track superimposed on a human footprint excavated on TH3. Photo Credit: K.B.S., US Geological Survey (USGS). (**B**) Linear features (LF) on TH2 that have been interpreted as travois pulled by humans, presumably during transport of resources (*2*). Photo Credit: K.B.S., USGS. (**C**) Orthomosaic of the surface of TP15 showing human footprints (Photo Credit: M.R.B., Bournemouth University). (**D**) Color rendered height map of multiple human footprints on TH0. (**E**) Color rendered height map of human footprints on TH(−3).

Biometric estimates were hampered by the small number of tracks on individual horizons, so the track data were pooled to make some general observations. Based on the size of the footprints, four age and stature subpopulations emerge, including three consisting of children [<4 years (*n* = 8), 6.3 ± 0.1 years (*n* = 72), and 11.8 ± 0.2 years (*n* = 12)] and a fourth subgroup (*n* = 60) that are either adults or adolescents that are at least 13.8 ± 0.2 years (male) or 14.6 ± 0.2 years (female) (Fig. 1B) (table S2). There is no evidence for congregation or habitation at WHSA Locality 2, and the travois features support the hypothesis of a transit site.

### A refined chronology for WHSA Locality 2

The temporal span of footprint-bearing sediments in the original trench, specifically from TH2 to TH6, was established previously using radiocarbon dating of seeds of *Ruppia cirrhosa* (*n* = 11) and conifer pollen (*n* = 3), as well as optically stimulated luminescence (OSL) dating (*n* = 3) (*1*, *3*). The seed, pollen, and OSL ages were all statistically indistinguishable from one another and are supported by the available geologic, hydrologic, stratigraphic, ecological, and climatic evidence (*1*). However, dating uncertainties of more than two millennia for the pollen that were related to the radiocarbon procedural blank limited our ability to quantify the magnitude of potential carbon isotope biases in the sequence and further resolve the chronologic framework.

To counter this, we established a new procedural blank using old (>110 ka) pollen from the Ziegler Reservoir fossil site in Snowmass Village, Colorado (*16*). We also developed and refined laboratory protocols to concentrate and pretreat the pollen grains and used flow cytometry to isolate up to ∼75,000 pollen grains per sample from residual organic matter. Together, these methodological advances reduced the levels of modern carbon introduced during laboratory processing significantly, such that the ^14^C content of the Ziegler Reservoir pollen is commensurate with other types of blank materials commonly used in radiocarbon dating. In turn, the uncertainties of pollen ^14^C ages at WHSA Locality 2 were reduced fivefold, which allowed their use in evaluating the accuracy of the *Ruppia* seed ages.

Eleven new calibrated ^14^C ages of *Ruppia* seeds were obtained that range from 24.87 ± 0.34 ka to 21.58 ± 0.33 ka as well as three new ^14^C ages of conifer pollen from the stratigraphically lower part of the record that span from 23.44 ± 0.43 ka to 22.71 ± 0.33 ka (Fig. 1) (table S4). The new pollen ages are consistently younger than *Ruppia* seed ages at the same stratigraphic levels, by as much as ∼1330 ^14^C years, which is likely the result of hard-water effects (HWEs) that influenced the ^14^C content of the *Ruppia* plants in the lower part of the sedimentary sequence.

The prospect of large HWEs in the WHSA Locality 2 record has been previously debated (*11*, *14*), and it is well-known that HWEs vary over time and space (*17*). We developed four different age models, including two that used the measured ^14^C activities of the new pollen samples to correct the *Ruppia* seed ages for HWEs (model 1: *Ruppia* seeds, uncorrected; model 2: *Ruppia* seeds/pollen, uncorrected; model 3: *Ruppia* seeds, constant HWE correction; and model 4: *Ruppia* seeds, variable HWE correction). We then used the strong climate teleconnection between the North Atlantic and southwestern US during the Late Pleistocene (*1*, *18*–*20*) to test the accuracy of the models by comparing leaf wax hydrogen isotope (δ^2^H_wax_) data from WHSA Locality 2 to the Greenland ice core oxygen isotope (δ^18^O) record (*21*), which show a close correspondence (Fig. 3B).

**Fig. 3.**
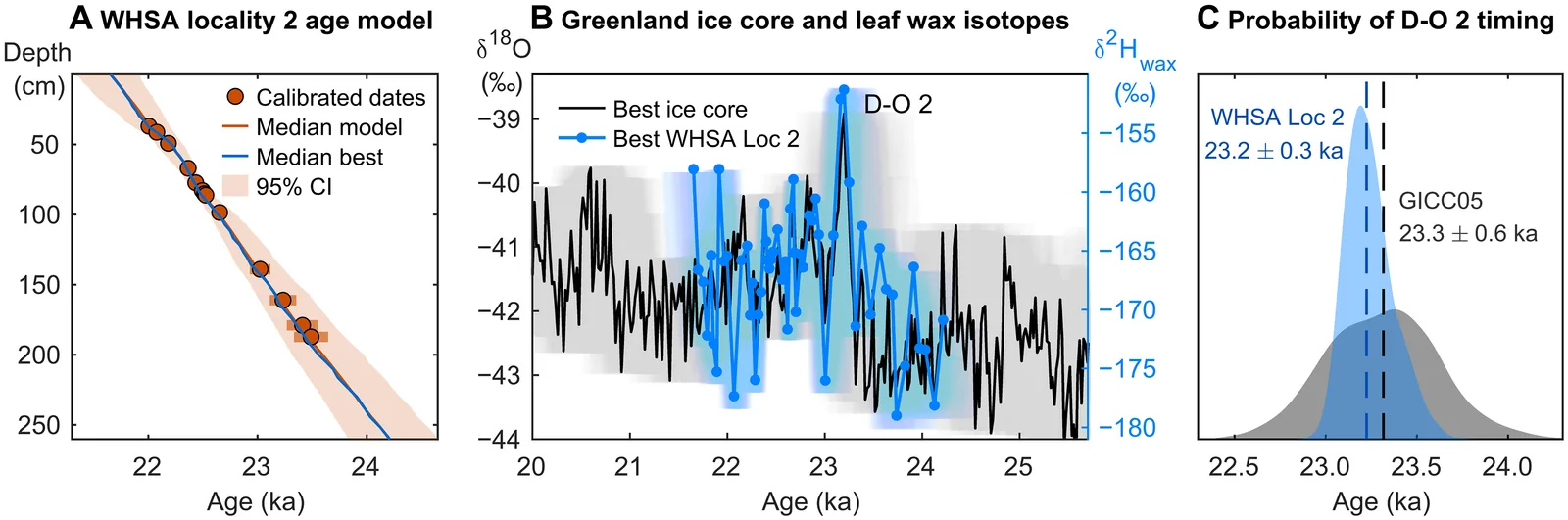
A refined chronology for WHSA Locality 2. (**A**) Age-depth plot using model 4, which is based on *Ruppia* seed ages corrected for variable HWEs using the ^14^C activity of paired pollen samples. Calibrated dates and 2σ uncertainties are shown as dark orange dots with error bars. The median model is shown as a dark orange line, with the 95% confidence interval (CI) in lighter orange. The blue line corresponds to the median model for the subset of age models that produce a correlation with the Greenland Ice δ^18^O record of *r* > 0.6 (denoted as “best”). (**B**) Greenland ice core δ^18^O (the average of the GISP2 and NGRIP records, on the GICC05 chronology, in black) and WHSA Locality 2 δ^2^H_wax_ data (blue). Both records are plotted on their median “best” chronology. Shading represents the age uncertainty associated with the records, with darker shading corresponding to greater probability. (**C**) Kernel density estimates of the probability distribution for the timing of Dansgaard-Oeschger event 2 (D-O 2) as recorded in Greenland ice δ^18^O (in black) and WHSA Locality 2 δ^2^H_wax_ (in blue). The blue dashed line denotes the median age for D-O 2 according to WHSA Locality 2; the black dashed line shows the median age for D-O 2 according to the GICC05 ice core chronology.

Model 4 yielded the strongest correlation [correlation coefficient (*r*) = 0.40, *P* = 0.03, accounting for serial correlation; (*22*)] (fig. S20). This model incorporates variable HWEs where the magnitude of the correction is greatest at the transition from lacustrine to alluvial sediments and decreases up section, which agrees with lithologic and geochemical data (Fig. 1) that show that pluvial Lake Otero receded due to drier and warmer conditions. The HWEs declined and eventually dropped to a negligible amount at TH1 as the environment became dominated by overland flow, intermittently wet conditions, and alluvial sedimentation.

To further evaluate the accuracy of model 4, we examined a prominent peak near 23 ka in the δ^2^H_wax_ data that occurs contemporaneously with warming during Dansgaard-Oeschger event 2 (D-O 2) (Fig. 3B) (*21*) while taking uncertainties in both records into account; for model 4, the age uncertainties average ∼250 years (2σ), whereas the GICC05 ice core chronology has a maximum counting error of ∼500 to 600 years (2σ) during this interval of the LGM (*21*). We then generated ensembles of possible age models from WHSA Locality 2 and GICC05, respectively, and computed the correlation between δ^2^H_wax_ and δ^18^O to assess how well the two series match within their chronological errors. Approximately 90% of the resulting iterations have a positive correlation, and 25% of the iterations yield an *r* value greater than 0.35, indicating that a positive relationship between the two datasets is likely. For some combinations, the correlation exceeds 0.6 (shown as the “best” model in Fig. 3B); these matches originate from sliding the GICC05 chronology forward in time by ∼100 years (Fig. 3B). All age model iterations from model 4 fit within the temporal envelope of D-O 2 (Fig. 3C) supporting the interpretation that the δ^2^H_wax_ peak reflects conditions during this event. Because the age model uncertainty at WHSA Locality 2 is smaller than the GICC05 chronology, it potentially provides a more precise and accurate age estimate of 23.2 ± 0.3 (2σ) ka for D-O 2 (Fig. 3C).

The presence of D-O 2 in the δ^2^H_wax_ data shows that there is little chance that the HWEs are larger than we estimate because there are no other comparable isotopic enrichment events in the Greenland ice cores within the age model uncertainty or, for that matter, for the several millennia surrounding the White Sands sedimentary sequence (Fig. 3B). We conclude that multiple lines of evidence constrain the age range of the human footprints at WHSA Locality 2 to between 23.5 and 21.7 ka (Fig. 1C).

### The climate and environment of White Sands during the LGM

The new chronology of the WHSA Locality 2 sedimentary sequence allows us to investigate the climate and environmental changes that humans experienced at a high temporal resolution (time steps between 20 and 80 years, with an average of 50 years), which is rare for glacial-aged paleoclimate archives from the southwestern US.

We took a multiproxy approach to elucidate climate and environmental changes on local, extralocal, and regional spatial scales, combining the sedimentary evidence with analyses of biomarkers, pollen and ostracode assemblages, and mineralogy. The biomarker data include (i) the previously discussed δ^2^H_wax_ data, which assisted with age modeling but also provide climate information by recording the hydrogen isotopic composition of precipitation (δ^2^H_p_) (*23*); and (ii) the relative abundances of glycerol dialkyl glycerol tetraethers (GDGTs), membrane lipids produced by archaea and bacteria. GDGTs can be used to infer air temperature via the MBT′_5Me_ proxy (*24*, *25*), relative changes in the salinity of the lake/playa through the abundance of GDGTs associated with saline conditions (*26*, *27*), and the basin depositional environment through the use of the BIGMaC machine learning algorithm (*28*). The biomarker and pollen data share similar trends, suggesting that they provide a coherent view of environmental change. To highlight the primary environmental changes in this multiproxy dataset, we performed principal components analysis (PCA) of the GDGT indices and prominent pollen groups (fig. S25). This analysis isolates two interpretable modes of climate change: one (PC1) that describes a long-term trend in temperature and moisture balance (driven by evaporation) of the basin (Fig. 4A), and a second (PC2) that captures shorter, centennial-scale changes in precipitation/aridity (Fig. 4B). Combined with stratigraphic evidence and ostracode assemblages, these modes provide a detailed picture of the evolution of climate and the environment when humans traversed White Sands.

**Fig. 4.**
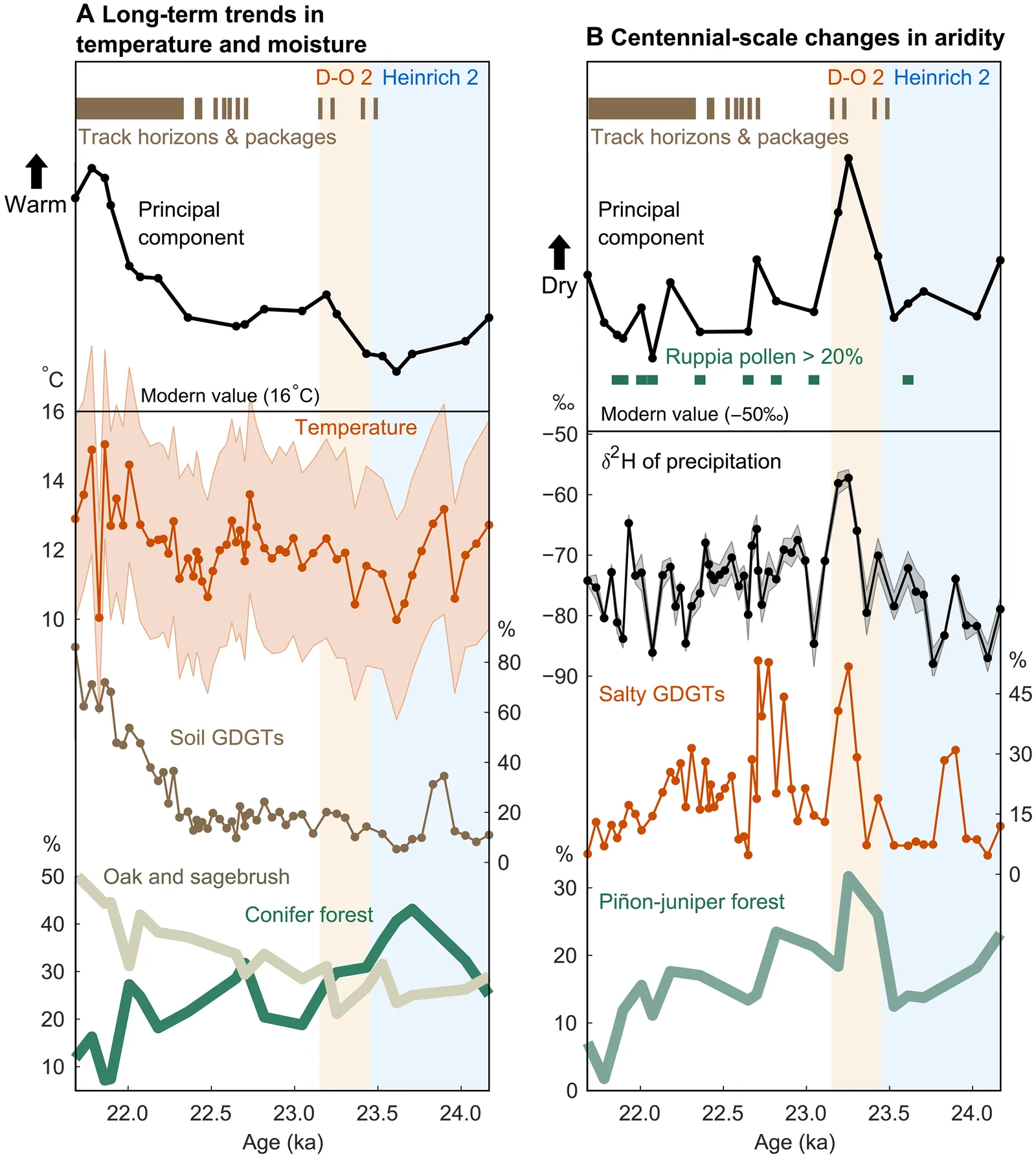
Glacial climate and environment at WHSA Locality 2. A PCA was undertaken to summarize the pollen and biomarker data. (**A**) The leading mode of variability (PC1) captures long-term warming and drying of Lake Otero in response to higher temperatures and increased evaporative demand. In addition to the principal component, we show data that loads prominently on this mode, including brGDGT-based mean annual air temperature (with modern value plotted for reference), the relative abundance of soil GDGTs, and the trade-off between mixed conifer forest (*Pinus*, *Abies*, and *Picea*) and oak/sagebrush steppe pollen. (**B**) The second mode of variability (PC2) captures short-term fluctuations in aridity. Data that load on this mode include the percentage of *Ruppia* pollen, δ^2^H of precipitation (inferred from δ^2^H_wax_), the relative abundance of salty GDGTs, and rapid shifts in piñon-juniper forest pollen. Note that the principal components have arbitrary units, so the *y* axis values are not indicated.

The leading principal component of our analysis (PC1; 39% of the variance; fig. S25) describes a long-term gradual shift in the depositional environment from a deeper lake to shallower water body that is supported by the lithology of the sedimentary sequence (Fig. 4A). Between ∼24.2 and 23.6 ka, Lake Otero covered WHSA Locality 2 based on the presence of dark-colored, clay-rich gypsiferous sediments that represent a lacustrine depositional setting and vertical cylindrical burrows with distinct linings (*Skolithos* ichnofacies) that are indicative of sandy, shallow lacustrine or fluviolacustrine environments (Fig. 1) (*29*). High amounts of GDGTs that are classified by machine learning as coming from a lake environment and low amounts of GDGTs classified as being from soils (“soil GDGTs”), which load prominently on PC1, support the continued presence of standing water (Fig. 4A). Ostracodes in this stratigraphically lowest part of the record consist of two distinct assemblages, including *Limnocythere staplini* alone and *L. staplini* with *Cyprideis salebrosa* and the foraminifera *Cribroelphidium* sp. cf. *gunteri*, which indicates that the water of Lake Otero was oligohaline to saline, rich in sulfate, and depleted in bicarbonate (low Alk/Ca) (fig. S26). Pollen suggests the mountainous areas around the lake hosted a mixed conifer forest, reaching 40% of the total and dominated by *Pinus* sp. (Fig. 4A). Mean air temperatures (based on the MBT′_5Me_ proxy) during this time interval were ∼12° or ∼4°C colder than modern mean annual conditions (Fig. 4A).

Starting around ∼23.6 ka, the lithologic transition from lacustrine gypsiferous clays to alluvial silts and sands indicates that Lake Otero began to shrink and recede to the west of WHSA Locality 2 as temperatures began to rise (Fig. 4A), in agreement with an independent geomorphic record to the northwest (*12*). As the region warmed, the mixed conifer forest on the piedmont slopes declined and was replaced by an oak and sagebrush ecosystem (Fig. 4A). The warming accelerated at 22.4 ka and was accompanied by a rise in soil GDGTs (at the expense of GDGTs that are classified as coming from a lake), indicating the basin began drying up. The lithologic changes and geochemical data also reflect this shift, with sediments in the upper part of the record exhibiting high concentrations of gypsum, bassanite, and minor halite, a decrease in calcite and aragonite concentrations, and the absence of microfauna and/or invertebrate trace fossils (Fig. 1). These data demonstrate that moisture availability declined because of a temperature-driven increase in evaporative demand.

The late glacial warming at White Sands differs from the average evolution of temperature change in the Northern Hemisphere, which shows progressively colder conditions from ∼23 to 18 ka with deglacial warming occurring thereafter (*30*). In contrast, conditions at WHSA Locality 2 resemble sea surface temperature alkenone data from the California Margin, which show warming began as early as 26 ka that may represent a weaker California current in response to a reorganization of the wind systems by the Laurentide Ice Sheet (*31*). Our data suggest that early glacial warming recognized along the California coast might have been widespread in the southwestern US, contributing to the relatively mild temperatures (12° to 14°C) encountered by early humans at White Sands.

The second principal component of our paleoenvironmental data (PC2; 20% of the variance; fig. S25) captures shorter-term fluctuations in precipitation/aridity that are overprinted on the longer-term warming and drying trend (Fig. 4B). The proxies that share variance on this mode include δ^2^H_p_, GDGTs that are classified as “marine,” which we interpret to represent saline conditions [“salty GDGTs”], *Ruppia* pollen, and piñon-juniper pollen (fig. S25). From 24.2 to 23.6 ka, this mode indicates wet conditions, as indicated by lower percentages of salty GDGTs and depleted values of δ^2^H_p_ [∼−80 per mil (‰), which is 30‰ lower than modern values; Fig. 4B]. This time period coincides with Heinrich Event 2, a millennial-scale cool period in the North Atlantic (*32*) that is associated with increased precipitation in the southwestern US (*33*). Climate model simulations of Heinrich events show that cooling in the North Atlantic resulted in more atmospheric river events reaching the western US during winter (*33*, *34*). The isotopically depleted moisture reaching Lake Otero (as shown by δ^2^H_p_) is consistent with winter precipitation originating from the Pacific that experiences more rainout and less evaporation in a cooler climate (*35*). Although White Sands sits in the North American Monsoon (NAM) domain today, summer rain is isotopically enriched, and, thus, it is unlikely to explain the depleted values that we observe during Heinrich Event 2. Moreover, during the last glacial period, the NAM was weak (*36*), and so LGM isotopic records in New Mexico are generally interpreted to reflect winter rainfall dynamics (*37*).

At 23.2 ka, extreme but short-lived aridity prevailed at White Sands, as well as other localities in the southwestern US [e.g., (*20*, *38*, *39*)] in response to D-O 2. This event is expressed in the δ^2^H_p_ data as a sudden enrichment in the isotopic composition of rainfall, which we infer is associated with less winter rainfall and perhaps more evaporation during moisture transport (*35*). A spike in salty GDGTs indicates that the basin became more saline (Fig. 4B), and a rise in piñon-juniper pollen shows that this more drought-tolerant ecosystem expanded during the event. D-O 2 also marks a permanent change in the environment from a shallow lake to a lake margin/wet playa setting. Beginning at this time, humans and megafauna in the Tularosa Basin were able to travel across a newly exposed mosaic of wet and dry ground (*1*); the earliest track horizon [TH(−3)] at WHSA Locality 2 occurs during D-O 2 (Figs. 1 and 4).

After D-O 2, another centennial-scale event occurred at ∼22.7 ka, where the presence of gypsiferous clays indicates short-lived and localized ponding, which was followed by fluctuating salinity and evaporative conditions that resulted in the distinct indurated dolomite horizon at TH2 (Fig. 1) (*40*). Another spike in salty GDGTs occurred here, supporting the lithologic interpretation of more saline conditions (Fig. 4B). Throughout the stratigraphic sequence, the abundance of *Ruppia* pollen shows a close correspondence with the second principal component. Peaks in *Ruppia* pollen (>20%, reaching as high as 60%) mark times when there was sufficient overland flow of water to sustain growth of this emergent aquatic plant and correspond to more depleted δ^2^H_p_ and a reduction in salty GDGTs, or, in other words, centennial-scale wet periods (Fig. 4B).

Previous studies have identified abrupt shifts in paleoclimate and environmental conditions in the Chihuahuan Desert that occurred during the LGM (*41*–*43*), including D-O events (*37*). However, the terrestrial record at WHSA Locality 2 provides the first multiproxy climate record that exhibits both long-term (millennial-scale) trends and superimposed short-term (centennial- to decadal-scale) fluctuations, including the only quantitative temperature estimates for this region of the American Southwest. It also marks the first time such estimates have been developed at high temporal resolution for a landscape that was traversed by both humans and megafauna in North America.

Our findings indicate the glacial climate at White Sands was dynamic. Humans witnessed a complete floral turnover from conifer forest to sagebrush and oak steppe, the recession of pluvial Lake Otero, and centennial-scale swings in hydroclimate, including severe aridity during D-O 2. Throughout, people continued to visit the basin, which is a testament to the importance of the vital resources that were available there. This large yet waning pluvial system was a destination that was visited repeatedly for nearly two millennia by local residents and/or people migrating through the basin. The expanded White Sands record advances our understanding of the conditions that early humans in the region experienced in the face of a rapidly changing hydroclimate and provides new insights into the environmental changes that occurred during individual human lifetimes at the height of the LGM.

## MATERIALS AND METHODS

### Excavation methods

Initial excavations at WHSA Locality 2 were conducted in January 2020 as described in detail by Bennett *et al.* (*1*). At that time, the main east-west trench was cut perpendicular to the slope using a mini trencher (dirt-rated chainsaw) coupled with manual block removal. Overall, the trench was 11 m long and exposed 1.3 vertical m of the stratigraphic sequence (fig. S1). A small north-south trench was also cut in January 2020 using the same procedures. Blocks of sediment in both trenches broke along natural bedding planes, which facilitated their removal while also exposing surfaces that contained human footprints and megafaunal tracks. Loose sediment from the excavations was sieved, but no artifacts were recovered.

Additional excavations were completed in January 2022 during which the main trench was extended to a total length of 25 m, exposing nearly 2.7 m of vertical stratigraphy (figs. S1 and S2). As in 2020, parallel cuts using a dirt-rated trenching tool were used to define the lateral dimensions of the trench, and cross-cut incisions assisted in block removal. Where possible, blocks were cut on four sides vertically and then levered upward using a pry bar (fig. S3), which exposed bedding planes that often contained human footprints.

In the upper part of the stratigraphy near the eastern end of the main trench, potential footprint horizons were also exposed using other methods (fig. S4). In some cases, this involved lowering the existing surface to reveal tracks adjacent to the trench. In other places, side benches were exposed by first using a circular rock saw to define vertical blocks, which were then hinged upward (fig. S3E) using a pry bar. The newly exposed surfaces were then brushed clean, and track topographies were excavated with wooden picks and brushes.

Outlines of footprints and tracks at WHSA Locality 2 are typically visible on freshly exposed surfaces via slight color differences and subtle contrasts in grain sizes. In many places, tracks are present on hard gypsum-rich layers and were revealed simply by removing the overlying silt and fine sand. Finally, as in 2020, loose sediment from the extended excavation was sieved, but no artifacts were recovered.

Figures S5 and S6 detail the north-facing wall of the upper and middle reaches of the main east-west trench, as well as the south-facing wall of the lower reach of the trench. Each of these walls was cleaned thoroughly in preparation for stratigraphic analysis, sampling, and photodocumentation.

### Ichnology

We have identified 20 distinct sedimentary horizons or packages that contain human footprints, including four horizons [TH0 through TH(−3)] that are stratigraphically below the previously documented tracks (table S1 and figs. S7 to S14). Footprint-bearing surfaces in the lower and middle parts of the trench continue to be designated as “track horizons” because they are isochronous surfaces that represent a moment in time, whereas we designate “track packages” (TPs) in the upper part of the sequence (above TH7) because footprints impress across multiple thin layers, many of which also contain tracks, and consequently assigning tracks to a specific horizon becomes problematic (fig. S15). The thickness of each TP was assigned on the basis of the thickness of the corresponding stratigraphic unit. Overall, the numbering system is consistent with that reported by Bennett *et al.* (*1*) and Pigati *et al.* (*3*), which allows for continuity between studies but necessitated using negative numbers for the lowest three track horizons, TH(−1) through TH(−3).

Best excavation practices were followed as set out by the ichnological community (*44*, *45*). Where possible, tracks were captured in three dimensions using between 40 and 150 oblique photographs taken with a Canon EOS 1200D and fixed lens or with an iPhone 12/15. Orthomosaics and digital elevation models were generated with Agisoft Metashape Pro v.1.8.2 (www.agisoft.com), with subsequent processing in Meshlab (www.meshlab.net) and CloudCompare (v2 12.4, www.danielgm.net/cc). Individual models were scaled and rotated to the principal plane. Color rendering was undertaken in Cloud Compare (v2 12.4, Kyiv; www.danielgm.net/cc) having first applied a 2.5D Delaunay triangulation. Autorotation of surfaces to the orthogonal plane was undertaken in all cases.

Footprint lengths were determined from heel to first toe unless otherwise stated, and all measurements were made on the basis of the three-dimensional models. Inferences from footprints such as stature and track-maker age all follow best practice via methods described previously by Bennett *et al.* (*1*, *46*). We also report minimum number of individuals (MNI) using a modification of the method established by Webb *et al.* (*47*) and further developed by Bennett and Reynolds (*48*) using the equation

$$\text{MNI}=\frac{R}{\left(\text{SITV}*1.96*2\right)}$$

where *R* is the range of sizes of similar age on a tracked surface and SITV is a measure of the SD of intratrackway variance, which has been estimated to be 6.67 based on experiments by Webb *et al.* (*47*) and revised to a value of 9.52 by Belvedere *et al.* (*49*). This value varies between individuals, walking speeds, and substrates and is difficult to determine quantitatively. In this study, we used a total of 53 estimates of intratrackway variance based on the values compiled by Belvedere *et al.* (*49*). From this, we computed an average *r* value of 8.34 ± 1.80 (1 SD). We then used this average to bootstrap (*n* = 1000) values for *SITV* and calculate the MNI for each of these values. This was repeated 1000 times to determine the average, median, and quartile ranges for MNI.

We report the average MNI rounded upward to a whole number. Note that values for MNI and the number of tracks (T) are normalized to the excavated surface area of each track horizon. However, some of the areas are small with high track density, so it is possible that we simply sampled a “trail” or other track concentration. We caution, therefore, that the MNI values reported here should be viewed as crude approximations only and should not be used for population estimates.

The number of tracks on some of the excavated horizons is small, so it is not appropriate to make specific biometric estimates for the purpose of comparing different track horizons. We instead pooled track data (*n* = 152) to make some general observations. An additional 22 tracks were identified (table S1) but were not included in the analysis because they were not contiguous with the trench. Inferences of stature from footprints were made using the 15% value (walking, firm substrate) after Wiseman and De Groote (*50*). Age estimates of the humans who made the tracks (table S2) were based on data from UMTRI/CPSC Child Anthropometry Study (*51*), supplemented by observations from one of the authors own dataset (*n* = 3901; MRB). For each whole number foot length in this dataset, the range of associated subject ages was used to generate a SD and mean from which bootstrapped samples were generated (*n* = 1000). Despite this, the number of values in some size classes is small or zero. To counter this problem, we used sampling windows of −3, 5, and 7 size classes (i.e., 1, 2, and 3 classes ± a given foot size class). Differences in mean values were negligible, but, in all cases, the error margins were smaller when using a larger sampling window. The mean ages and errors used here are all based on a five-column window (i.e., foot size ± 2 mm). It is important to recognize that a range of studies recognize differences in footprints and foot lengths [e.g., (*52*)], often by as much as 5%. Intratrackway variability may introduce additional uncertainty, so, as above, trackmaker age estimates should be taken as approximations only.

### Radiocarbon dating

#### Samples

Previous studies have reported calibrated ^14^C ages of *Ruppia* seeds (*n* = 11) and pollen (*n* = 3) at WHSA Locality 2 (*1*, *3*). For the current study, we collected additional samples of *Ruppia* seeds (*n* = 11) and pollen (*n* = 3) for radiocarbon dating from numerous horizons within the extended sedimentary sequence. Notably, two of the new pollen samples, 22WHSA1-18.1nn and 22WHSA1-18.1rr, were collected from the same stratigraphic horizons as two of the new *Ruppia* seed samples, 22WHSA1-18.1n and 22WHSA1-18.1r, respectively. In addition, we determined the ^14^C content of five aliquots of sediment from the Ziegler Reservoir fossil site in Snowmass Village, Colorado, that are beyond the limit of radiocarbon dating for use as a procedural blank for pollen (see below).

#### Chemical pretreatment

*Ruppia* seeds recovered from sediment samples were physically cleaned and treated at the US Geological Survey (USGS) Radiocarbon Laboratory in Denver, CO, using the standard acid-base-acid (ABA) procedure. The ABA treatment included an initial acid wash with 1 M HCl at 60°C for 30 min to remove carbonate minerals, a base wash with 1 M NaOH at 60°C for 30 min to remove humic acids and other base-soluble compounds (this step was repeated until the supernatant remained clear), and a final acid wash in 1 M HCl at 60°C for at least 10 min to acidify the sample. The treated samples were washed with ASTM Type 1, 18.2-MΩ water and dried.

Samples of pollen from WHSA Locality 2 were also treated at the USGS Radiocarbon Laboratory. All of the procedures described below took place either in a positive flow hood (when no chemicals were involved) or a standard fume hood (when chemicals were used). Clean and/or new beakers and tubes were covered throughout the entire process to prevent the introduction of modern pollen. For each sample, 75 to 150 g of sediment was initially weighed and placed into glass beakers and covered with 18.2-MΩ ultrapure water (hereafter UPW). The samples were then stirred and were allowed to soak for at least 24 hours.

The samples were then stirred again, and mineral material was allowed to settle before being poured through a clean 150-μm sieve. This step was repeated with decreasing settling times (30, 20, 10 s, etc.) until the supernatant slurry was either clear or nearly clear before passing through the sieve. Material that was caught on the screen was gently macerated with a sterile stir rod and rinsed with UPW. The sample was stirred and allowed to settle for up to 30 s, depending on the density of the slurry, and then transferred to another beaker, which further facilitated separation of organic material from mineral particles based on density differences.

Slurries in the final beakers were transferred into 250-ml Teflon beakers to aid in the distribution into multiple 50-ml polytetrafluoroethylene (PTFE) centrifuge tubes (to prevent the sediment volume from exceeding ∼7 cm^3^). The samples were centrifuged using an IEC tabletop centrifuge, at a setting of 3, for 3 min and decanted. This setting has been tested repeatedly in the USGS laboratory to ensure the settling of most pollen grains while allowing particles smaller than ∼8 μm to remain in the supernatant fluid. This protocol has been verified multiple times by microscopic inspection.

The samples were then mixed using a vortex mixer with UPW and again centrifuged and decanted. This step was performed as many times as necessary to facilitate the removal of ultrafine material (i.e., particles less than 8 μm in diameter). The total volume for each sample was estimated and recorded. The samples were then vortexed and slowly treated with ∼5 ml of 1 M HCl to remove carbonates and the intensity of the reaction noted. This step was repeated until the visible reaction stopped. After most of the carbonates were removed, 10 to 15 ml of 6 M HCl was added, the tube was capped lightly, and the samples were allowed to sit overnight at room temperature. The samples were centrifuged and decanted, and fresh 6 M HCl was added followed by a brief vortex to mix and placed in a heating block at 80°C for at least 4 hours, and then again cooled overnight in the HCl.

Following the HCl treatment, the samples were again centrifuged and decanted, and a few milliliters of concentrated HF was introduced to begin removing silicates. The reaction was closely monitored, and, once it was determined that the reaction was not violent and the samples would not boil over, an additional 5 to 10 ml of concentrated HF was added to each sample and allowed to sit in HF overnight. The samples were centrifuged and decanted, and ∼10 ml of fresh, concentrated HF was added and then placed in the heating block at 80°C for at least 2 hours. The samples were centrifuged and decanted, and 6 M HCl was added to prevent the precipitation of fluoride minerals. The samples were placed in a heating block at 80°C for at least 2 hours, then centrifuged and decanted, combined in steps down to one tube (more if the remaining sediment volume was greater than 7 cm^3^), centrifuged, decanted, and washed with UPW.

After the washing step was complete, the Schulze procedure was performed that included ∼3 ml of saturated KClO_3_ added to the samples and vortexed to mix and an additional 3 ml of KClO_3_ to rinse the sides of the tubes. Then, ∼6 ml of concentrated HNO_3_ was added to the tubes. The samples were allowed to react for roughly 10 min in the heating block at 80°C. This duration was determined through previous experimentation to effectively attack nonpollen organics while leaving coniferous pollen intact. UPW was then added to fill the tubes, and the samples were centrifuged and decanted. Each sample was then washed at least three times with UPW.

After this wash step was complete, 4 ml of freshly made 10% KOH was added to remove base-soluble organics, and an additional 4 ml 10% KOH was used to rinse the sides of the tubes and then placed in the heating block at 80°C. The base reaction took place for a total of 10 min to remove soluble materials without deteriorating the coniferous pollen exine. The samples were then topped with UPW, centrifuged, and decanted. Each sample was then washed with UPW at least three times, more if color remained in the supernatant fluid. A small aliquot of each sample was examined to determine the effectiveness of the Schulze-base wash treatment. If the amount of nonpollen organic material was still too great (∼50%), then a second, shorter Schulze-base wash step was performed. This was determined for each sample separately and generally a less than 3-min treatment was used.

After the demineralization and the organic attack, each sample was typically between 1 and 3 cm^3^ in volume. This small size allowed the use of pluriSelect microsieves to be used with the 50-ml tubes. The samples were passed through dedicated sieves at 100 μm and then at 70 μm. The 70- to 100-μm fraction was collected in a 15-ml tube while the <70-μm fraction was then passed through either a 30-μm sieve (samples 22WHSA1-18.1nn and 22WHSA1-18.1rr) or a 40-μm sieve (sample 22WHSA1-18.1hh). These smaller fractions were then collected in a 15-ml tube, and the <30- and <40-μm fractions were discarded. Each sample was cycled through the sets of sieves several times to ensure removal of the smaller-than particles for each fraction.

The samples, now typically less than 1 cm^3^ each, were examined under magnification that revealed a lack of abundant pollen in the 70- to 100-μm fractions so these aliquots were not treated further. The 30- to 70-μm and 40- to 70-μm fractions contained abundant pollen and were treated using the standard ABA treatment as described above. These samples were centrifuged, decanted, and washed with UPW water at least three times, and 1 ml of 1 M HCl was added to acidify the samples during shipping.

#### Flow cytometry

At the Flow Cytometry Core Facility at Indiana University, flow cytometry analysis and sorting were performed on dissociated samples using a COPAS SELECT large particle sorter (Union Biometrica, Holliston, MA) with Advanced Acquisition package, using a 488-nm 15-mW laser excitation source. The flow cytometry method is used to detect both light scatter and fluorescence characteristics of particles using laser excitation in a fluidics-based system. These characteristics (scatter and fluorescence) are used to separate the pollen (autofluorescence) portion of the sample from other materials (nonfluorescent). Samples were received at a final volume of 10 ml per sample. Samples were briefly analyzed by light microscopy to determine how much pollen versus other materials were in the sample. Before sorting, the instrument pressure was checked to ensure that it was in the correct range. Samples were then added to UPW water in a 250-ml sample cup and acquired. A gate region for pollen was first selected using time of flight (ToF; relative length) versus extinction (EXT; relative structure) parameters. A sort region was selected on the basis of pollen autofluorescence, which is detected in the RED parameter at an emission of 610/20 nm, versus ToF. A test was performed to analyze whether the correct number of particles (maximum, 75,000) was sorted and whether the collected particles were pollen. The samples were then sorted into sterile 10-cm petri dishes, transferred to 15-ml Falcon tubes, allowed to settle, and pooled. Pooled samples were transferred to sterile CryoClear vials (Globe Scientific). One drop of 1 M HCl was added to each sample before they were frozen and shipped to the USGS Radiocarbon Laboratory on dry ice. These last two steps, addition of 1 M HCl and dry ice shipment, minimize the potential for atmospheric carbon contamination. The resulting samples consist entirely of pure pollen grains based on visual inspection.

#### Carbon extraction, graphitization, and AMS analysis

The treated *Ruppia* seeds and pollen extracts were processed at the USGS Radiocarbon Laboratory using an automated carbon extraction and graphitization system (CEGS). The *Ruppia* seeds were dried before processing, whereas the pollen aliquots were transferred to the combustion tubes while still wet to minimize the potential for loss due to static electricity during transfer. The *Ruppia* samples were initially heated on the CEGS to 150°C for 15 min under vacuum to drive off atmospheric gases, whereas the pollen aliquots were freeze-dried under vacuum before the water was driven off via sublimation and then heated to 150°C for 15 min. After this preheating step, all samples were combusted online at 625°C in the presence of excess high-purity oxygen. Water and other contaminant gases were removed by precise cryogenic separation at −140°C using a variable temperature trap capable of holding temperatures to within 1° to 2°C of the desired target. Sulfur was removed by heating the resulting gases at 400°C for 20 min in the presence of iron powder, which produces iron sulfide without fractionating the carbon isotopes. The purified CO_2_ gas was then measured manometrically, converted to graphite using an iron catalyst and hydrogen reduction (*53*), and submitted for AMS ^14^C analysis at the National Ocean Sciences Accelerator Mass Spectrometry facility in Woods Hole, MA.

#### Blank correction

Corrections of the measured carbon isotopic ratios are required in radiocarbon dating because a small amount of contaminant ^14^C (usually modern) is introduced to samples during chemical pretreatment, CO_2_ extraction, graphitization, and/or the AMS measurement (*54*–*56*). The magnitude of this “blank correction” is determined by measuring the ^14^C content of material that is independently known to be beyond the limit of the dating technique (>50 ka) so that any measured ^14^C represents atoms that were introduced during laboratory processing and measurement rather than ^14^C derived from the material itself. Two key requirements for blank corrections are as follows: (i) the type of blank material must be as close as possible to the sample material being dated, and (ii) the blank material must be processed in the laboratory using the same procedures as the unknown samples. This is especially important in the case of dating pollen because the extensive laboratory pretreatment procedures that are required to date pollen are far beyond what is typically necessary for dating other types of organic materials. The appropriate blank material for radiocarbon dating of pollen, therefore, is ^14^C-free pollen extracted from a sample using the same procedures as those used to process the samples of unknown age.

The blank material used in this study was pollen recovered from unit 8 at the Ziegler Reservoir fossil site at Snowmass Village, Colorado. Previous work has constrained the age of this unit to between 120 ± 9 ka and 109 ± 7 ka (*16*), which is well beyond the practical limit of radiocarbon dating. The blank material was treated in the same manner as the pollen samples from WHSA Locality 2, including the use of flow cytometry. The average measured F^14^C for five aliquots of the Ziegler Reservoir pollen was 0.0023 ± 0.0023 (table S3), which is five times lower than the blank level used to date pollen previously at WHSA Locality 2 (*3*) and is similar to blank levels of other materials at the USGS Radiocarbon Laboratory including Alfa Aesar graphite (0.0020 ± 0.0011) and Carrera marble (0.0018 ± 0.0014).

#### Calibration

All ^14^C ages were calibrated using OxCal v.4.4.4 (*57*) and the IntCal20 dataset (*58*). Ages are presented in thousands of calibrated years before present (0 ka = 1950 CE), and uncertainties are given at the 95% (2σ) confidence level.

### Geochemical analysis

Mineralogical characterization of bulk sediment samples was conducted using x-ray diffractometry (XRD) at the USGS in Denver, CO. Sediment samples were first oven-dried, then manually crushed, and homogenized using a mortar and pestle. To ensure uniform particle size, the samples were sieved in accordance with standard XRD sample preparation protocols. The resulting powders were mounted in back-loaded sample holders to ensure a randomly oriented surface, minimizing preferred orientation effects. XRD analyses were performed using a Malvern Panalytical Aeris diffractometer (Malvern Panalytical, The Netherlands), equipped with a θ/θ goniometer and a copper (Cu) anode producing Cu Kα radiation (40 kV, 15 mA). Diffraction data were collected over a 2θ range of 5.0064° to 84.9874°, with a step size of 0.0110°, suitable for identifying common sedimentary minerals. Phase identification was performed using Malvern Panalytical’s HighScore software, in conjunction with the PDF-4 Minerals database from the International Centre for Diffraction Data. The XRD results are presented in figure S22.

### Biomarker analysis

Sediments from each of the units depicted in the composite stratigraphic section (fig. S21) were freeze dried and homogenized. Approximately 50 g of sediment for each sample was extracted for lipids using a Thermo Dionex Accelerated Solvent Extractor 350 and dichloromethane (DCM):methanol (9:1, v/v). The resulting total lipid extracts were separated into four fractions over a dual-layer column with LC-NH_2_ gel in the lower layer and 5% deactivated silica in the upper-layer. The fractions collected were aliphatics (F1, eluted with 100% hexane), ketones (F2, eluted with 100% DCM), GDGTs (F3, eluted with DCM:isopropanol) (2:1, v/v), and fatty acids (F4, eluted with 5% acetic acid in DCM).

The GDGT fraction was dried with N_2_ gas, redissolved in hexane:isopropanol (99:1, v/v), and filtered through a 0.45-μm PTFE filter before analysis. Samples were analyzed on an Agilent 1260 high-performance liquid chromatograph coupled to an Agilent 6120 quadrupole mass spectrometer using the method of (*59*) and single ion monitoring mode. Both isoprenoid and branched GDGTs were analyzed and quantified relative to a synthetic C_46_ internal standard (*60*).

The fatty acid fraction was methylated overnight at 50°C in a solution of 5% acetyl chloride in methanol. The resulting fatty acid methyl esters (FAMEs) were purified over a 5% deactivated silica gel column, eluting with DCM. FAMEs were quantified using a Thermo Trace 1310 gas chromatograph (GC) connected to a flame ionization detector and then analyzed for their δ^2^H composition using a Trace 1310 GC coupled to a Thermo Delta V Plus isotope ratio mass spectrometer. Samples were run in at least triplicate to reduce SE. Machine drift and isotopic accuracy was monitored with repeat injections of the A7 *n*-alkane mix (from Arndt Schimmelmann, Indiana University) throughout analysis. Mass balance corrections were made for the methyl group added during methylation; the δ^2^H composition of the methanol used for the reaction was determined by analysis of methylated phthalic acid of a known composition (*61*).

We focused on analyzing δ^2^H of the C_28_ and C_30_ fatty acids, the most abundant homologs in the sediments produced by higher plants. The δ^2^H composition of these two homologs is very similar, so we used their average to represent δ^2^H_wax_.

### Pollen analysis

To characterize contemporary local vegetation, representative samples from the stratigraphic sequence at WHSA Locality 2 were processed for quantitative pollen counts in the USGS Radiocarbon Laboratory as follows. Around 10 g of sample (air dried) was placed in 250-ml Nalgene beaker along with two lycopodium tablets (batch no. 20890,^−^X = 11,267), covered with 1 M HCl, and allowed to fully react. Samples were washed with deionized (DI) water before being sieved using a 150-μm stainless 3-inch (7.62-cm) mesh screen into a 250-ml beaker and then transferred back into the 50-ml tubes using a swirl/settle technique, stranding heavier particles in the beaker (the heavy fraction was optically verified to contain no pollen). Samples were disaggregated with a vortex mixer, and 15 to 20 ml of 6 M HCl were mixed in, covered lightly, and allowed to react overnight. Samples were centrifuged and decanted and revortexed, ∼20 ml of 6 M HCl were added, and then tubes were placed in a heating block set to 80°C for more than 2 hours before being centrifuged and decanted. The samples were disaggregated with the vortex and slowly mixed with ∼5 ml of concentrated HF and allowed to react for up to 1 hour. Samples were then vortexed and mixed with fresh HF (∼10 ml) and allowed to sit, lightly covered overnight, then centrifuged, and decanted. Fresh HF (∼10 ml) was again added, and the tubes were placed in the heating block for 2 hours or more, centrifuged, and decanted. Following this, the samples were vortexed; ∼10 ml of 6 M HCl were added; and samples were placed back in the heating block at 80°C for ∼2 hours, then centrifuged, and decanted. The samples were washed/vortexed with DI water for a minimum of three times and then washed into 15-ml Pyrex tubes, where the volume was noted.

At this stage, slides were made to assess mineral content and note the condition of organic content. Each sample was then treated with a Schulze solution (∼2 ml of water, saturated with KClO_3_, and ∼2 ml of concentrated HNO_3_), mixed, and placed in the heating bloc for 6 min at 80°C. DI water was added to stop the reaction; tubes were then centrifuged and decanted. Each sample was then stirred and washed with DI three times. Roughly 3 ml of 10% KOH was added to the samples, agitated with the vortexer, and placed in the heating block for 10 min at 80°C, then filled with DI water, and centrifuged and decanted a minimum of three times.

Final slides were then made by hand using a glass pipette, 1 ^1^/_2_ drops of glycerin gel, and a toothpick lightly dipped into Safranin O stain, stirred, and then covered with a cover slip. The slides were then suspended upside down in a slide warmer (on thick toothpicks) to allow the grains to settle on the underside of the cover slip. They were then transferred onto the countertop, still upside down to allow the gel to set up.

Counting and identification of palynomorphs was carried out with a transmitted light microscope at ×400 magnification at Universidad de Granada. Pollen grains are relatively abundant in the samples, and between 200 and 525 terrestrial pollen grains were classified for each sample (table S13). Pollen reference materials of plants that occur in the American Southwest from the palynological laboratory at Northern Arizona University were used for certain identifications. *Pinus* pollen was divided into three categories: *Pinus diploxylon* (*Pinus ponderosa* or *Pinus contorta*), *Pinus haploxylon* (*Pinus aristata* or *Pinus flexilis*) and *Pinus edulis* (piñon). *P. edulis* pollen was differentiated from the rest of *Pinus* indeterminate pollen by its small size (generally less than 65 μm in diameter) and the presence of verrucae on the leptoma (*62*, *63*).

### Principal components analysis

To isolate shared modes of variability between the biomarker and pollen data, we performed a PCA on the combined dataset. For the pollen we used a reduced dataset consisting only of the major representative taxa: *Pinus* (piñon treated separately), Chenopodiaceae/Amaranthaceae (ChenoAm), *Juniperus*, *Quercus*, Poaceae, Asteraceae (*Artemisia* and *Ambrosia* treated separately), *Abies*/*Picea*/*Pseudotsuga* (Spruce and Fir combined), and *Ruppia*. The biomarkers were analyzed at a higher resolution than the pollen, so the PCA only included data points where both types of data were present (*n* = 20; table S14). Results indicated that 59% of the variance in the dataset can be explained by the two leading modes (39 and 20%). We then applied varimax rotation to these two modes to minimize cross-correlation and improve interpretability (fig. S25). As a result, variance is cleanly separated into two modes that represent two main types of changes in the dataset.

The first mode (PC1) consists of strong positive loading from GDGT-based temperature, soil GDGTs, Oak (*Quercus*), and ChenoAm, all of which increase through time in the WHSA Locality 2 record (fig. S25). Conversely there is strong negative loading from *Pinus*, juniper (*Juniperus*), Lake GDGTs, and Spruce/Fir, which decrease through time in the record. The pollen species involved in this mode are all temperature sensitive, and the GDGTs involved represent temperature and the water depth of the Tularosa Basin. Therefore, PC1 describes long-term increase in temperature across the WHSA Locality 2 record as well as the overall change in moisture that occurs as higher temperatures increase evaporation. Cold, wet, and deeper lake conditions at 24 ka transitioned to warm, dry, and playa conditions by 22 ka, with accelerated changes toward the end (Fig. 4A).

The second mode (PC2) contains strong positive loadings from δ^2^H_p_, salty GDGTs, IR67, ACE, and piñon (fig. S25). The strongest negative loading comes from *Ruppia*. Temporally, this mode shows centennial-scale oscillations with maximum values during D-O 2, when all of the GDGT indicators indicate salty conditions in the basin, δ^2^H_p_ is enriched, and *Ruppia* is absent (Fig. 4B). This mode can, therefore, be interpreted as short-term fluctuations in aridity, when reduced rainfall resulted in drying and salinification of the playa. *Ruppia* percentages rise when PC2 is lower, i.e., when the playa is wet and can support this emergent aquatic plant.

### Ostracode analysis

Processing bulk sediment samples to extract fossil ostracode shells was carried out following standard procedures involving washing, freezing, and freeze drying (*64*). All samples were weighed and individually placed in beakers to which 800 ml of warm, distilled water and 3 g of baking soda were added. The disaggregated samples were then gently washed through a stack of standard 8-inch (20.32-cm)–diameter brass sieves of 850-, 150-, and 63-μm mesh. Most of the ostracodes were collected on the 150-μm mesh sieve. The washed subsamples were then decanted into Whirlpak bags, frozen, and then freeze-dried. Freeze-dried samples were then examined under a Leica-MZ 12.5 stereomicroscope.

Species identifications, ecological tolerances, and biogeographic ranges follow Smith and Delorme (*65*), Smith and Horne (*66*), the North American Nonmarine Ostracode database (*67*), and the Neotoma database (*68*), as well as relevant taxonomic publications cited within these sources. Species counts were recorded, and the freeze-dried samples were stored in labeled slides and vials. The samples are currently housed in the Paleolimnology Lab, Earth Sciences Department of Kent State University.

## Supplementary Materials

### This PDF file includes:

Supplementary Text Figs. S1 to S27 Tables S1 to S15 References
