# Supplementary material for: Humans experienced abrupt environmental changes at White Sands during the Last Glacial Maximum
Source: Sci Adv. 2026 Oct 14;12(42):eaeh0707. doi: 10.1126/sciadv.aeh0707 (PMC13666055; doi:10.1126/sciadv.aeh0707)
Supplement: Supplementary file 1 [file sciadv.aeh0707_sm.pdf]

Science Advances
AAAS

Supplementary Materials for

Humans experienced abrupt environmental changes at White Sands during the Last Glacial Maximum

Kathleen B. Springer *et al.*

Corresponding author: Kathleen B. Springer, kspringer@usgs.gov; Jeffrey S. Pigati, jpigati@usgs.gov

Sci. Adv. **12**, eaeh0707 (2026)
DOI: 10.1126/sciadv.aeh0707

This PDF file includes:

Supplementary Text
Figs. S1 to S27
Tables S1 to S15
References

Supplementary text

1. Radiocarbon dating

1.1 Data evaluation

We considered four different age model hypotheses when evaluating the chronologic data from WHSA Locality 2: (1) *Ruppia* seed ages only, uncorrected, (2) *Ruppia* seed ages and pollen ages, both uncorrected, (3) *Ruppia* seed ages only, corrected assuming a constant hard-water effect, and (4) *Ruppia* seed ages only, corrected assuming a variable hard-water effect. The first two age model hypotheses did not require corrections of the measured ^{14}C data (Table S4).

To determine the average hard-water effect (HWE) value for age model hypothesis 3, we used paired pollen-*Ruppia* ages for samples 22WHSA1-18.1n/nn and 22WHSA1-18.1r/rr as well as a paired pollen age and modeled *Ruppia* seed age for sample 22WHSA1-18.1hh. We first converted each of the ^{14}C ages to ^{14}C activity values, determined the difference between each pair while adding the uncertainties quadratically, and calculated the average HWE value, which was 0.0107±0.0044 (Table S5). We then added this average value to the measured ^{14}C activity of every *Ruppia* seed sample and calculated a suite of HWE-corrected ^{14}C ages. The temporal magnitude of the correction ranged from 0.72 to 1.05 ^{14}C kyrs (Table S6).

To determine hard-water effects for age model hypothesis 4, we used paired pollen-*Ruppia* seed ages for samples 22WHSA1-18.1n/nn and 22WHSA1-18.1r/rr, and paired *Ruppia* seed ages and interpolated pollen ages for samples 22WHSA1-18.1k and 22WHSA1-18.1q. As above, we began by converting the ^{14}C ages to ^{14}C activity values and then determined the difference between each pair while adding the uncertainties quadratically (Table S7). We then added the HWE value to the ^{14}C activity of the corresponding *Ruppia* seed activity before calculating a HWE-corrected ^{14}C age for each of these four samples. All other *Ruppia* seed ages remained unchanged. The temporal magnitude of the correction ranged from 0.75 to 1.39 ^{14}C kyrs (Table S8).

1.2. Numerical modeling

For all four age model hypotheses, we determined ages for the sediments at WHSA Locality 2 using the `Deposition`, `Sequence`, and `Boundaries` functions in OxCal v.4.4.4 (*57*) and the IntCal20 calibration curve (*58*); accessed online 20 July 2025. Samples USGS-1803 and USGS-1804 were identified as outliers at the 95% confidence level, so were excluded from all of the analyses (Table S4).

For input, we used the uncorrected ^{14}C ages for age model hypotheses 1 and 2 (n=20 and 26, respectively; Table S4) and the corrected ^{14}C ages for age model hypotheses 3 and 4 (n=20 for both; Tables S6 and S8) for the numerical modeling exercises, along with the elevation of each sample, the elevation of each track horizon, and the average elevation of each track package. Notably, we did not use the new pollen ^{14}C ages (USGS-2330, 2331, 2332) as input for age model hypotheses 3 and 4 because they were used to determine the HWE values and correct the ^{14}C ages of the *Ruppia* seeds, and are therefore not independent of the *Ruppia* ages. Moreover, we did not include the previously published pollen ^{14}C ages (CAMS-190357A, 190358A, 190359A) in the modeling exercises because the uncertainties were so large that they did not impact the final results.

The codes used for the deposition models (excluding the two outlier ages) are as follows:

Age Model Hypothesis 1 – *Ruppia* seed ages only, uncorrected

```
Plot()
 {
  P_Sequence("WHSA Extended Locality 2",1,5e+2,U(-2,2))
  {
   Boundary("Start");
   Date("Bottom"){z=1195.482675;};
   R_Date("USGS-2076",20640,110){z=1196.256325;};
   Date("THminus-3"){z=1196.258575;};
   R_Date("USGS-2075",20490,110){z=1196.336350;};
   Date("THminus-2"){z=1196.3386000;};
   R_Date("USGS-2074",20160,100){z=1196.518600;};
   Date("THminus-1"){z=1196.521775;};
   Date("TH0"){z=1196.601775;};
   R_Date("USGS-2073",19570,100){z=1196.738450;};
   Date("TH1"){z=1197.081425;};
   Date("TH2"){z=1197.137550;};
   R_Combine("USGS-1808.1/1808/1830/1810"){R_Date("USGS-1808.1",18950,110);
   R_Date("USGS-1808",19020,110);R_Date("USGS-
      1830",18810,100);R_Date("USGS-1810",19010,120);z=1197.143725;};
   Date("TH3"){z=1197.185740;};
   Date("TH4"){z=1197.217525;};
   R_Date("USGS-1807",18680,110){z=1197.267475;};
   Date("TH5"){z=1197.267525;};
   R_Combine("USGS-1805/1829/2071"){R_Date("USGS-
      1805",18550,100);R_Date("USGS-1829",18660,100);R_Date("USGS-
      2071",18640,90);z=1197.277475;};
   R_Date("USGS-2070",18760,90){z=1197.300025;};
   R_Combine("USGS-1828/1806/2069"){R_Date("USGS-
      1828",17930,90);R_Date("USGS-1806",18200,110);R_Date("USGS-
      2069",18860,90);z=1197.356025;};
   Date("TH6"){z=1197.358575;};
   Date("TH7"){z=1197.387360;};
   R_Date("USGS-2068",18650,90){z=1197.459225;};
   Date("TP8"){z=1197.488950;};
   Date("TP9"){z=1197.598950;};
   R_Date("USGS-2067",18410,90){z=1197.637000;};
   Date("TP10"){z=1197.713950;};
   R_Date("USGS-2066",18190,80){z=1197.716425;};
   R_Date("USGS-2065",17760,80){z=1197.759225;};
   Date("TP11"){z=1197.793950;};
   Date("TP12"){z=1197.853950;};
   Date("TP13"){z=1197.903950;};
   Date("TP14"){z=1197.938950;};
   Date("TP15"){z=1197.983950;};
   Date("TP16"){z=1198.078950;};
   Date("Top"){z=1198.148950;};
  Boundary("End");
  };
 };
```

Age Model Hypothesis 2 – *Ruppia* seed ages and pollen ages, both uncorrected

```
Plot()
 {
  P_Sequence("WHSA Extended Locality 2",1,5e+2,U(-2,2))
  {
   Boundary("Start");
```

```
    Date("Bottom"){z=1195.482675;};
    R_Combine("USGS-2076/2332"){R_Date("USGS-2076",20640,110);R_Date("USGS-
        2332",19520,200);z=1196.256325;};
    Date("THminus-3"){z=1196.258575;};
    R_Date("USGS-2075",20490,110){z=1196.336350;};
    Date("THminus-2"){z=1196.3386000;};
    R_Combine("USGS-2074/2331"){R_Date("USGS-2074",20160,100);R_Date("USGS-
       2331",18830,180);z=1196.518600;};
    Date("THminus-1"){z=1196.521775;};
    Date("TH0"){z=1196.601775;};
    R_Date("USGS-2073",19570,100){z=1196.738450;};
    R_Date("USGS-2330",18820,180){z=1196.861795;};
    Date("TH1"){z=1197.081425;};
    Date("TH2"){z=1197.137550;};
    R_Combine("USGS-1808.1/1808/1830/1810"){R_Date("USGS-1808.1",18950,110);
    R_Date("USGS-1808",19020,110);R_Date("USGS-
       1830",18810,100);R_Date("USGS-1810",19010,120);z=1197.143725;};
    Date("TH3"){z=1197.185740;};
    Date("TH4"){z=1197.217525;};
    R_Combine("USGS-1807 and CAMS-190359A"){R_Date("USGS-
       1807",18680,110);R_Date("CAMS-190359A",18500,950);z=1197.267475;};
    Date("TH5"){z=1197.267525;};
    R_Combine("USGS-1805/1829/2071 and CAMS-190358A"){R_Date("USGS-
       1805",18550,100);R_Date("USGS-1829",18660,100);R_Date("USGS-
       2071",18640,90);R_Date("CAMS-190358A",18600,960);z=1197.277475;};
    R_Date("USGS-2070",18760,90){z=1197.300025;};
    R_Combine("USGS-1828/1806/2069 and CAMS-190357A"){R_Date("USGS-
       1828",17930,90);R_Date("USGS-1806",18200,110);R_Date("USGS-
       2069",18860,90);R_Date("CAMS-190357A",19250,1050);z=1197.356025;};
    Date("TH6"){z=1197.358575;};
    Date("TH7"){z=1197.387360;};
    R_Date("USGS-2068",18650,90){z=1197.459225;};
    Date("TP8"){z=1197.488950;};
    Date("TP9"){z=1197.598950;};
    R_Date("USGS-2067",18410,90){z=1197.637000;};
    Date("TP10"){z=1197.713950;};
    R_Date("USGS-2066",18190,80){z=1197.716425;};
    R_Date("USGS-2065",17760,80){z=1197.759225;};
    Date("TP11"){z=1197.793950;};
    Date("TP12"){z=1197.853950;};
    Date("TP13"){z=1197.903950;};
    Date("TP14"){z=1197.938950;};
    Date("TP15"){z=1197.983950;};
    Date("TP16"){z=1198.078950;};
    Date("Top"){z=1198.148950;};
   Boundary("End");
   };
  };
```

Age Model Hypothesis 3 – *Ruppia* seed ages only, corrected assuming a constant hard-water effect

```
Plot()
 {
  P_Sequence("WHSA Extended Locality 2",1,5e+2,U(-2,2))
  {
    Boundary("Start");
    Date("Bottom"){z=1195.482675;};
    R_Date("USGS-2076",19590,430){z=1196.256325;};
    Date("THminus-3"){z=1196.258575;};
```

```
   R_Date("USGS-2075",19460,420){z=1196.336350;};
   Date("THminus-2"){z=1196.3386000;};
   R_Date("USGS-2074",19170,400){z=1196.518600;};
   Date("THminus-1"){z=1196.521775;};
   Date("TH0"){z=1196.601775;};
   R_Date("USGS-2073",18640,380){z=1196.738450;};
   Date("TH1"){z=1197.081425;};
   Date("TH2"){z=1197.137550;};
   R_Combine("USGS-1808.1/1808/1830/1810"){R_Date("USGS-
      1808.1",18090,360);R_Date("USGS-1808",18150,360);R_Date("USGS-
      1830",17960,350);R_Date("USGS-1810",18150,360);z=1197.143725;};
   Date("TH3"){z=1197.185740;};
   Date("TH4"){z=1197.217525;};
   R_Date("USGS-1807",17850,350){z=1197.267475;};
   Date("TH5"){z=1197.267525;};
   R_Combine("USGS-1805/1829/2071"){R_Date("USGS-
      1805",17730,340);R_Date("USGS-1829",17830,340);R_Date("USGS-
      2071",17810,340);z=1197.277475;};
   R_Date("USGS-2070",17920,350){z=1197.300025;};
   R_Combine("USGS-1828/1806/2069"){R_Date("USGS-
      1828",17170,320);R_Date("USGS-1806",17410,330);R_Date("USGS-
      2069",18010,350);z=1197.356025;};
   Date("TH6"){z=1197.358575;};
   Date("TH7"){z=1197.387360;};
   R_Date("USGS-2068",17820,340){z=1197.459225;};
   Date("TP8"){z=1197.488950;};
   Date("TP9"){z=1197.598950;};
   R_Date("USGS-2067",17600,330){z=1197.637000;};
   Date("TP10"){z=1197.713950;};
   R_Date("USGS-2066",17400,320){z=1197.716425;};
   R_Date("USGS-2065",17010,310){z=1197.759225;};
   Date("TP11"){z=1197.793950;};
   Date("TP12"){z=1197.853950;};
   Date("TP13"){z=1197.903950;};
   Date("TP14"){z=1197.938950;};
   Date("TP15"){z=1197.983950;};
   Date("TP16"){z=1198.078950;};
   Date("Top"){z=1198.148950;};
  Boundary("End");
  };
 };
```

Age Model Hypothesis 4 – *Ruppia* seed ages only, corrected assuming a variable hard-water effect

```
Plot()
 {
  P_Sequence("WHSA Extended Locality 2",1,5e+2,U(-2,2))
  {
   Boundary("Start");
   Date("Bottom"){z=1195.482675;};
   R_Date("USGS-2076",19620,280){z=1196.256325;};
   Date("THminus-3"){z=1196.258575;};
   R_Date("USGS-2075",19100,260){z=1196.336350;};
   Date("THminus-2"){z=1196.3386000;};
   R_Date("USGS-2074",19240,240){z=1196.518600;};
   Date("THminus-1"){z=1196.521775;};
   Date("TH0"){z=1196.601775;};
   R_Date("USGS-2073",18820,220){z=1196.738450;};
   Date("TH1"){z=1197.081425;};
```

```
   Date("TH2"){z=1197.137550;};
   R_Combine("USGS-1808.1/1808/1830/1810"){R_Date("USGS-1808.1",18950,110);
   R_Date("USGS-1808",19020,110);R_Date("USGS-
      1830",18810,100);R_Date("USGS-1810",19010,120);z=1197.143725;};
   Date("TH3"){z=1197.185740;};
   Date("TH4"){z=1197.217525;};
   R_Date("USGS-1807",18680,110){z=1197.267475;};
   Date("TH5"){z=1197.267525;};
   R_Combine("USGS-1805/1829/2071"){R_Date("USGS-
      1805",18550,100);R_Date("USGS-1829",18660,100);R_Date("USGS-
      2071",18640,90);z=1197.277475;};
   R_Date("USGS-2070",18760,90){z=1197.300025;};
   R_Combine("USGS-1828/1806/2069"){R_Date("USGS-
      1828",17930,90);R_Date("USGS-1806",18200,110);R_Date("USGS-
      2069",18860,90);z=1197.356025;};
   Date("TH6"){z=1197.358575;};
   Date("TH7"){z=1197.387360;};
   R_Date("USGS-2068",18650,90){z=1197.459225;};
   Date("TP8"){z=1197.488950;};
   Date("TP9"){z=1197.598950;};
   R_Date("USGS-2067",18410,90){z=1197.637000;};
   Date("TP10"){z=1197.713950;};
   R_Date("USGS-2066",18190,80){z=1197.716425;};
   R_Date("USGS-2065",17760,80){z=1197.759225;};
   Date("TP11"){z=1197.793950;};
   Date("TP12"){z=1197.853950;};
   Date("TP13"){z=1197.903950;};
   Date("TP14"){z=1197.938950;};
   Date("TP15"){z=1197.983950;};
   Date("TP16"){z=1198.078950;};
   Date("Top"){z=1198.148950;};
  Boundary("End");
  };
 };
```

The codes used for the sequence models (excluding the two outlier ages) are as follows:

<u>Age Model Hypothesis 1 – *Ruppia* seed ages only, uncorrected</u>

```
Plot()
{
Outlier_Model("General",T(5),U(0,4),"t");
Sequence()
  {
      Boundary("Start Extended WHSA Locality 2");
        Sequence("WHSA Extended Locality 2")
            {
              Phase("1196.26 masl")
                  {
                    R_Date("USGS-2076",20640,110)
                        {Outlier("General",0.05); };
                    Date("THminus3",Track Horizon minus 3);
                  };
              Phase("1196.34 masl")
                  {
                    R_Date("USGS-2075",20490,110)
                         {Outlier("General",0.05); };
                    Date("THminus2",Track Horizon minus 2);
                  };
              Phase("1196.52 masl")
```

```
    {
      R_Date("USGS-2074",20160,100)
          {Outlier("General",0.05); };
      Date("THminus1",Track Horizon minus 1);
    };
Date("TH0",Track Horizon 0);
R_Date("USGS-2073",19570,100)
     {Outlier("General",0.05); };
Date("TH1",Track Horizon 1);
Phase("1197.14 masl")
    {
      R_Date("USGS-1810",19010,120)
          {Outlier("General",0.05); };
      R_Date("USGS-1830",18810,100)
          {Outlier("General",0.05); };
      R_Date("USGS-1808",19020,110)
          {Outlier("General",0.05); };
      R_Date("USGS-1808.1",18950,110)
          {Outlier("General",0.05); };
      Date("TH2",Track Horizon 2);
    };
Date("TH3",Track Horizon 3);
Date("TH4",Track Horizon 4);
Phase("1197.27 masl")
    {
      R_Date("USGS-1807",18680,110)
          {Outlier("General",0.05); };
      Date("TH5",Track Horizon 5);
    };
Phase("1197.28 masl")
    {
      R_Date("USGS-2071",18640,90)
          {Outlier("General",0.05); };
      R_Date("USGS-1829",18660,100)
          {Outlier("General",0.05); };
      R_Date("USGS-1805",18550,100)
          {Outlier("General",0.05); };
    };
Phase("1197.30 masl")
    {
      R_Date("USGS-2070",18760,90)
          {Outlier("General",0.05); };
    };
Phase("1197.36 masl")
    {
      R_Date("USGS-2069",18860,90)
          {Outlier("General",0.05); };
      R_Date("USGS-1806",18200,110)
          {Outlier("General",0.05); };
      R_Date("USGS-1828",17930,90)
          {Outlier("General",0.05); };
      Date("TH6",Track Horizon 6);
    };
Date("TH7",Track Horizon 7);
R_Date("USGS-2068",18650,90)
     {Outlier("General",0.05); };
Date("TP8",Track Package 8);
Date("TP9",Track Package 9);
R_Date("USGS-2067",18410,90)
     {Outlier("General",0.05); };
Date("TP10",Track Package 10);
```

```
            R_Date("USGS-2066",18190,80)
                 {Outlier("General",0.05); };
            R_Date("USGS-2065",17760,80)
                 {Outlier("General",0.05); };
            Date("TP11",Track Package 11);
            Date("TP12",Track Package 12);
            Date("TP13",Track Package 13);
            Date("TP14",Track Package 14);
            Date("TP15",Track Package 15);
            Date("TP16",Track Package 16);
          };
      Boundary("End Extended WHSA Locality 2");
  };
};
```

Age Model Hypothesis 2 – *Ruppia* seed ages and pollen ages, both uncorrected

```
Plot()
{
Outlier_Model("General",T(5),U(0,4),"t");
Sequence()
  {
      Boundary("Start Extended WHSA Locality 2");
        Sequence("WHSA Extended Locality 2")
          {
            Phase("1196.26 masl")
                {
                  R_Date("USGS-2076",20640,110)
                      {Outlier("General",0.05); };
                  R_Date("USGS-2332",19520,200)
                      {Outlier("General",0.05); };
                  Date("THminus3",Track Horizon minus 3);
                };
            Phase("1196.34 masl")
                {
                  R_Date("USGS-2075",20490,110)
                       {Outlier("General",0.05); };
                  Date("THminus2",Track Horizon minus 2);
                };
            Phase("1196.52 masl")
                {
                  R_Date("USGS-2074",20160,100)
                      {Outlier("General",0.05); };
                  R_Date("USGS-2331",18830,180)
                      {Outlier("General",0.05); };
                  Date("THminus1",Track Horizon minus 1);
                };
            Date("TH0",Track Horizon 0);
            R_Date("USGS-2073",19570,100)
                 {Outlier("General",0.05); };
            R_Date("USGS-2330",18820,180)
                 {Outlier("General",0.05); };
            Date("TH1",Track Horizon 1);
            Phase("1197.14 masl")
                {
                  R_Date("USGS-1810",19010,120)
                      {Outlier("General",0.05); };
                  R_Date("USGS-1830",18810,100)
                      {Outlier("General",0.05); };
                  R_Date("USGS-1808",19020,110)
```

```
          {Outlier("General",0.05); };
      R_Date("USGS-1808.1",18950,110)
          {Outlier("General",0.05); };
      Date("TH2",Track Horizon 2);
    };
Date("TH3",Track Horizon 3);
Date("TH4",Track Horizon 4);
Phase("1197.27 masl")
    {
      R_Date("CAMS-190359A",18500,950)
          {Outlier("General",0.05); };
      R_Date("USGS-1807",18680,110)
          {Outlier("General",0.05); };
      Date("TH5",Track Horizon 5);
    };
Phase("1197.28 masl")
    {
      R_Date("CAMS-190358A",18600,960)
          {Outlier("General",0.05); };
      R_Date("USGS-2071",18640,90)
          {Outlier("General",0.05); };
      R_Date("USGS-1829",18660,100)
          {Outlier("General",0.05); };
      R_Date("USGS-1805",18550,100)
          {Outlier("General",0.05); };
    };
Phase("1197.30 masl")
    {
      R_Date("USGS-2070",18760,90)
          {Outlier("General",0.05); };
    };
Phase("1197.36 masl")
    {
      R_Date("CAMS-190357A",19250,1050)
          {Outlier("General",0.05); };
      R_Date("USGS-2069",18860,90)
          {Outlier("General",0.05); };
      R_Date("USGS-1806",18200,110)
          {Outlier("General",0.05); };
      R_Date("USGS-1828",17930,90)
          {Outlier("General",0.05); };
      Date("TH6",Track Horizon 6);
    };
Date("TH7",Track Horizon 7);
R_Date("USGS-2068",18650,90)
     {Outlier("General",0.05); };
Date("TP8",Track Package 8);
Date("TP9",Track Package 9);
R_Date("USGS-2067",18410,90)
     {Outlier("General",0.05); };
Date("TP10",Track Package 10);
R_Date("USGS-2066",18190,80)
     {Outlier("General",0.05); };
R_Date("USGS-2065",17760,80)
     {Outlier("General",0.05); };
Date("TP11",Track Package 11);
Date("TP12",Track Package 12);
Date("TP13",Track Package 13);
Date("TP14",Track Package 14);
Date("TP15",Track Package 15);
Date("TP16",Track Package 16);
```

```
            };
      Boundary("End Extended WHSA Locality 2");
  };
};
```

Age Model Hypothesis 3 – *Ruppia* seed ages only, corrected assuming a constant hard-water effect

```
Plot()
{
Outlier_Model("General",T(5),U(0,4),"t");
Sequence()
  {
      Boundary("Start Extended WHSA Locality 2");
        Sequence("WHSA Extended Locality 2")
            {
              Phase("1196.26 masl")
                  {
                    R_Date("USGS-2076",19590,430)
                        {Outlier("General",0.05); };
                    Date("THminus3",Track Horizon minus 3);
                  };
              Phase("1196.34 masl")
                  {
                    R_Date("USGS-2075",19460,420)
                         {Outlier("General",0.05); };
                    Date("THminus2",Track Horizon minus 2);
                  };
              Phase("1196.52 masl")
                  {
                    R_Date("USGS-2074",19170,400)
                        {Outlier("General",0.05); };
                    Date("THminus1",Track Horizon minus 1);
                  };
              Date("TH0",Track Horizon 0);
              R_Date("USGS-2073",18640,380)
                   {Outlier("General",0.05); };
              Date("TH1",Track Horizon 1);
              Phase("1197.14 masl")
                  {
                    R_Date("USGS-1810",18140,360)
                        {Outlier("General",0.05); };
                    R_Date("USGS-1830",17960,350)
                        {Outlier("General",0.05); };
                    R_Date("USGS-1808",18150,360)
                        {Outlier("General",0.05); };
                    R_Date("USGS-1808.1",18090,360)
                        {Outlier("General",0.05); };
                    Date("TH2",Track Horizon 2);
                  };
              Date("TH3",Track Horizon 3);
              Date("TH4",Track Horizon 4);
              Phase("1197.27 masl")
                  {
                    R_Date("USGS-1807",17850,350)
                        {Outlier("General",0.05); };
                    Date("TH5",Track Horizon 5);
                  };
              Phase("1197.28 masl")
                  {
```

```
                    R_Date("USGS-2071",17810,340)
                        {Outlier("General",0.05); };
                    R_Date("USGS-1829",17830,340)
                        {Outlier("General",0.05); };
                    R_Date("USGS-1805",17730,340)
                        {Outlier("General",0.05); };
                  };
              Phase("1197.30 masl")
                  {
                    R_Date("USGS-2070",17920,350)
                        {Outlier("General",0.05); };
                  };
              Phase("1197.36 masl")
                  {
                    R_Date("USGS-2069",18010,350)
                        {Outlier("General",0.05); };
                    R_Date("USGS-1806",17410,330)
                        {Outlier("General",0.05); };
                    R_Date("USGS-1828",17170,320)
                        {Outlier("General",0.05); };
                    Date("TH6",Track Horizon 6);
                  };
              Date("TH7",Track Horizon 7);
              R_Date("USGS-2068",17820,340)
                   {Outlier("General",0.05); };
              Date("TP8",Track Package 8);
              Date("TP9",Track Package 9);
              R_Date("USGS-2067",17600,330)
                   {Outlier("General",0.05); };
              Date("TP10",Track Package 10);
              R_Date("USGS-2066",17400,320)
                   {Outlier("General",0.05); };
              R_Date("USGS-2065",17010,310)
                   {Outlier("General",0.05); };
              Date("TP11",Track Package 11);
              Date("TP12",Track Package 12);
              Date("TP13",Track Package 13);
              Date("TP14",Track Package 14);
              Date("TP15",Track Package 15);
              Date("TP16",Track Package 16);
            };
      Boundary("End Extended WHSA Locality 2");
  };
};
```

Age Model Hypothesis 4 – *Ruppia* seed ages only, corrected assuming a variable hard-water effect

```
Plot()
{
Outlier_Model("General",T(5),U(0,4),"t");
Sequence()
  {
      Boundary("Start Extended WHSA Locality 2");
        Sequence("WHSA Extended Locality 2")
            {
              Phase("1196.26 masl")
                  {
                    R_Date("USGS-2076",19620,280)
                        {Outlier("General",0.05); };
```

```
      Date("THminus3",Track Horizon minus 3);
     };
Phase("1196.34 masl")
     {
      R_Date("USGS-2075",19100,260)
           {Outlier("General",0.05); };
      Date("THminus2",Track Horizon minus 2);
     };
Phase("1196.52 masl")
     {
      R_Date("USGS-2074",19240,240)
          {Outlier("General",0.05); };
      Date("THminus1",Track Horizon minus 1);
     };
Date("TH0",Track Horizon 0);
R_Date("USGS-2073",18820,220)
     {Outlier("General",0.05); };
Date("TH1",Track Horizon 1);
Phase("1197.14 masl")
     {
      R_Date("USGS-1810",19010,120)
          {Outlier("General",0.05); };
      R_Date("USGS-1830",18810,100)
          {Outlier("General",0.05); };
      R_Date("USGS-1808",19020,110)
          {Outlier("General",0.05); };
      R_Date("USGS-1808.1",18950,110)
          {Outlier("General",0.05); };
      Date("TH2",Track Horizon 2);
     };
Date("TH3",Track Horizon 3);
Date("TH4",Track Horizon 4);
Phase("1197.27 masl")
     {
      R_Date("USGS-1807",18680,110)
          {Outlier("General",0.05); };
      Date("TH5",Track Horizon 5);
     };
Phase("1197.28 masl")
     {
      R_Date("USGS-2071",18640,90)
          {Outlier("General",0.05); };
      R_Date("USGS-1829",18660,100)
          {Outlier("General",0.05); };
      R_Date("USGS-1805",18550,100)
          {Outlier("General",0.05); };
     };
Phase("1197.30 masl")
     {
      R_Date("USGS-2070",18760,90)
          {Outlier("General",0.05); };
     };
Phase("1197.36 masl")
     {
      R_Date("USGS-2069",18860,90)
          {Outlier("General",0.05); };
      R_Date("USGS-1806",18200,110)
          {Outlier("General",0.05); };
      R_Date("USGS-1828",17930,90)
          {Outlier("General",0.05); };
      Date("TH6",Track Horizon 6);
```

```
                    };
                Date("TH7",Track Horizon 7);
                R_Date("USGS-2068",18650,90)
                     {Outlier("General",0.05); };
                Date("TP8",Track Package 8);
                Date("TP9",Track Package 9);
                R_Date("USGS-2067",18410,90)
                     {Outlier("General",0.05); };
                Date("TP10",Track Package 10);
                R_Date("USGS-2066",18190,80)
                     {Outlier("General",0.05); };
                R_Date("USGS-2065",17760,80)
                     {Outlier("General",0.05); };
                Date("TP11",Track Package 11);
                Date("TP12",Track Package 12);
                Date("TP13",Track Package 13);
                Date("TP14",Track Package 14);
                Date("TP15",Track Package 15);
                Date("TP16",Track Package 16);
             };
        Boundary("End Extended WHSA Locality 2");
    };
};
```

1.3 Modeling results

The modeling results show the calibrated ^{14}C ages for the trackway-bearing sediments [TH(-3) through TP16] at WHSA Locality 2 consistently fall within the temporal bounds of the Last Glacial Maximum (~26 to 19 or 20 ka; *4*), regardless of which age model is applied:

Age Model Hypothesis 1: ~24.7 to 21.3 ka
Age Model Hypothesis 2: ~24.5 to 21.3 ka
Age Model Hypothesis 3: ~23.5 to 20.3 ka
Age Model Hypothesis 4: ~23.5 to 21.7 ka

Graphical representations of the `Deposition` and `Sequence` modeling results are shown in Figures S17 and S18, respectively, and the age distributions of the track horizons are provided in Figure S19. Modeled ages for all track horizons and packages, radiocarbon sample depths, and proxy sample depths are presented in Table S9.

1.4 Evaluating the age model hypotheses

To evaluate the four age model hypotheses, we compared the hydrogen isotopic composition of leaf waxes (δ^2H_{wax}) from samples collected throughout the WHSA Locality 2 sedimentary sequence to oxygen isotopic values from the Greenland ice cores. For the latter, we used the average $\delta^{18}O$ values between GISP2 and NGRIP to capture shared variability, both of which are on a synchronized GICC05 chronology (*21*). Previous work has shown clear teleconnections between the North Atlantic and southwestern U.S. existed during the late Pleistocene (*18-20, 38*), so we hypothesized that the highest level of agreement between these two independent records should reflect the most accurate age model. As a further test, the modeled age of a prominent spike in the leaf wax data observed at an elevation of 1196.50 at WHSA Locality 2 was compared to the age (23.30±0.60 ka) of the spike in the $\delta^{18}O$ values of the ice core record representing Dansgaard-Oeschger (D-O) event 2.

The results for Model 1 (*Ruppia* seed ages only, uncorrected) yielded an R value of only 0.02 and an age of 24.19±0.22 ka for the δD spike, neither of which indicate significant agreement

between the records (Fig. S20A). Similarly, the results for Model 2 (*Ruppia* seed ages and pollen ages, both uncorrected) yielded an R value of 0.09 and an age of 24.00±0.17 ka (Fig. S20B), again indicative of a lack of agreement between the records. For Model 3 (*Ruppia* seed ages only, corrected assuming a constant HWE), the R value was 0.25 and the age of the isotopic spike was 23.09±0.41 ka, which is statistically indistinguishable from the onset of D-O 2 (Fig. S20C). Finally, Model 4 (*Ruppia* seed ages only, corrected assuming a variable HWE) yielded the best results, with an R value of 0.40 and an age of 23.25±0.25 ka for the isotopic spike, which is nearly identical to the timing of D-O 2 in the Greenland ice core record (Fig. S20D).

Based on the correlation between the two independent isotopic records and agreement between the timing of the isotopic spike in the WHSA Locality 2 record and the onset of D-O 2 in the NGRIP isotopic record, we prefer Model 4 and used those results when shaping the discussion and creating the figures presented in the main text (Fig. S21).

To determine how well WHSA Locality 2 and the ice core data match while accounting for age uncertainty, we generated 1000 possible age models for each time series, respectively. For WHSA Locality 2, we re-ran Model 4 using BACON (rbacon) (*69*) and sampled 1000 age models from the posterior distribution of generated by BACON's MCMC algorithm. For the GICC05 ice chronology, we generated 1000 age models by Monte Carlo sampling of the counting uncertainty (while preserving the correlation of counting uncertainty downcore). We then calculated the correlation between each of the set 1000 time series for a total number of correlation (r) values of 1,000,000. The significance threshold of the correlation was assessed using the non-parametric method of (*22*).

2. Biomarker analysis

2.1 GDGTs

From the GDGT peak areas we computed the following indices to infer changes in temperature and salinity:

The 5-methyl branched temperature index (MBT'_{5ME}) based on branched GDGTs (*70*):

$$MBT'_{5ME} = \frac{brIa + brIb + brIc}{brIa + brIb + brIc + brIIa + brIIb + brIIc + brIIIa + brIIb}$$

The Archaeol-Caldarchaeol Econometric (ACE) based on archaeol and isoGDGT-0 (*26*):

$$ACE = \frac{Archaeol}{Archaeol + isoGDGT0}$$

The Isomerization ratio of 6- and 7-methyl isomers based on branched GDGTs (IR_{67}) (*27*):

$$IR_{67} = \frac{IR_{6ME} + IR_{7Me}}{2}$$

where:

$$IR_{6ME} = \frac{brIIa' + brIIb' + brIIc' + brIIIa' + brIIIb'}{brIIa + brIIb + brIIc + brIIIa + brIIIb + brIIa' + brIIb' + brIIc' + brIIIa' + brIIIb'}$$

and:

$$IR_{7ME} = \frac{brIIIa''' + brIIa'''}{brIIIa + brIIIa' + brIIIa''' + brIIa + brIIa' + brIIa'''}$$

In saline lake basins, salinity influences MBT'_{5ME}, which can result in an overestimate of temperature from this proxy (*27*). Wang et al. (*27*) proposed a method to correct for this effect using IR_{67} which we followed with some modifications. First, we fit a multiple linear regression model to the published salty lake dataset of Wang et al. (*27*) to predict MBT'_{5ME} from IR_{67} and their provided mean lake water temperature (which is equivalent to the mean annual air temperature above freezing (MAF); i.e., mean annual temperature excluding months below 0˚C):

$$MBT'_{5ME} = a + b1 * IR_{67} + b2 * MAF$$

The coefficient *b1* is 0.67 +/- 0.03 (1σ), which is the sensitivity of MBT'_{5ME} to salinity, as represented indirectly by IR_{67}. This is then used to correct our MBT'_{5ME} values measured at White Sands for salinity changes as follows:

$$MBT_{corr} = MBT'_{5ME} - 0.67 * IR_{67}$$

We then calibrated the corrected MBT'_{5ME} values to mean temperature above freezing using the BayMBT lakes calibration of (*25*). We then calibrated the corrected MBT'5ME values to mean temperature above freezing (MAF) using the BayMBT lakes calibration of (33). The lakes calibration relates MBT'_{5ME} to MAF because it assumes that the bacterial producers can only be active in liquid water in a lake. If winter temperatures did not drop below freezing at White Sands during glacial times, then MAF is equivalent to mean annual air temperature (MAAT). If the winter temperatures did drop below freezing, then glacial MAF is warmer than MAAT and glacial cooling would be underestimated. Present day winter temperatures at White Sands are 6 ˚C, so this scenario would require glacial cooling greater than this to occur. Independent noble gas analyses suggest that glacial cooling in the New Mexico region was 5.5 ˚C (*71*), which is fairly similar to what the MBT temperatures estimate here and would suggest that there were not prolonged freezing temperatures in winter.

We prefer this approach over using the multiple linear regression equation above because the Wang et al. (*27*) dataset consists of high-altitude lakes from China that have a limited temperature range, so the temperature sensitivity *b2* is underestimated (in contrast, the dataset spans a wide range of salinities so should capture *b1* well). Conversely, the BayMBT calibration spans the full range of lake temperatures across the globe and anomalous hypersaline lakes are omitted, so it provides the best estimate of *b2*.

To test whether this temperature inference method is robust, we analyzed modern playa sediments in WHSA from an intermittently wet playa located at 32.798 ˚N, 106.212 ˚W. Two surface samples were taken from the playa and GDGTs were analyzed using the same methods listed above and resulted in similar values; average IR_{67} from these sediments was 0.53 and average MBT'_{5ME} was 0.92 (Table S10). Using the method above, the corrected MBT'_{5ME} value was 0.56, which using BayMBT yields a MAF (equivalent to mean annual temperature) of 16˚C, which matches the mean annual temperature measured at Holloman Air Force Base and Tularosa, New Mexico (also 16˚C). Assuming that the paleoenvironment was similar, this modern test suggests that the temperature inference approach taken here is valid for the ancient playa sediments.

We also computed the probability of the type of depositional environment using the branched and isoprenoid GDGT machine learning classification algorithm (BIGMaC) (*28*). This algorithm outputs the probability of four types of environments based on the distribution of GDGTs: “Soil-Type”, “Marine-Type”, “Lake-Type”, and “Peat-Type”. The “-Type” acknowledges that these classifications are not always interpreted literally; for example, in the context of White Sands, the “Marine-Type” classification does not mean that the paleoenvironment was marine but rather points towards saltier conditions which mimic the assemblages found in the marine environment. For clarity we refer to the “Marine-Type” class as “Salty GDGTs” when discussing this category.

The BIGMaC algorithm predicts that the dominant GDGT environments are “Lake-Type” and “Soil-Type”, with “Lake-Type” yielding a higher probability in the deeper section of the sedimentary sequence and “Soil-Type” vice versa, in agreement with the lithological interpretation that the deeper part of the section is a lake and shallower portion a playa that is increasingly drying through time (Fig. S23). “Peat-type” probabilities are uniformly low suggesting this environment was never a peatland. “Salty GDGTs” jump up in abundance at select intervals in the sequence and are associated with drying events, which we interpret as more saline conditions (Figs. 4B, S23). GDGT results are provided in Table S11.

2.2 δ^2H of leaf waxes

δ^2H_{wax} ranges from -180‰ to -150‰ with standard errors between 0.3‰ and 2.1‰ (Fig. 3B, Table S12). To convert these values to an estimate of δD of precipitation (δD_p) we had to determine the apparent fractionation value (ε) between rain water and leaf wax, which can vary as a function of plant type and biome (*23*). To do this for the White Sands area we turned to our modern playa sediments and analyzed these for δ^2H_{wax}. Modern average δ^2H_{wax} is -144‰ (Table S12). There are no precipitation isotope monitoring stations in the immediate vicinity of White Sands National Park nor anywhere in New Mexico at lower elevations. Thus, we use the long-term monitoring station in Tucson, Arizona (*72*) to estimate δ^2H_p. Tucson and White Sands have a similar climatology, although White Sands is slightly cooler year-round due to its higher elevation (1241 m vs. Tucson’s 750 m) and receives less winter precipitation (Fig. S24). The latter may be important for mean annual δ^2H_p since winter precipitation in the southwestern US is typically more depleted (Fig. S24). We therefore took the monthly values of δ^2H_p from Tucson and then calculated the weighted mean annual average using monthly precipitation at White Sands, which yields a value of -50‰. This is nominally different from the Tucson average of -53‰ but might be slightly more accurate for a more summer-biased rainfall climatology. Using -144 ‰ and -50‰ as our modern values for δ^2H_{wax} and δ^2H_p respectively, ε is computed as:

$$\varepsilon = \frac{1000 + \delta^2 H_{wax}}{\frac{\delta^2 H_p}{1000} + 1} - 1000$$

Resulting ε is -100‰, which is very similar to the average apparent fractionation value of -94‰ determined for a variety of biomes in southern California, including the Mojave Desert (*73*). We used this value for computing δ^2H_p from δ^2H_{wax} for our data from WHSA Locality 2, which results in a range of values between -90‰ and -60‰ (Fig. 4B).

3. Ostracode analysis

Ostracodes were present only in the lower part of the WHSA Locality 2 depth profile, specifically in samples 22WHSA1-18.1r, .1t, .1u, .1v2, .1v4, and .1w (Table S15; Fig. S26). There were no ostracodes, mollusks, charophytes, or shell fragments in any of the other samples we analyzed. A total of 1726 ostracode valves were identified, which consisted of only two species: *Limnocythere staplini* Gutentag & Benson, 1962 (Fig. S27) and *Cyprideis salebrosa* van den Bold, 1963. Both of these taxa are benthic species that are found living today in oxygenated, shallow lakes, marshes and wetlands at the sediment/water interface. In addition, in some samples the foraminifera *Cribroelphidium* sp. cf. *gunteri* Cole, 1931 occurs, and occasionally the calcified oogonia of macrophytic Charophyte algae are also found. Where ostracodes occur in the samples, the shells are in good condition, with little to no evidence of reworking. Adults co-occur with juveniles, giving a good indication of populations living at that time.

Miscellaneous notes

Any use of trade, firm, or product names is for descriptive purposes only and does not imply endorsement by the U.S. Government.

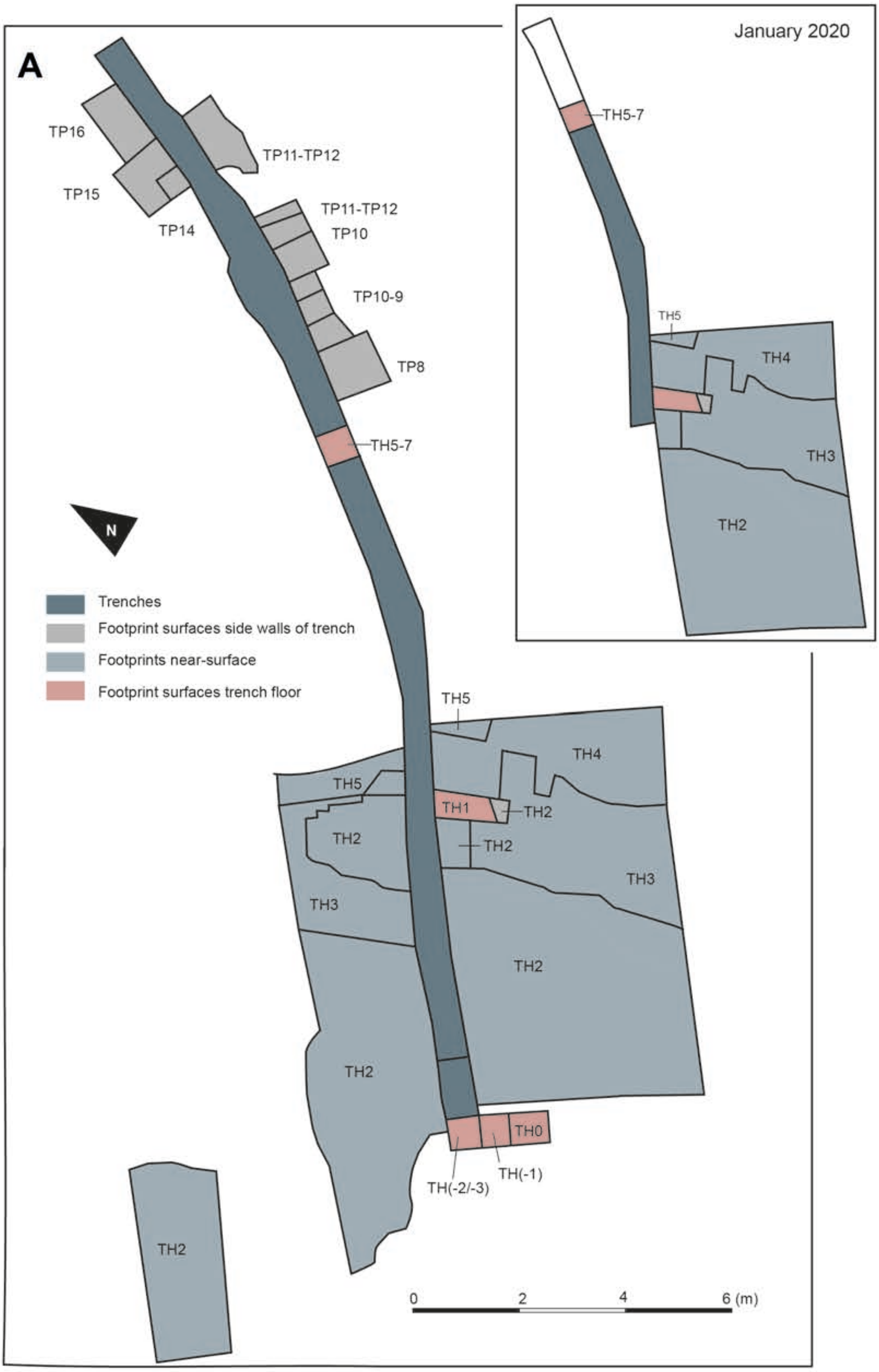

Fig. S1. Excavated surfaces at WHSA Locality 2. The inset figure shows the extent of the excavation completed in January 2020, with that completed in January 2022 shown in the main figure. Note the precise location of the site is withheld in accordance with the statutory responsibilities of the National Park Service who can be contacted for a precise location if required for research purposes.

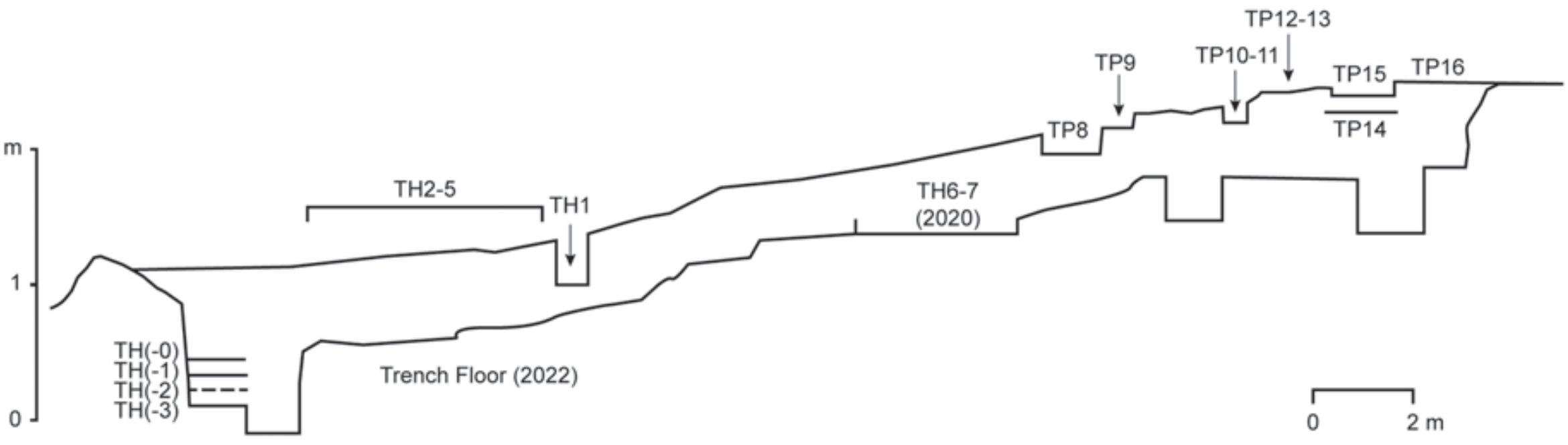

Fig. S2. Profile of the trench from west (left) to east (right) in January 2022 showing the location and approximate elevation of the footprint surfaces. Derived from DEM created via photogrammetry.

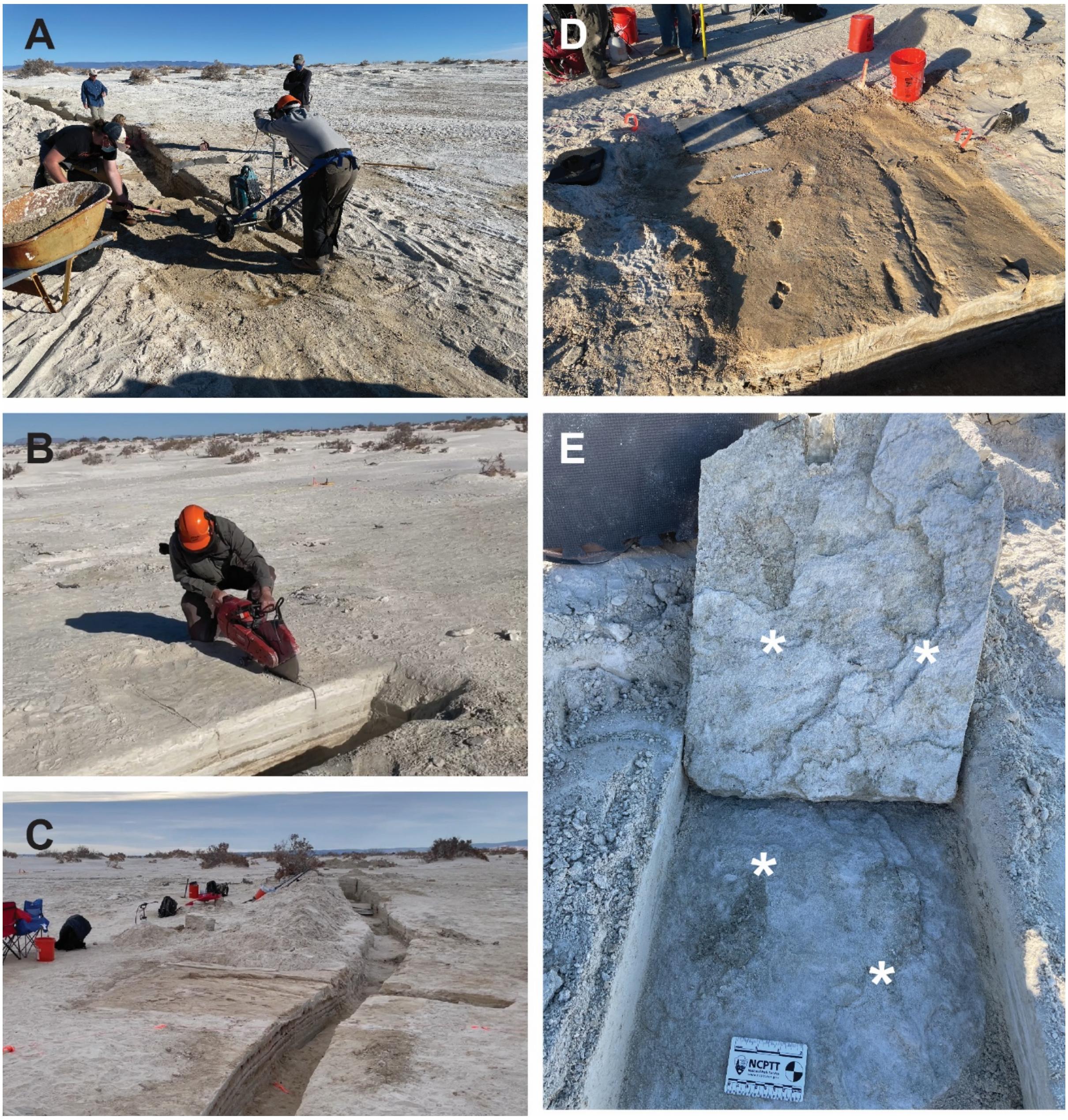

Fig. S3. Photographs showing excavation processes. (**A**) Using the mini-trenching tool to cut the main trench. (**B**) Using the circular rock saw to define blocks to be hinged. The sediment surface and some of the gypsum-rich layers are simply too indurated for more conventional approaches. (**C**) General view of the main trench in January 2022 showing a typical example of a near-surface footprint horizon to the left of the main trench. (**D**) Initial work on the area shown in (C). (**E**) An example of a hinged-slab. The asterisks show matching points on two unexcavated footprints just as they are found when the slab was first hinged upwards. Photo Credits: Matthew Bennett, Bournemouth University.

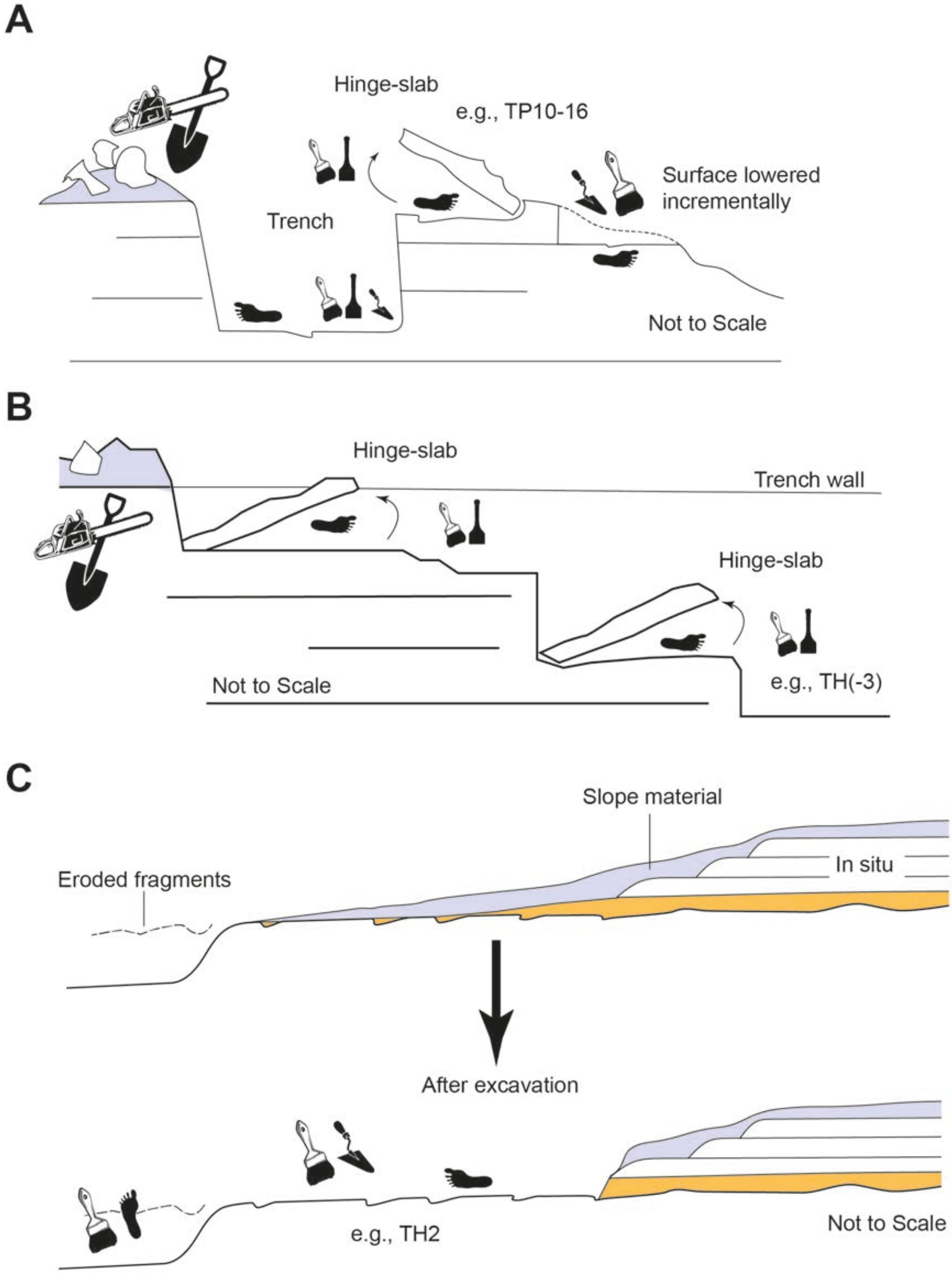

Fig. S4. Schematic illustration of the excavation methods used at WHSA Locality 2. The different colors are used only to depict different sedimentary layers. (**A**) Methods used at the eastern end of the main trench where most of the footprint surfaces were recorded on the shoulders of the main trench. (**B**) Schematic section along the long axis of the main trench showing how we levered up slabs from the floor to reveal tracks at the western end of the trench. (**C**) In the central section of the main trench and either side of it undisturbed tracked surfaces outcrop close to the surface as shown.

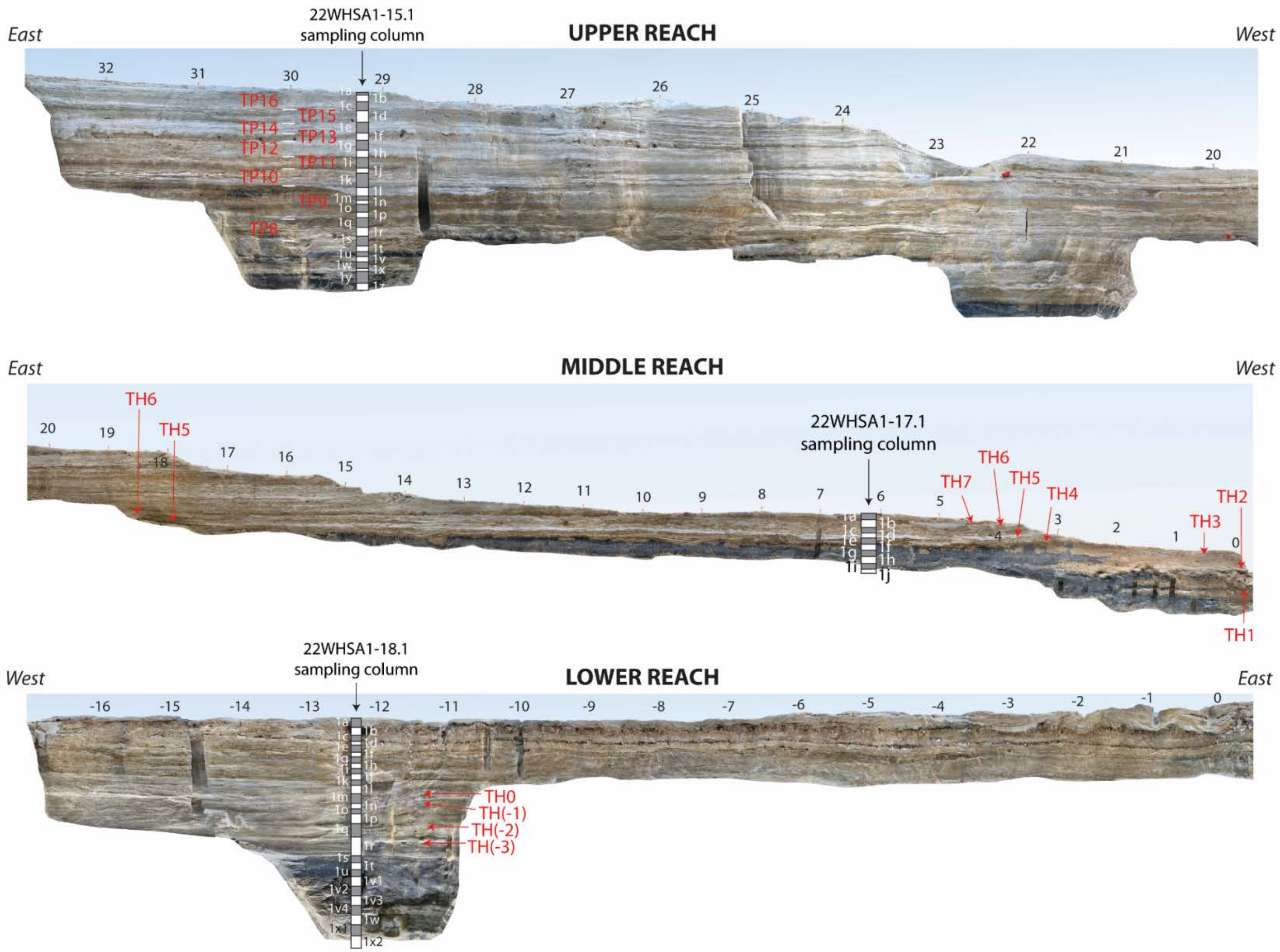

Fig. S5. Photomosaics of the main east-west trench at WHSA Locality 2 showing tracked horizons and sampling locations. Views are toward the north-facing wall for the upper and middle reaches, and toward the south-facing wall for the lower reach. Stations 32 through -16 are spaced 50 cm apart. Photo Credits: Jeff Pigati, U.S. Geological Survey.

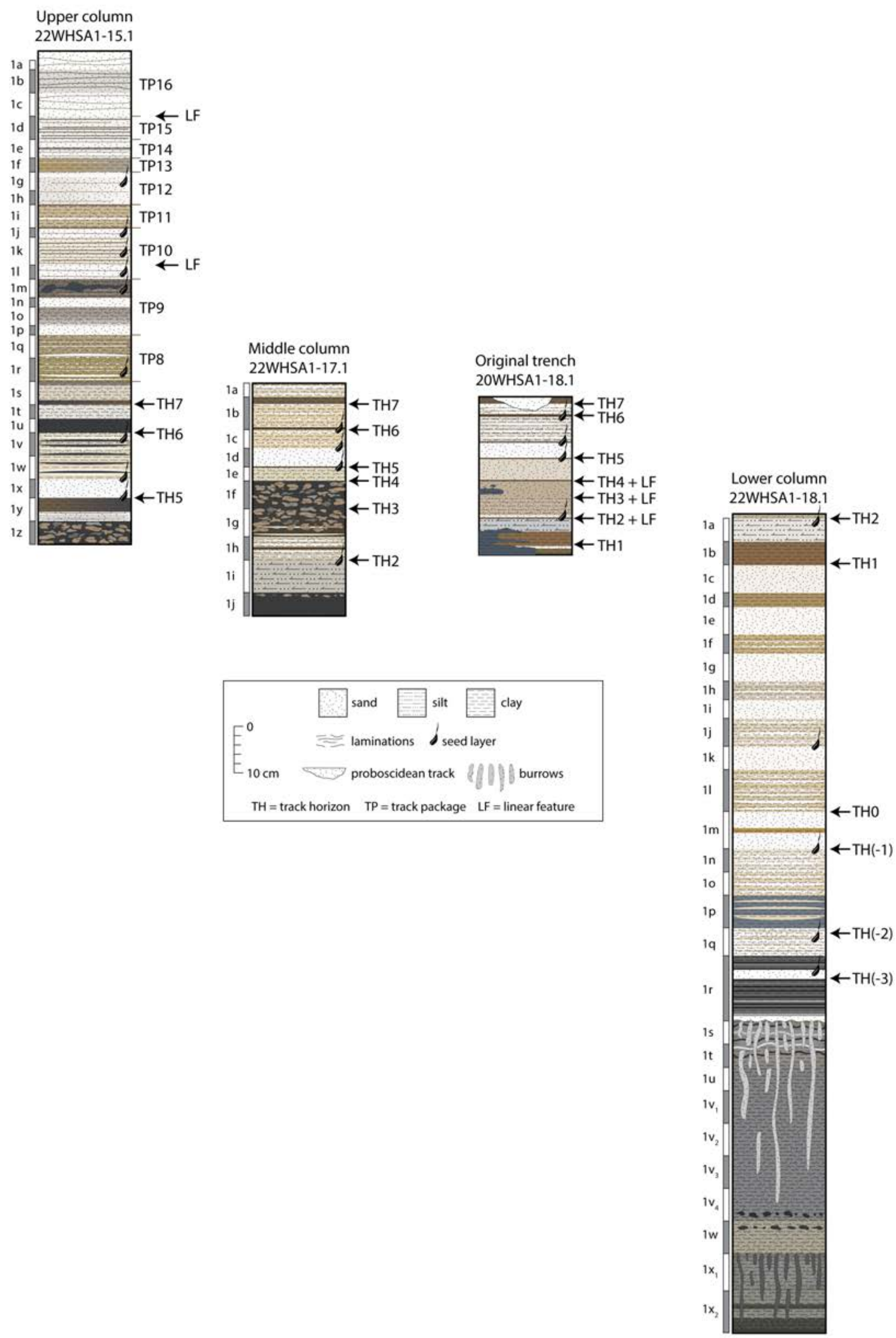

Fig. S6. Individual stratigraphic sections for the upper, middle, and lower reaches where proxy samples were collected as well as the stratigraphy of the original trench published by Bennett et al. (*1*).

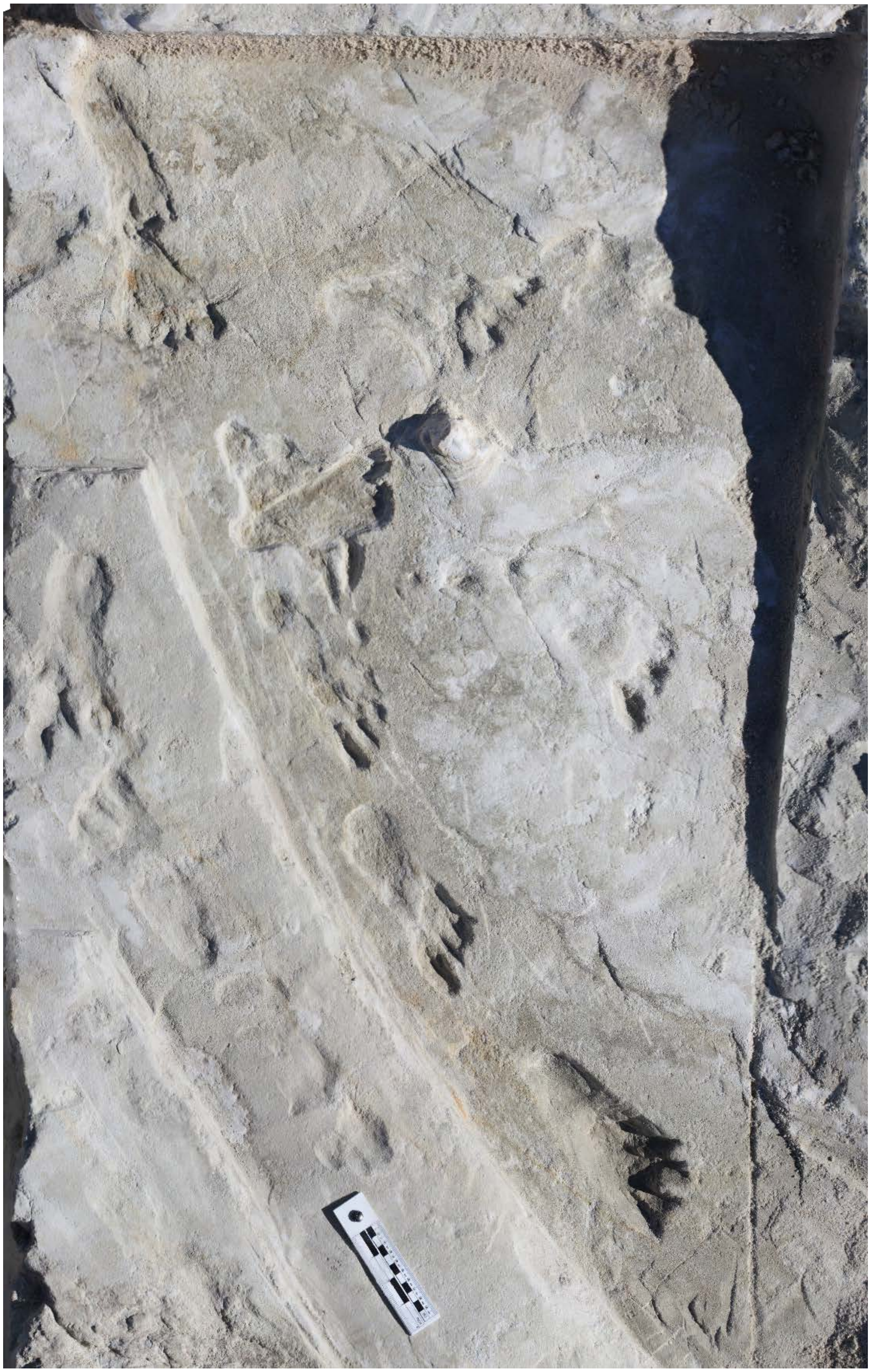

Fig. S7. Orthomosaic of the surface of TP15. This is a hard indurated layer with multiple tracks on thinly bedded gypsum sheets. There are also areas, including track fills, that are composed of dolomite. Photo Credit: Matthew Bennett, Bournemouth University.

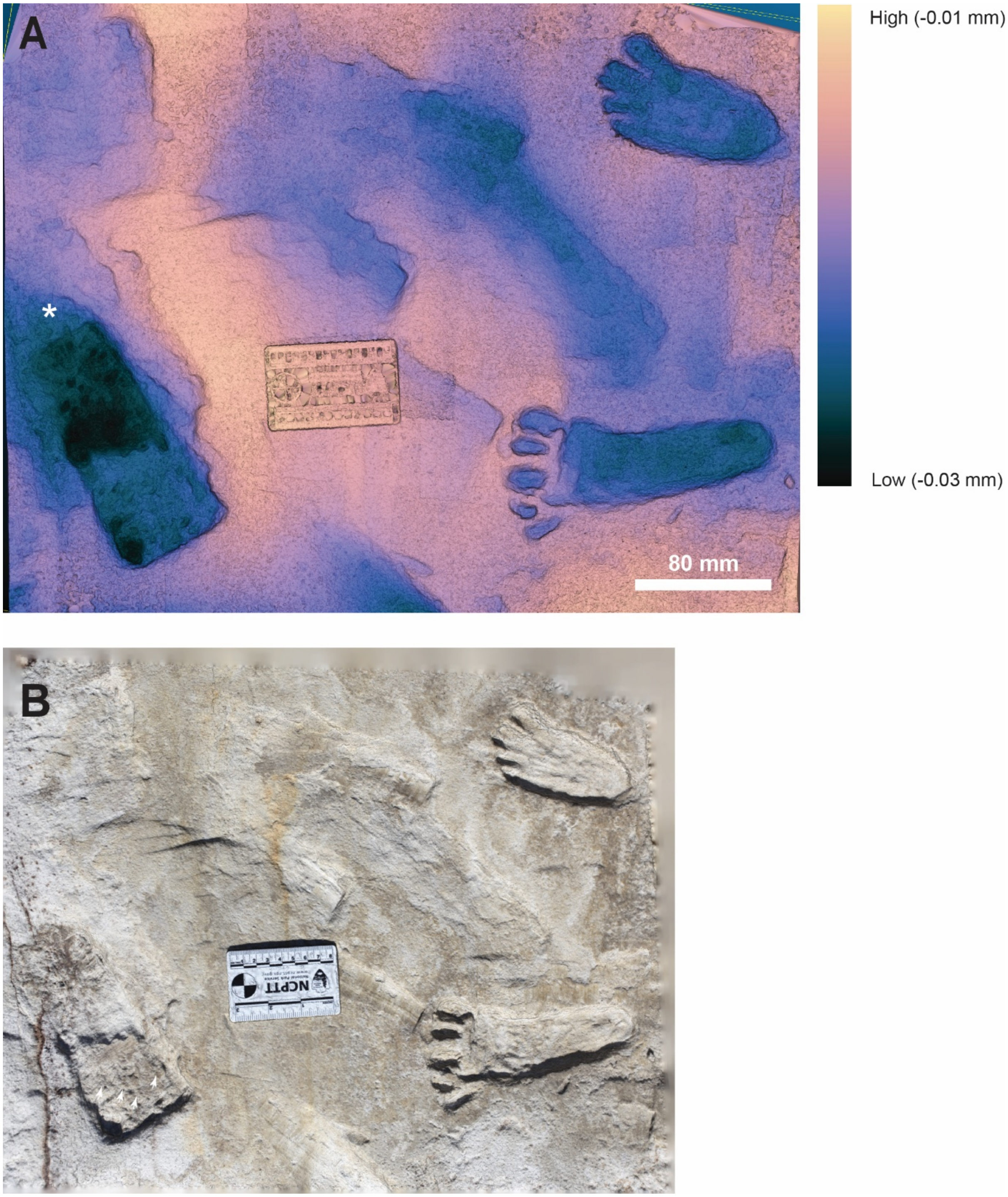

Fig. S8. Tracks on TP14. This is an indurated surface in which shallow track outlines are visible but difficult to excavate with confidence. The lower left track (asterisk in upper frame) in both frames is the exception, as the surface broke out of the track to reveal a softer substrate, and may show evidence of a woven sandal impression. Visible are ribs running the length of the track. (**A**) False color depth rendered surface with light colors showing elevated areas. (**B**) Orthomosaic of tracks on the same surface.

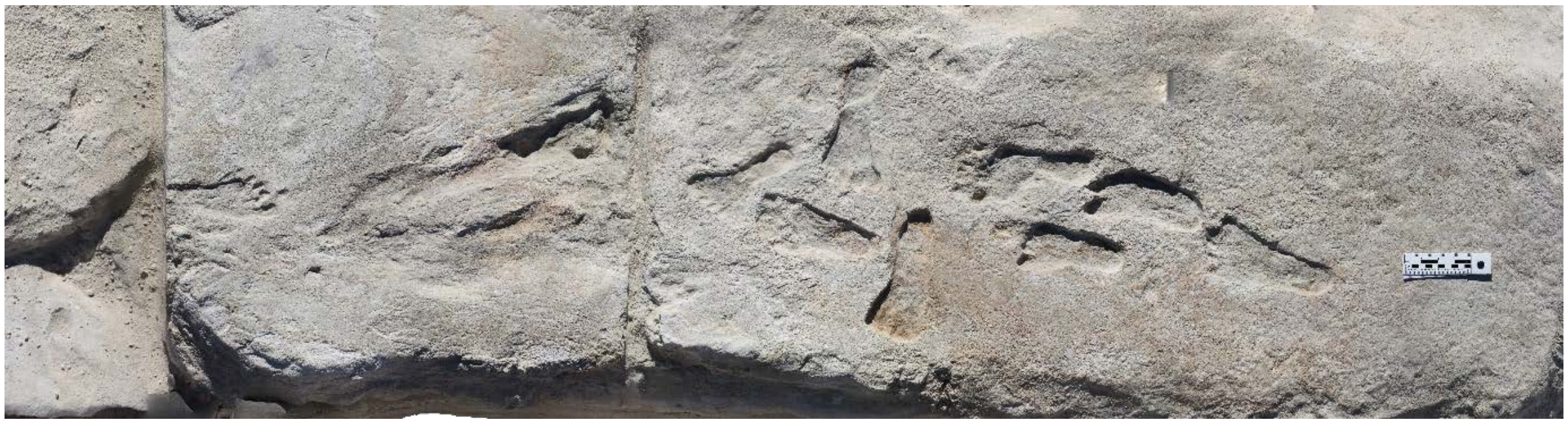

Fig. S9. Orthomosaic of tracks on TP12. This is an indurated surface in which several shallow track outlines are visible with varying directions. Photo Credit: Matthew Bennett, Bournemouth University.

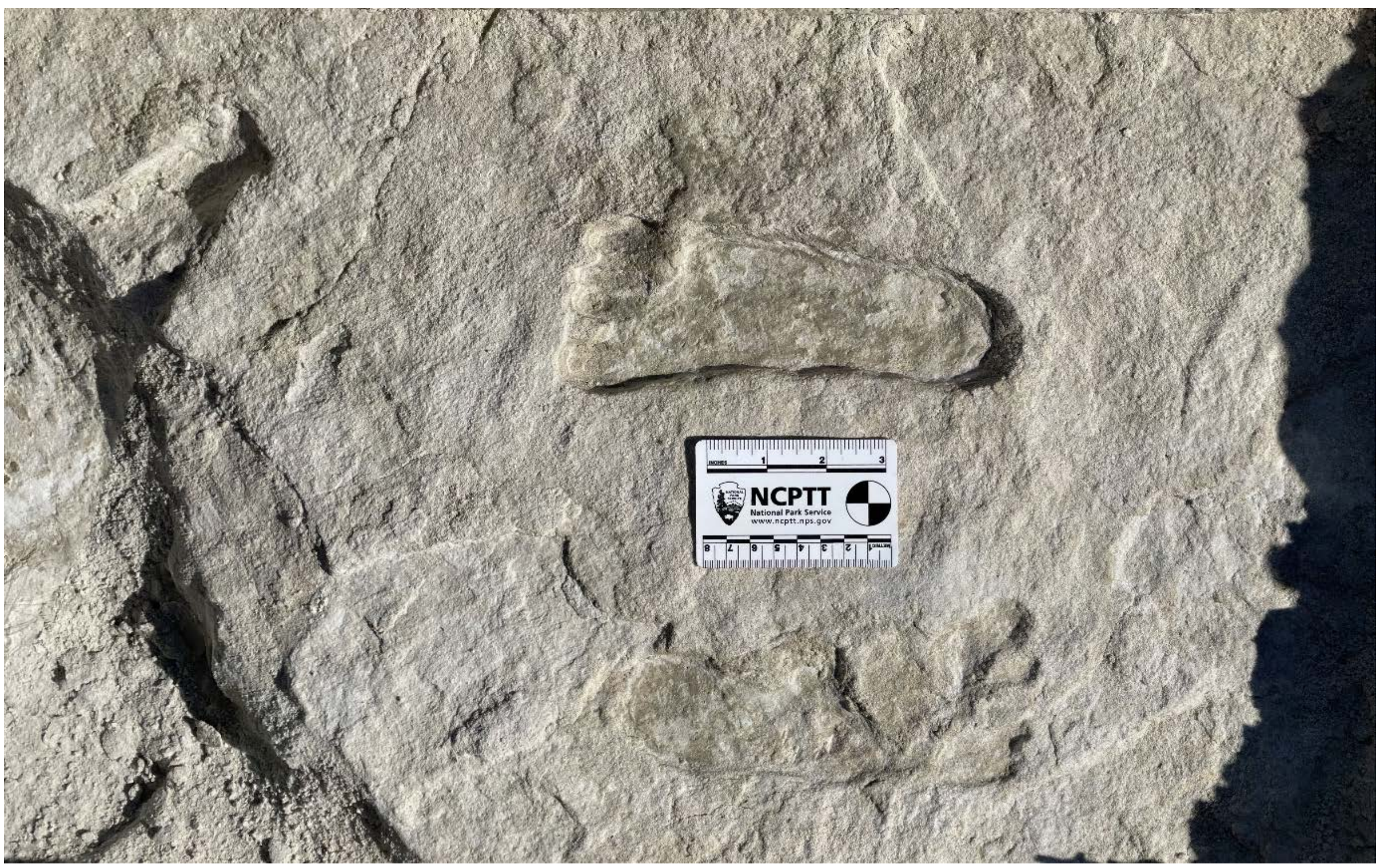

Fig. S10. Orthomosaic of tracks on TP11, these are the same tracks as in Figure S3E. Photo Credit: Matthew Bennett, Bournemouth University.

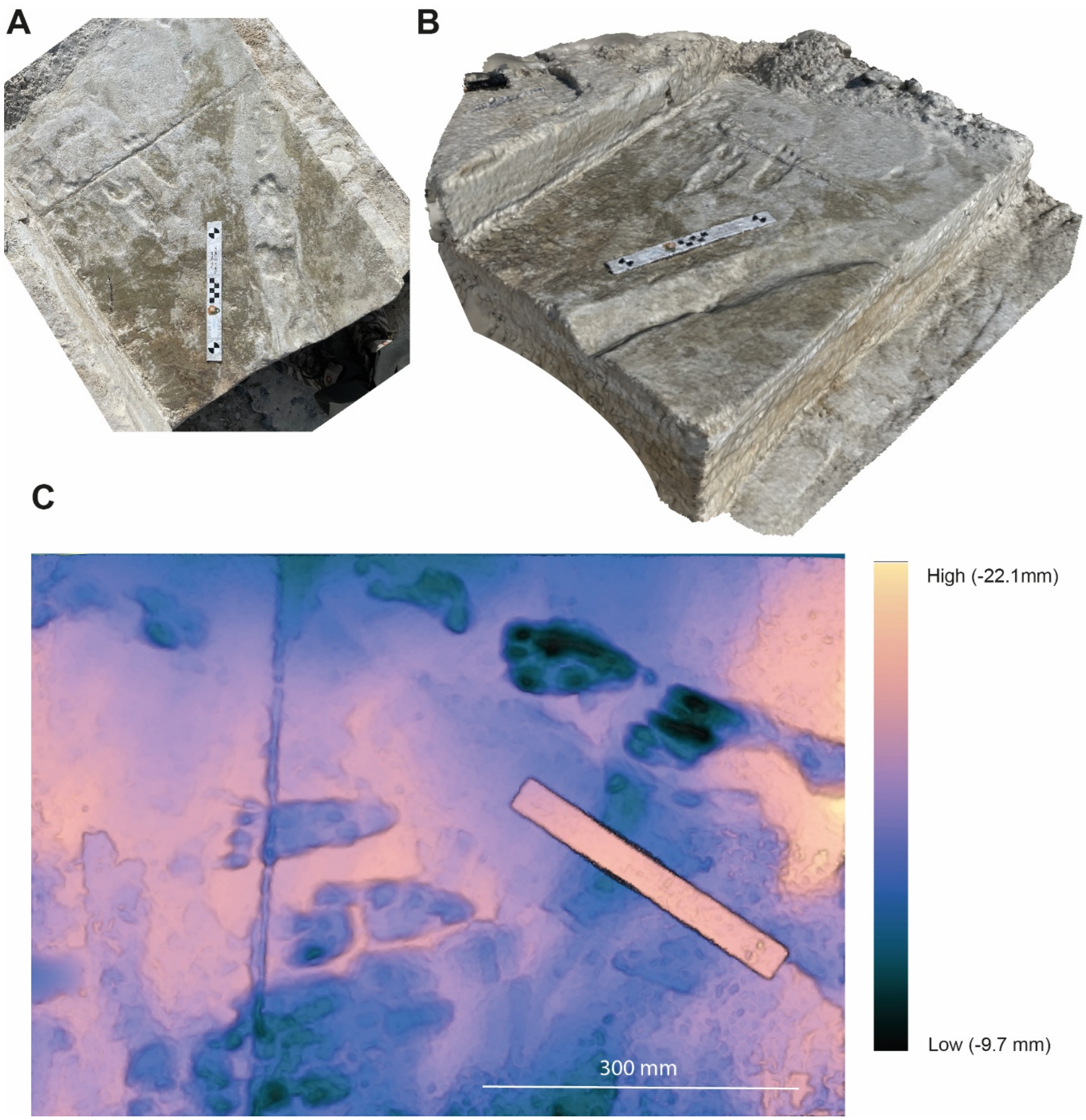

Fig. S11. Three dimensional models of TP9. (**A**) The initial surface with tracks superimposed on an infilled linear feature. (**B**) The surface after that linear feature was excavated. (**C**) Color rendered three-dimensional model of the initial surface (i.e., panel A). Photo Credits: Matthew Bennett, Bournemouth University.

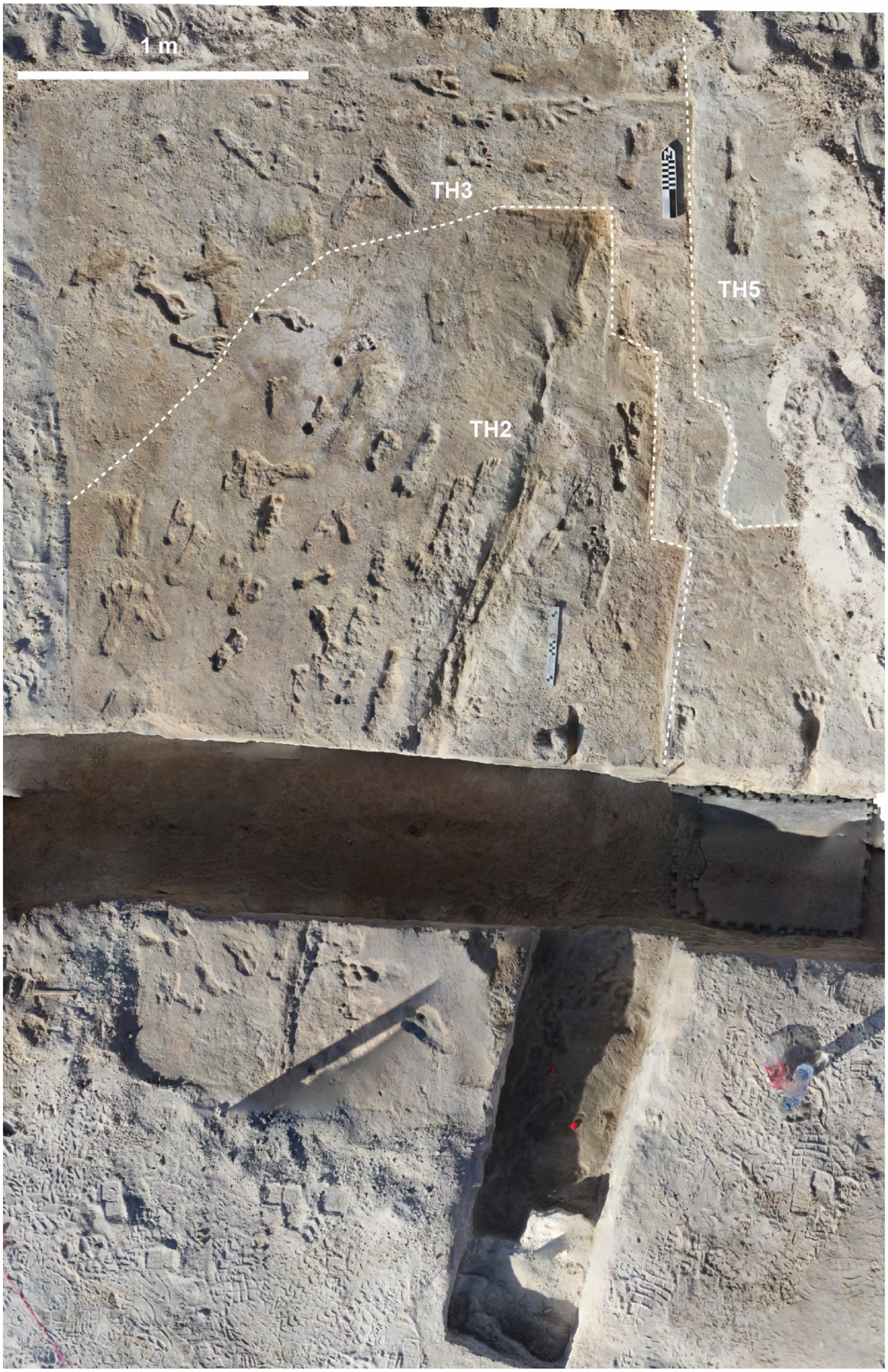

Fig. S12. Orthomosaic of tracks on TH2 and TH3. A linear feature on TH2 splits the surface north-south. Photo Credit: Matthew Bennett, Bournemouth University.

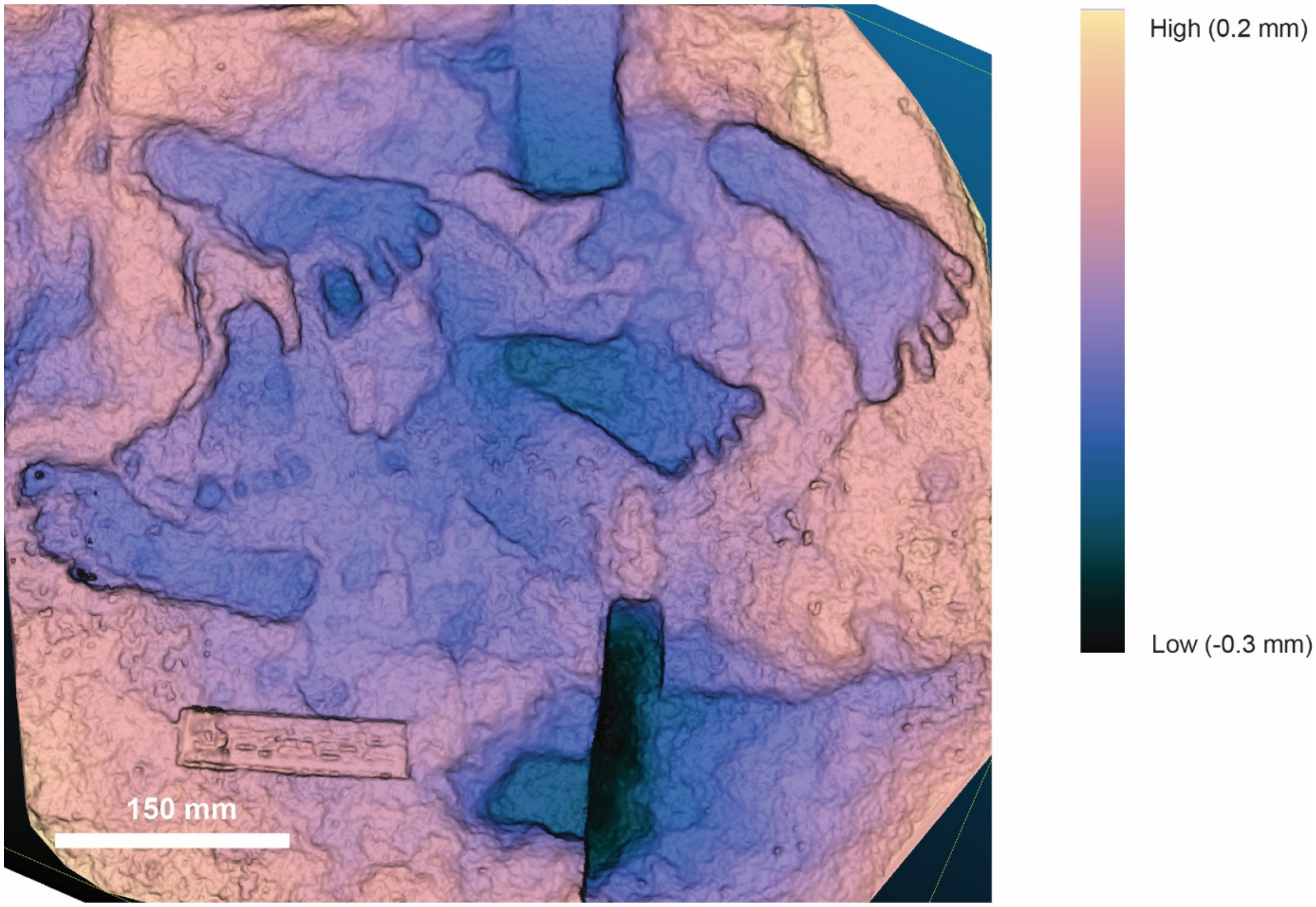

Fig. S13. Track horizon TH0 showing a trampled surface with multiple human tracks of various sizes. False color image with light colors showing elevated areas. Note the missing sediments at the top and bottom of the image which were created by the dirt saw during excavation.

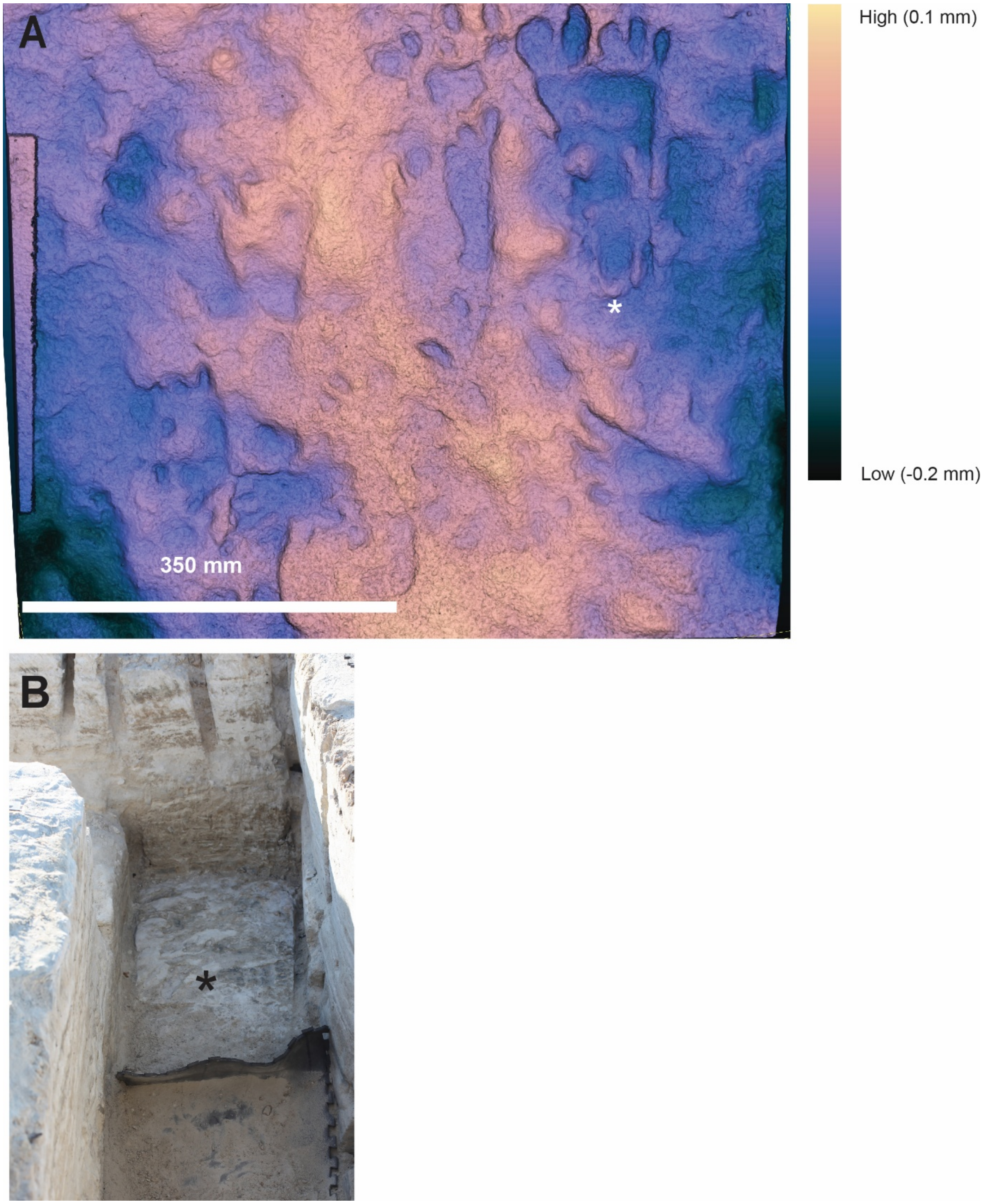

Fig. S14. (**A**) TH(-3) showing a trampled horizon with a large right foot, several toe impressions and a few smaller partial human tracks. In the bottom left-hand corner of this surface (just above the scale bar) is a collection of small, superimposed tracks which may reflect the shuffling movement and foot rotation of a child. (**B**) Location of the surface at the base of the main trench; asterisk shows location of the track's heel marked in panel A. Photo Credit: Matthew Bennett, Bournemouth University.

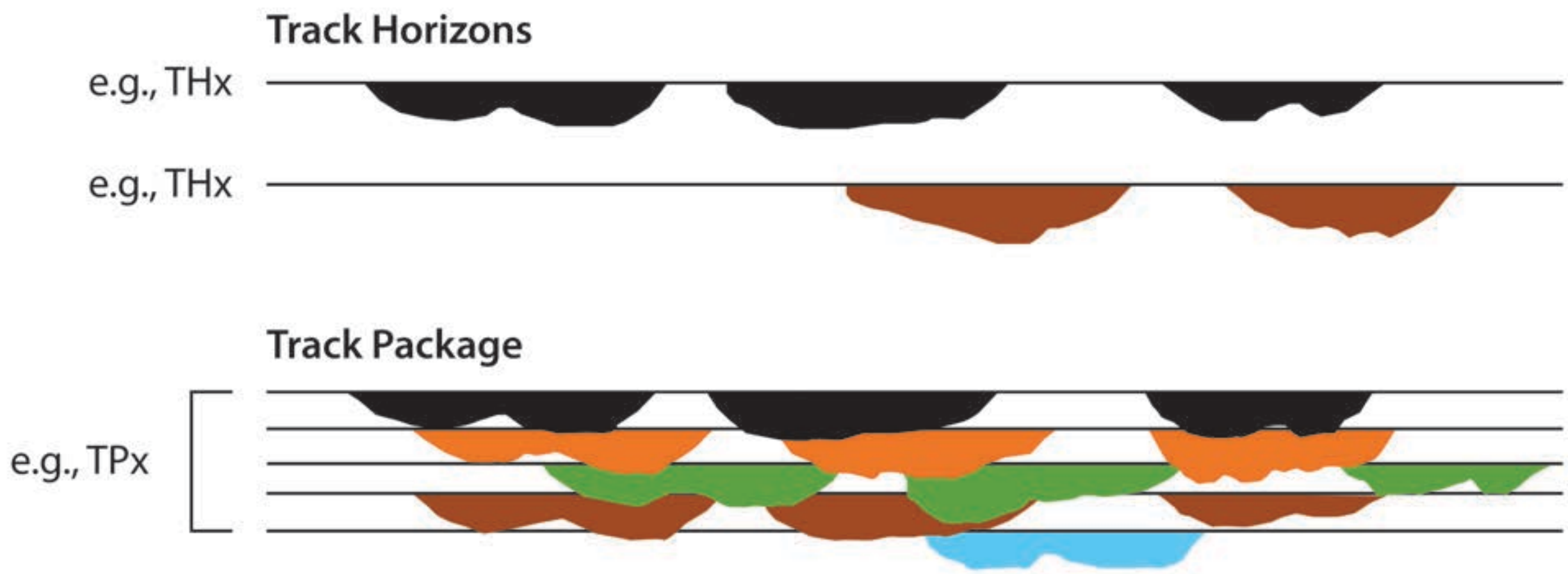

Fig. S15. Schematic drawing showing the difference between individual track horizons and a track package.

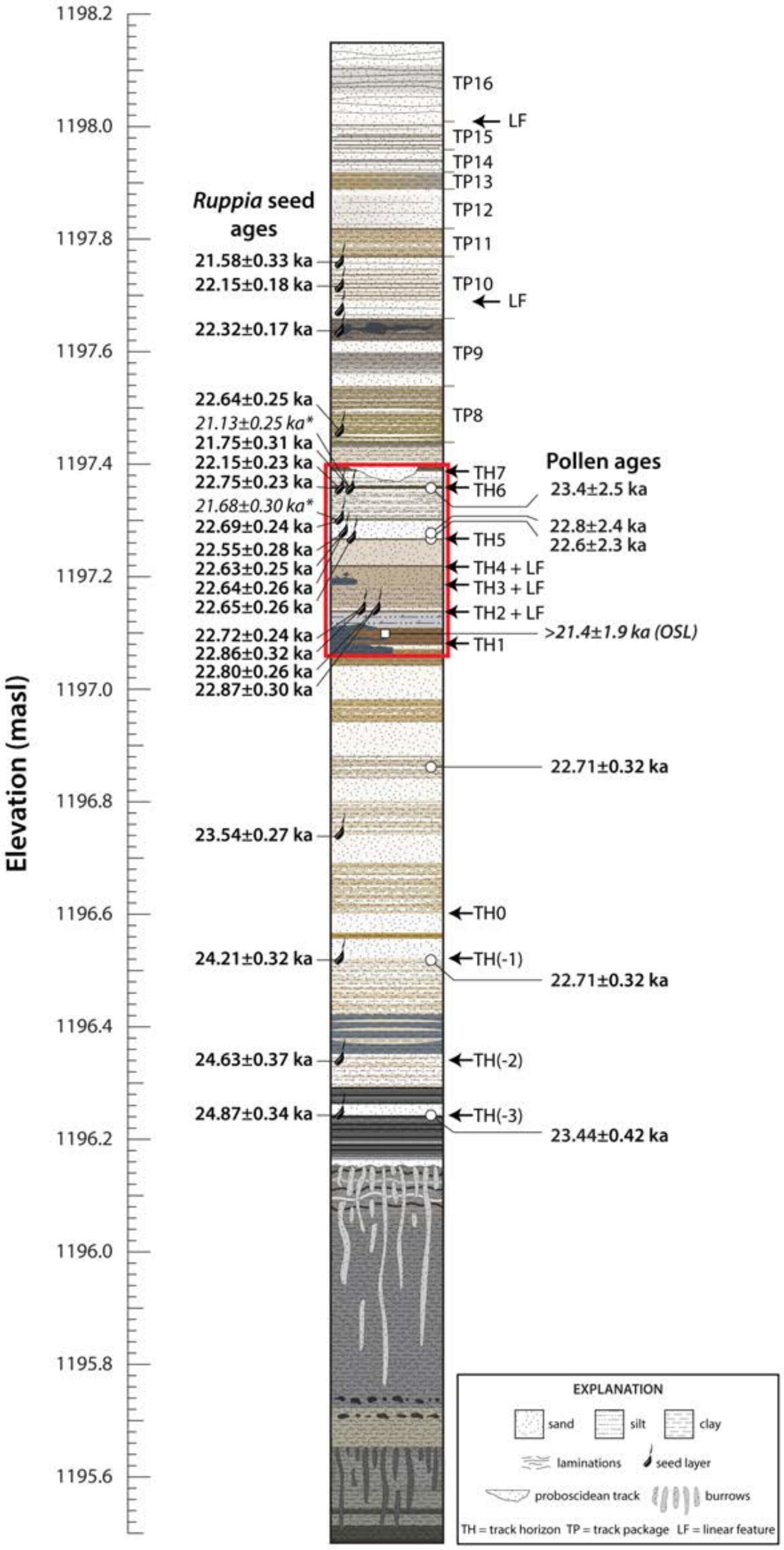

Fig. S16. Composite stratigraphic section showing the locations of calibrated ^{14}C ages of *Ruppia* seeds and pollen, minimum OSL age, track horizons and packages, linear features, and elevation. The red box depicts the extent of the stratigraphy at WHSA Locality 2 containing track horizons reported previously (*1*).

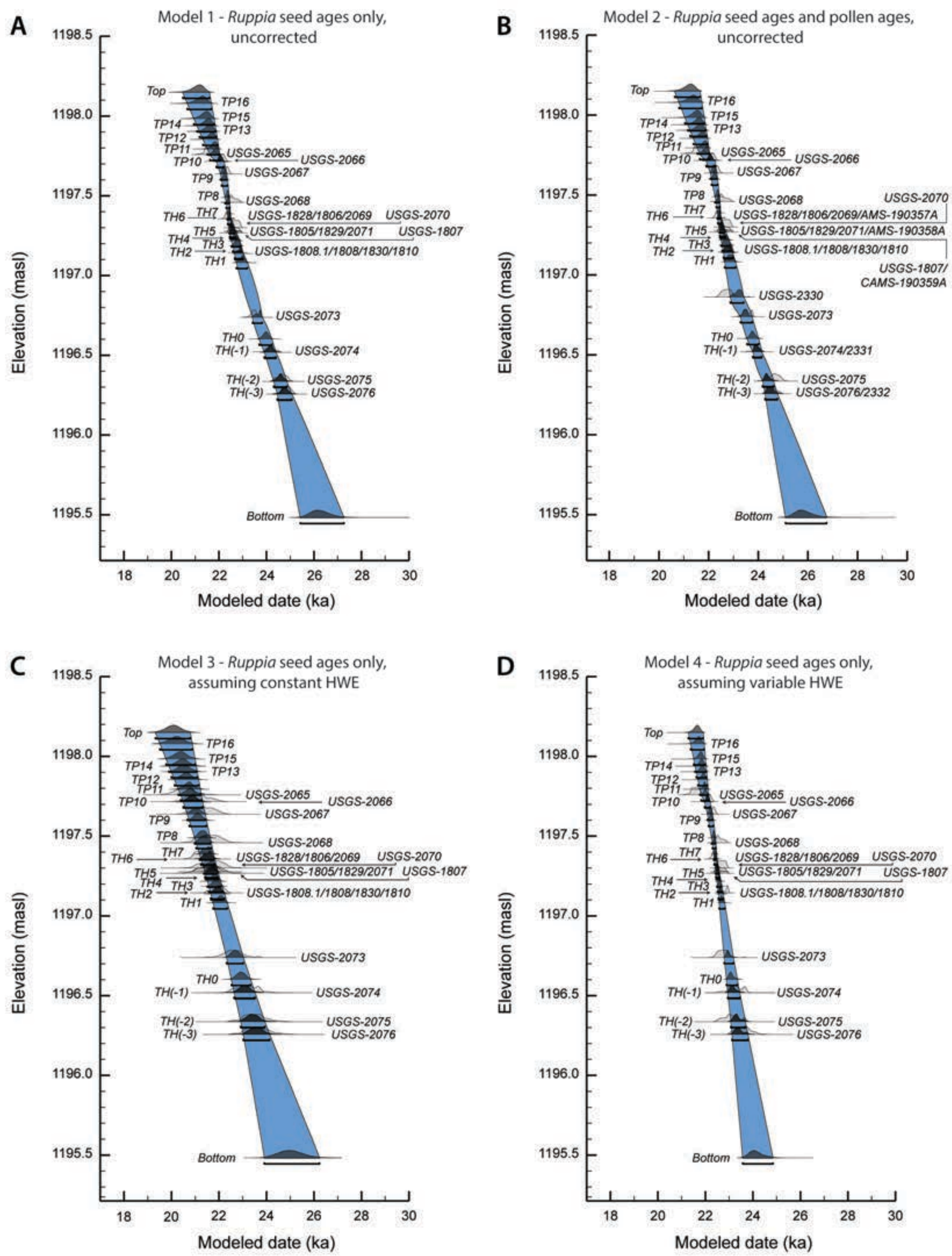

Fig. S17. Modeled ages using the `Deposition` function in OxCal for the track-bearing sequence at WHSA Locality 2 for (**A**) Model 1: *Ruppia* seed ages only, uncorrected, (**B**) Model 2: *Ruppia* seed ages and pollen ages, both uncorrected, (**C**) Model 3: *Ruppia* seed ages only, corrected assuming a constant hard-water effect (HWE), and (**D**) Model 4: *Ruppia* seed ages only, corrected assuming a variable hard-water effect. All figures were generated using OxCal v.4.4.4 (*57*) and the IntCal20 calibration curve (*58*); accessed online 20 July 2025. Samples USGS-1803 and USGS-1804 were identified as outliers at the 95% confidence level, so were excluded from the analysis.

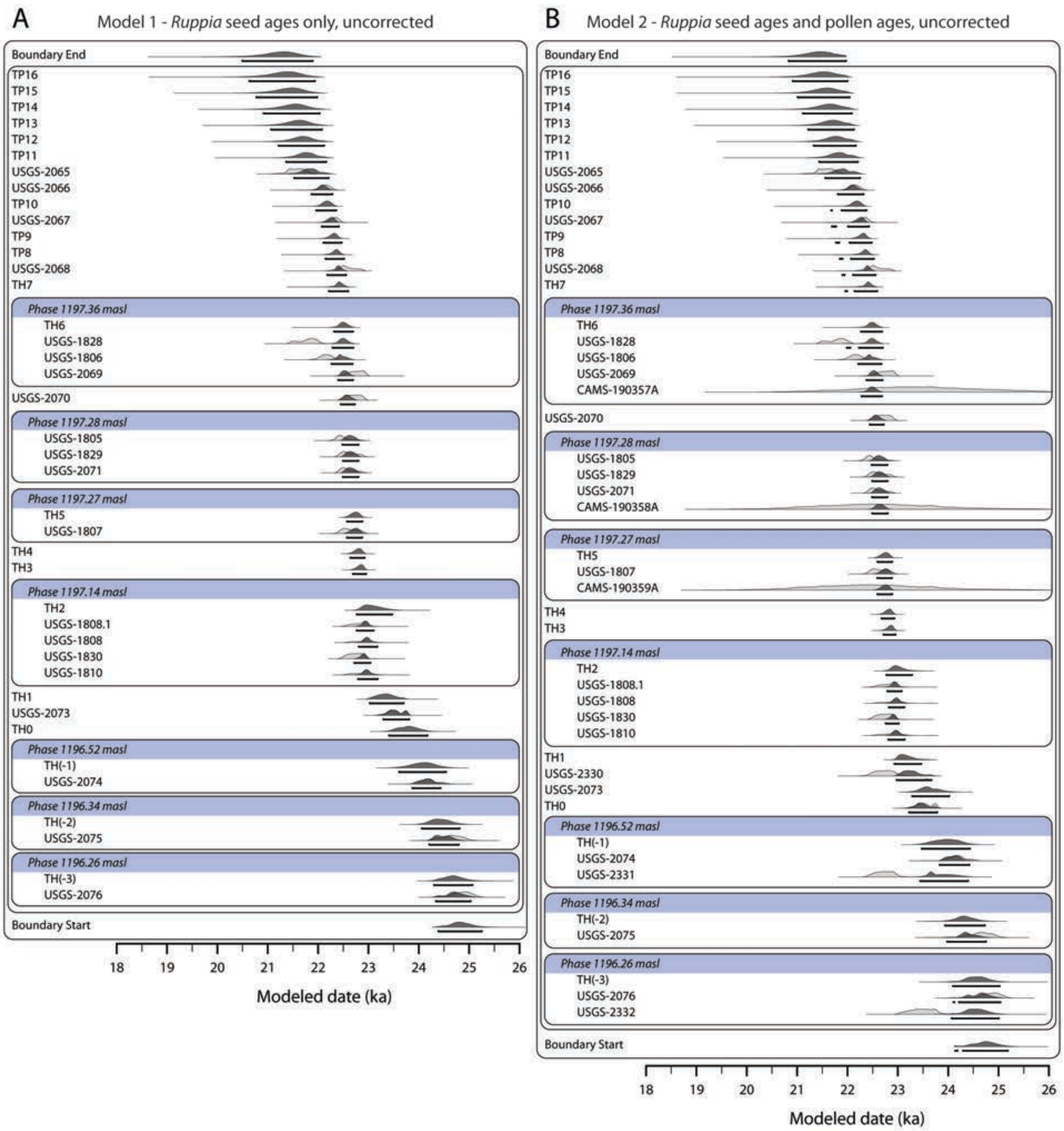

Fig. S18. Modeled ages using the `Sequence` function in OxCal for the track-bearing sequence at WHSA Locality 2 for (**A**) Model 1: *Ruppia* seed ages only, uncorrected, (**B**) Model 2: *Ruppia* seed ages and pollen ages, both uncorrected. All figures were generated using OxCal v.4.4.4 (*57*) and the IntCal20 calibration curve (*58*); accessed online 20 July 2025. Samples USGS-1803 and USGS-1804 were identified as outliers at the 95% confidence level, so were excluded from the analysis.

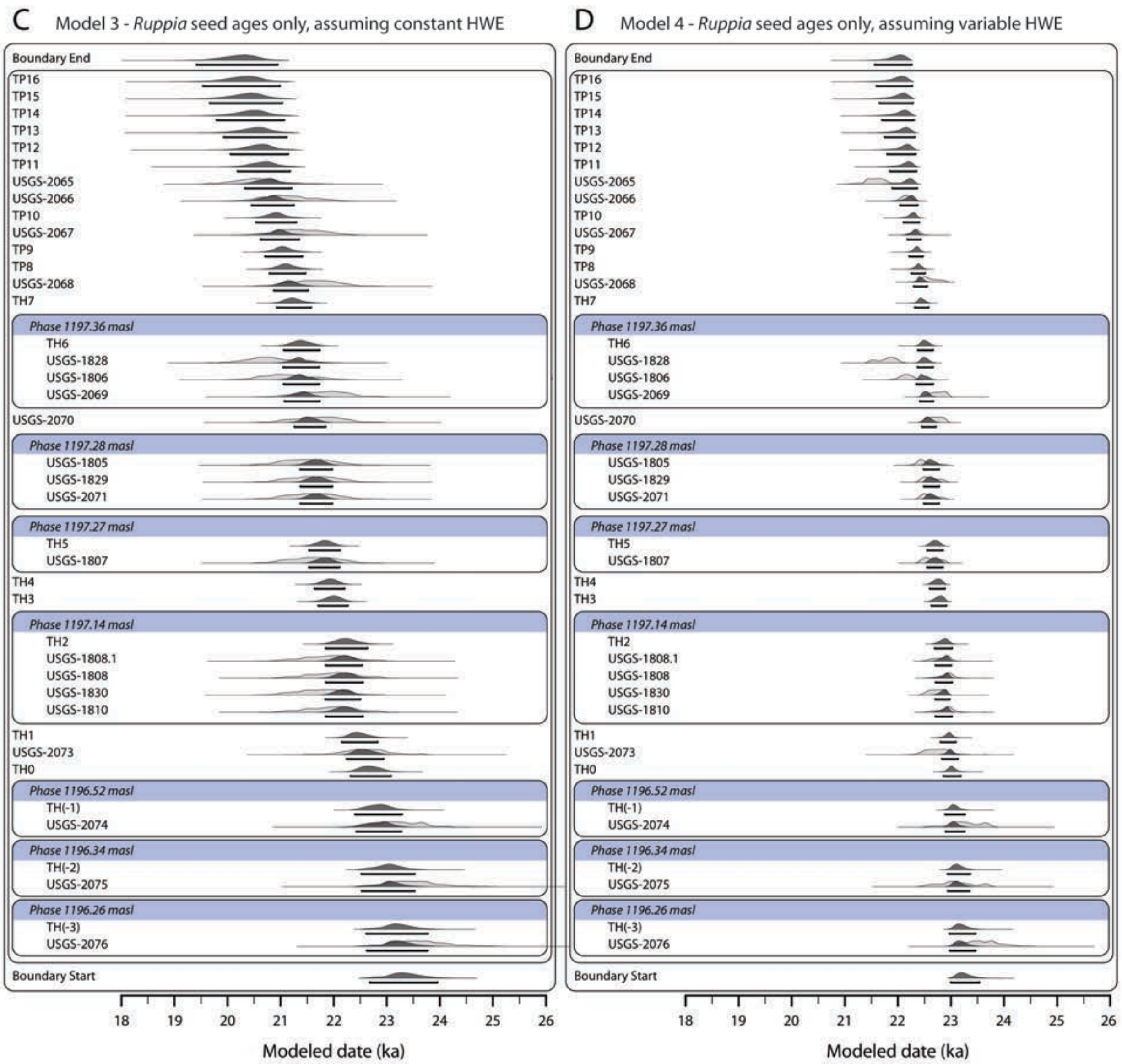

Fig. S18 (cont'd). Modeled ages using the `Sequence` function in OxCal for the track-bearing sequence at WHSA Locality 2 for (**C**) Model 3: *Ruppia* seed ages only, corrected assuming a constant hard-water effect (HWE), and (**D**) Model 4: *Ruppia* seed ages only, corrected assuming a variable hard-water effect. All figures were generated using OxCal v.4.4.4 (*57*) and the IntCal20 calibration curve (*58*); accessed online 20 July 2025. Samples USGS-1803 and USGS-1804 were identified as outliers at the 95% confidence level, so were excluded from the analysis.

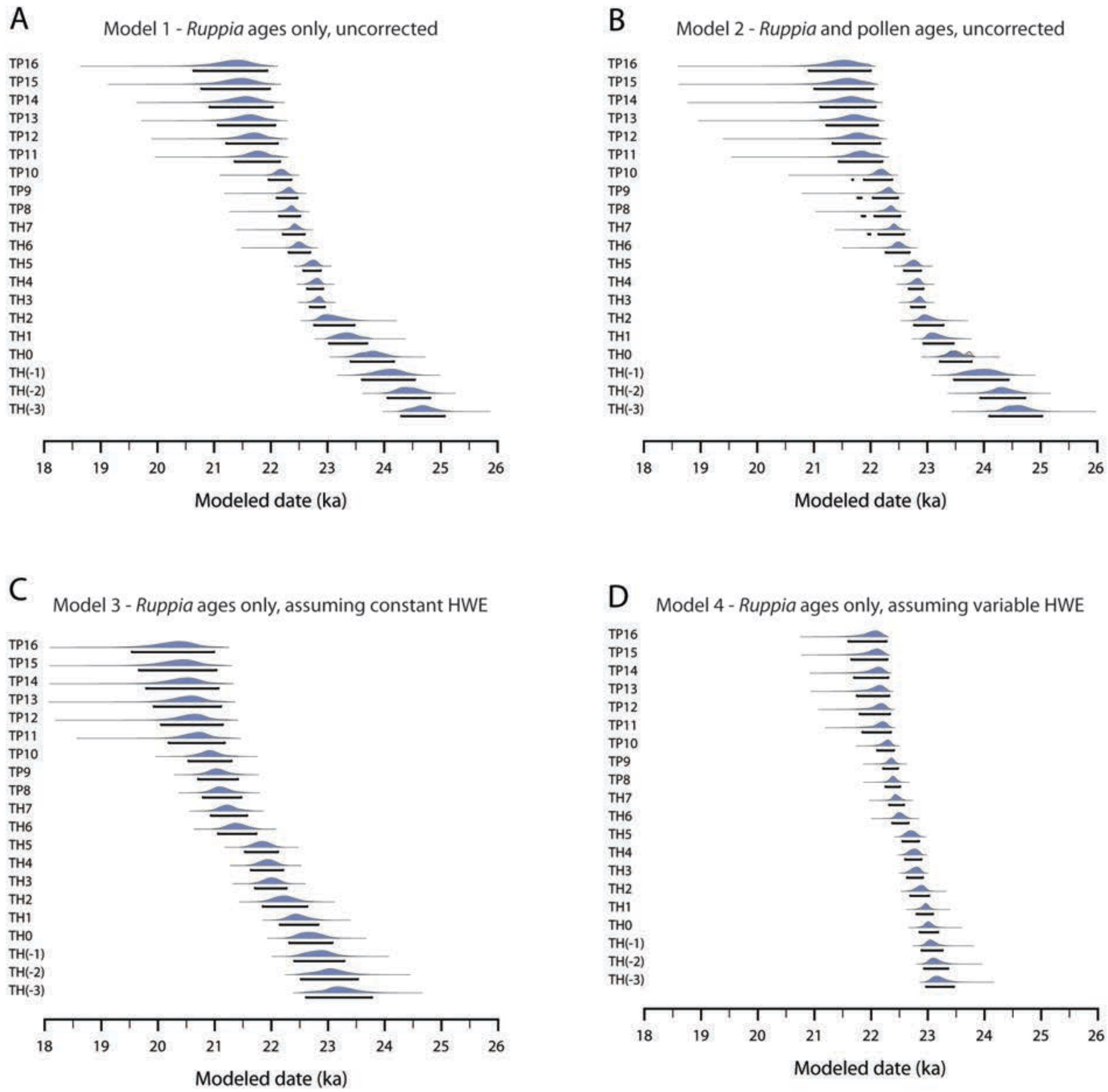

Fig. S19. Modeled ages using the `Sequence` function in OxCal of only the track horizons and track packages [TH(-3) through TP16] at WHSA Locality 2 for (**A**) Model 1: *Ruppia* seed ages only, uncorrected, (**B**) Model 2: *Ruppia* seed ages and pollen ages, both uncorrected, (**C**) Model 3: *Ruppia* seed ages only, corrected assuming a constant hard-water effect (HWE), and (**D**) Model 4: *Ruppia* seed ages only, corrected assuming a variable hard-water effect. All figures were generated using OxCal v.4.4.4 (*57*) and the IntCal20 calibration curve (*58*); accessed online 20 July 2025. Samples USGS-1803 and USGS-1804 were identified as outliers at the 95% confidence level, so were excluded from the analysis.

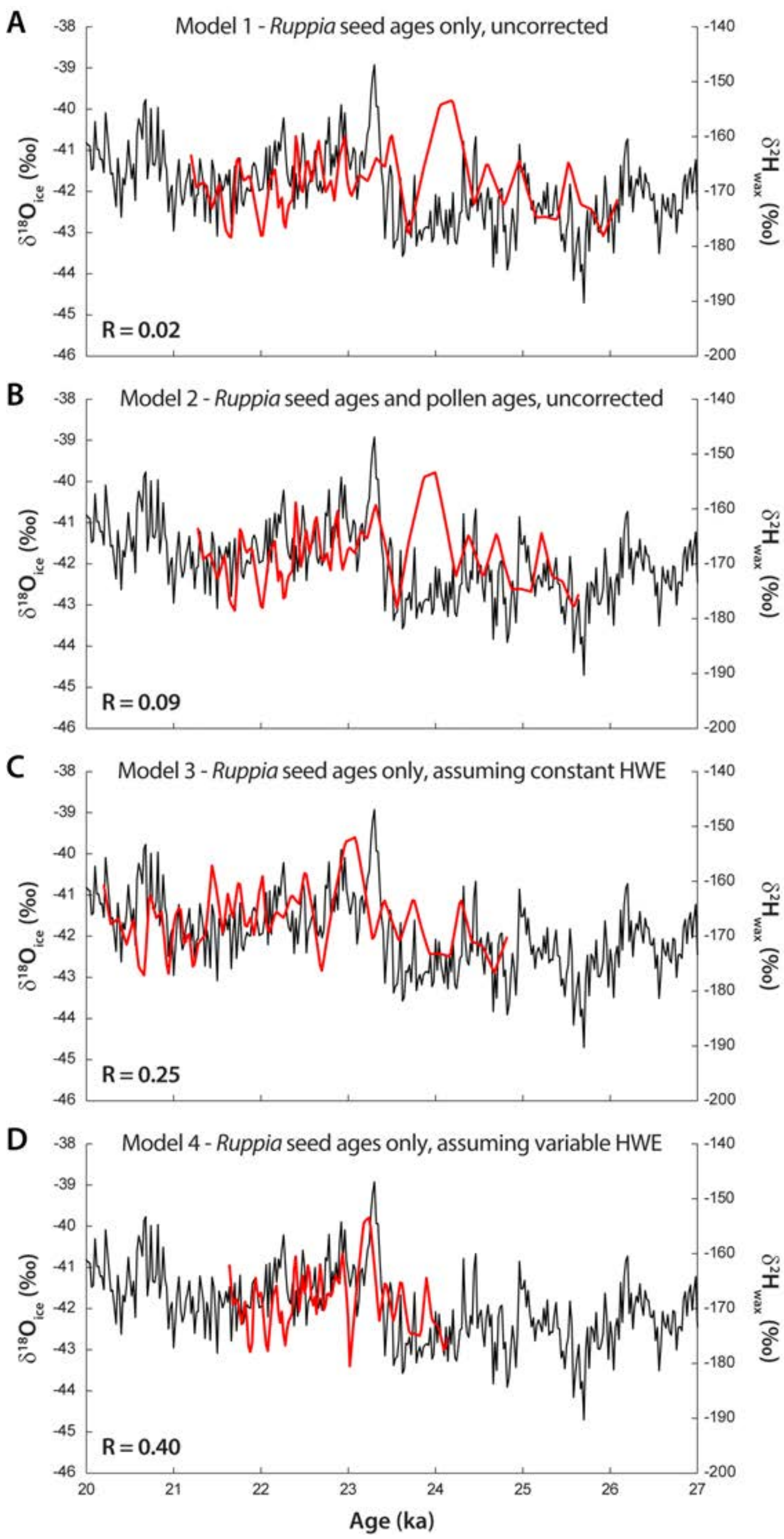

Fig. S20. Isotopic composition (δ^2H) of leaf waxes from samples collected from the WHSA Locality 2 sedimentary sequence (red lines) compared to average oxygen isotopic values from the North Greenland Ice Core Project (NGRIP) and Greenland Ice Sheet Project 2 (GISP2) records (black lines). (**A**) Model 1: *Ruppia* seed ages only, uncorrected, (**B**) Model 2: *Ruppia* seed ages and pollen ages, both uncorrected, (**C**) Model 3: *Ruppia* seed ages only, corrected assuming a constant hard-water effect (HWE), and (**D**) Model 4: *Ruppia* seed ages only, corrected assuming a variable hard-water effect.

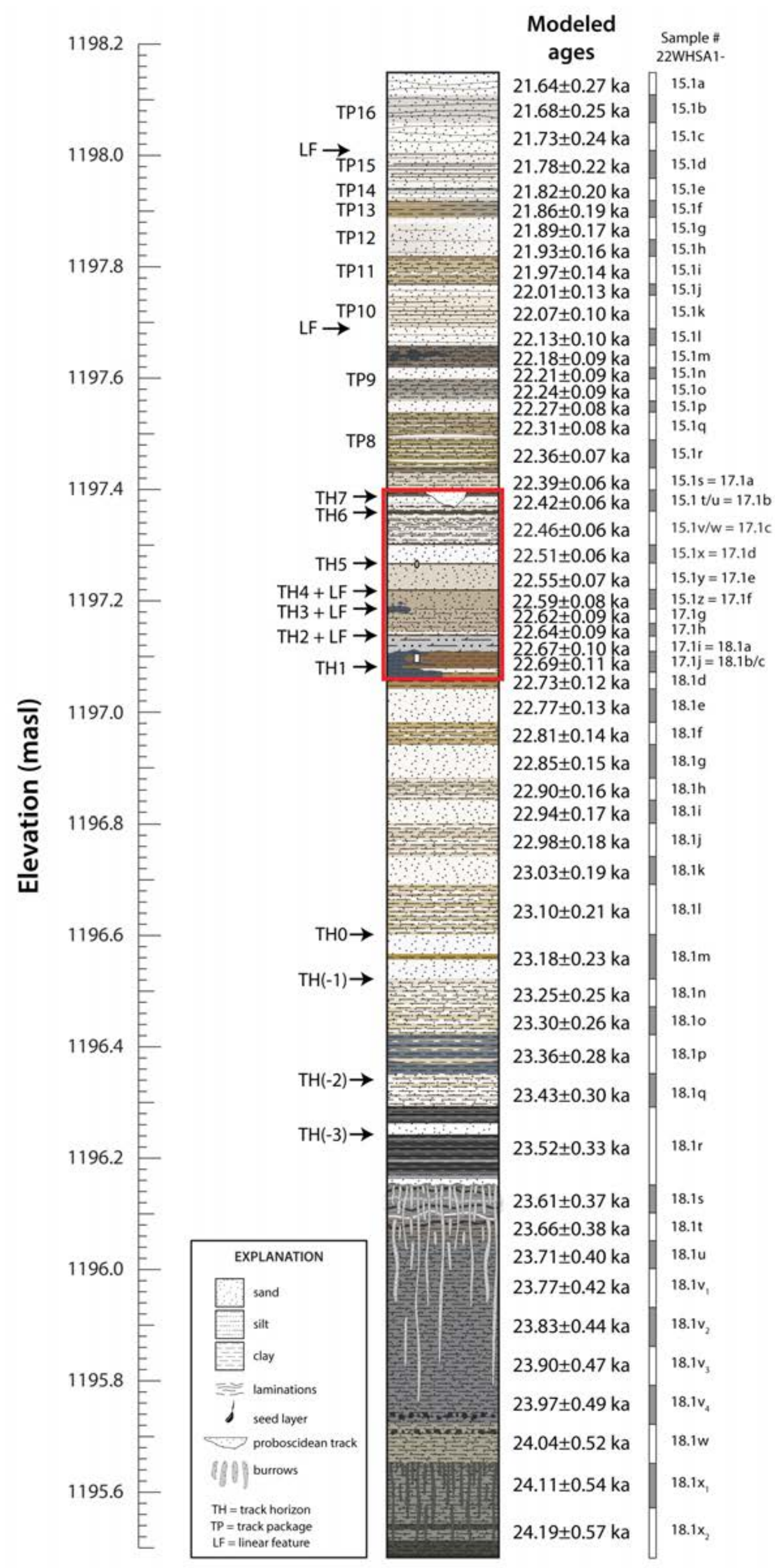

Fig. S21. Composite stratigraphic section showing the locations of proxy samples, the corresponding ages of Model 4, and elevation. The red box depicts the extent of the stratigraphy at WHSA Locality 2 containing track horizons reported previously (*1*).

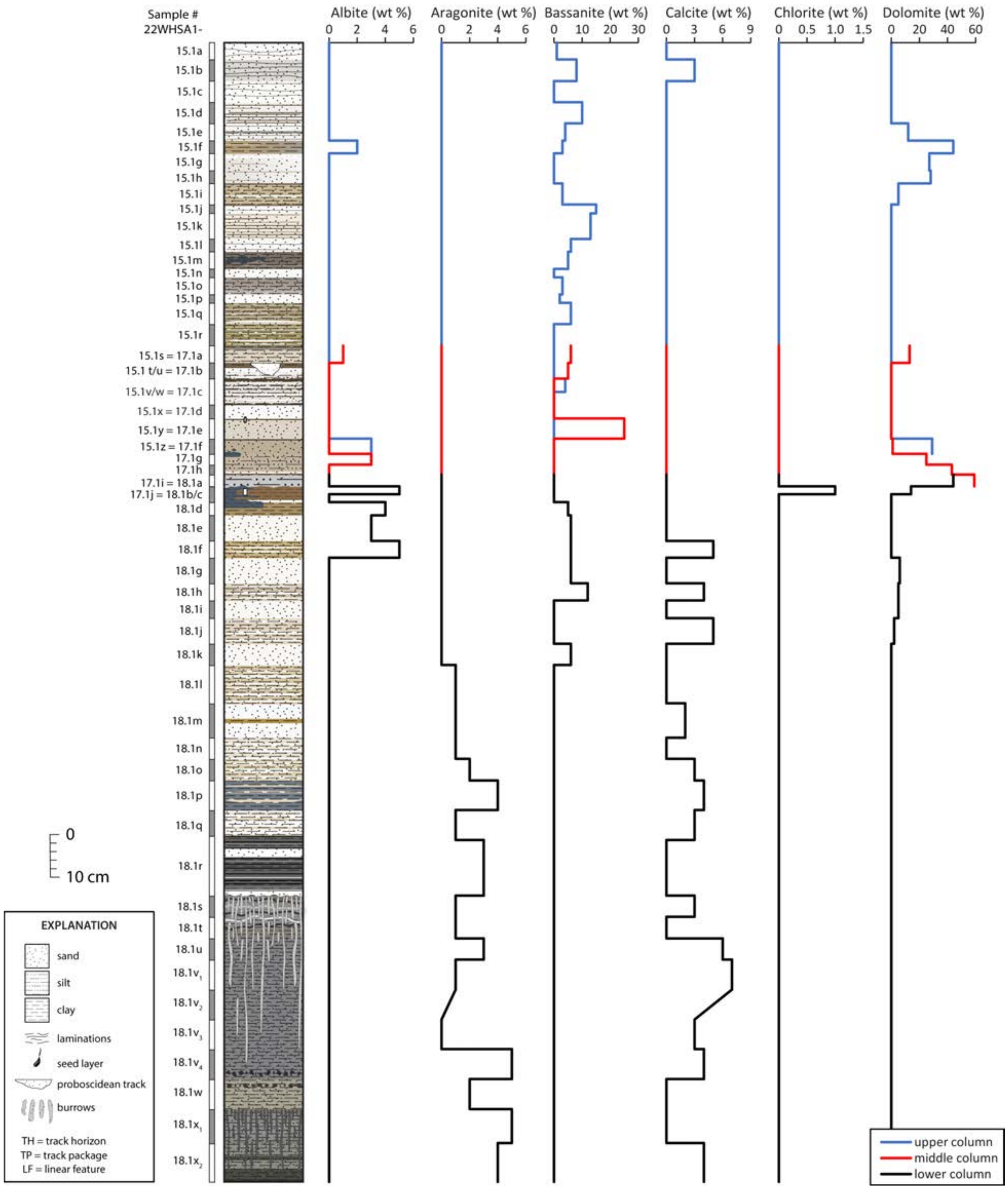

Fig. S22. Composite stratigraphic section, samples, and X-ray diffraction data for albite, aragonite, bassanite, calcite, chlorite, and dolomite.

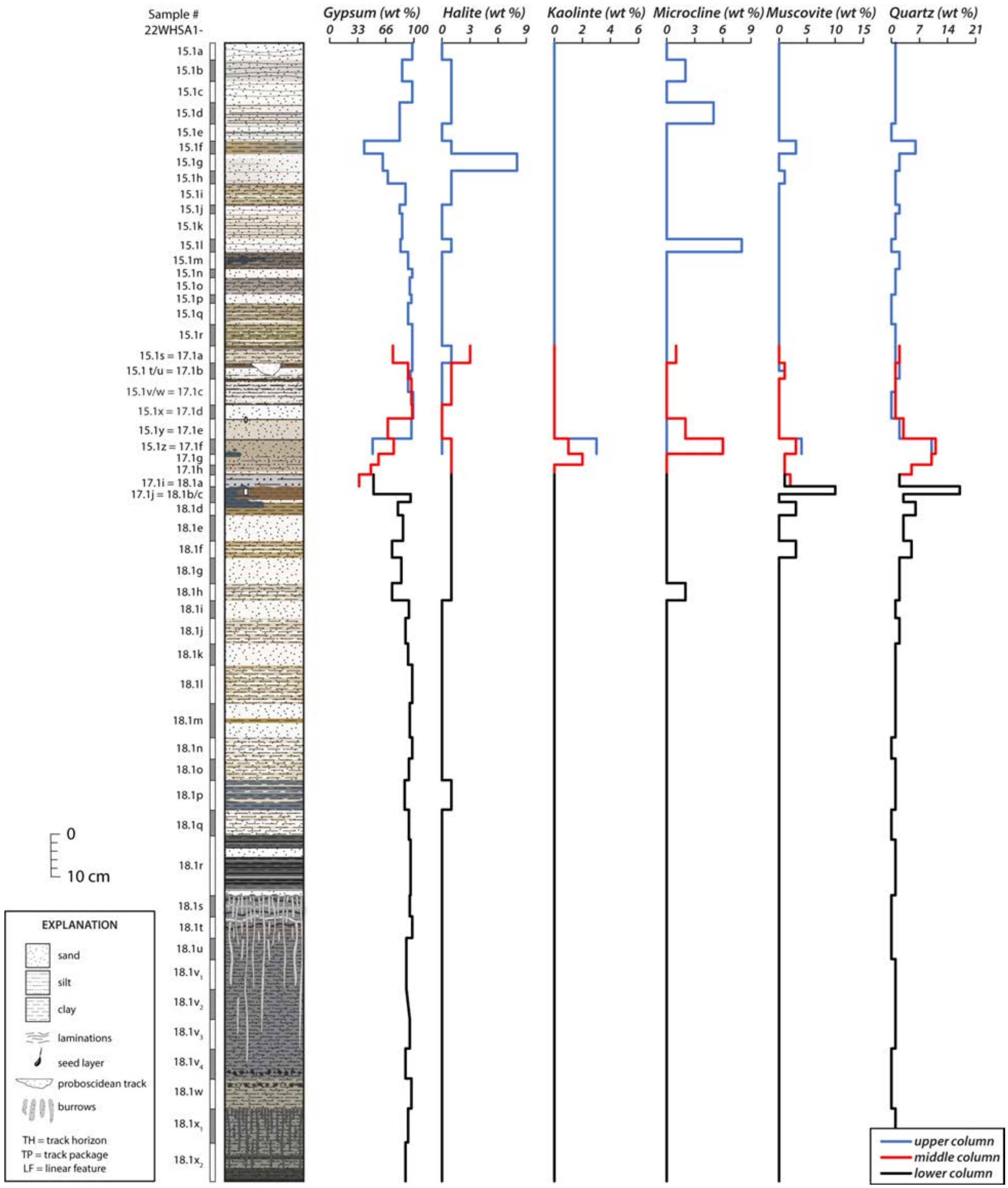

Fig. S22 (cont'd). Composite stratigraphic section, samples, and X-ray diffraction data for gypsum, halite, kaolinite, microcline, muscovite, and quartz.

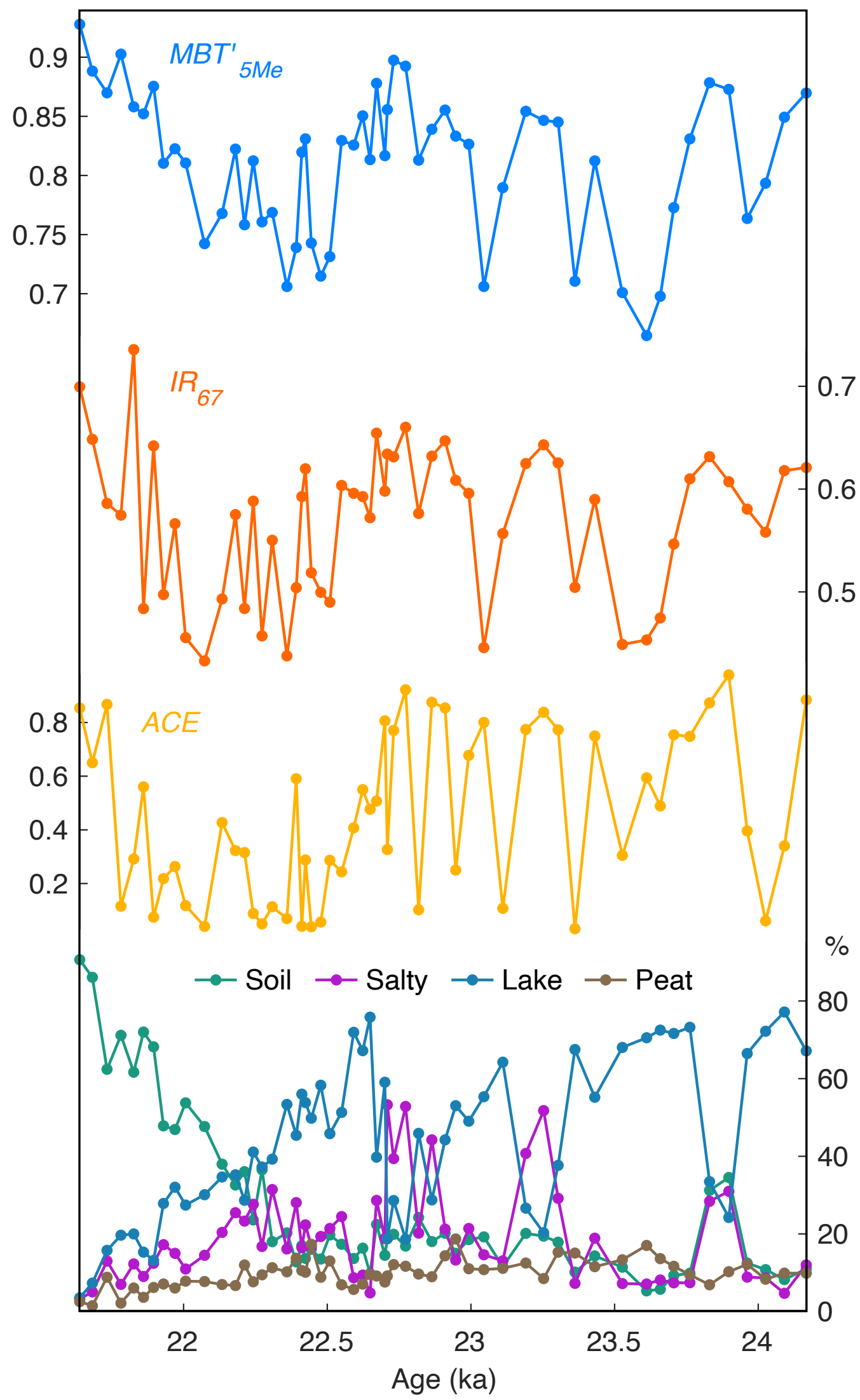

Fig. S23. GDGT indices measured at WHSA Locality 2: MBT'$_{5Me}$ (affected by temperature and salinity), IR_{67} (salinity sensitive), ACE (salinity sensitive), and BIGMaC classification probabilities, including Soil-Type, Salty (Marine)-Type, Lake-Type, and Peat-Type.

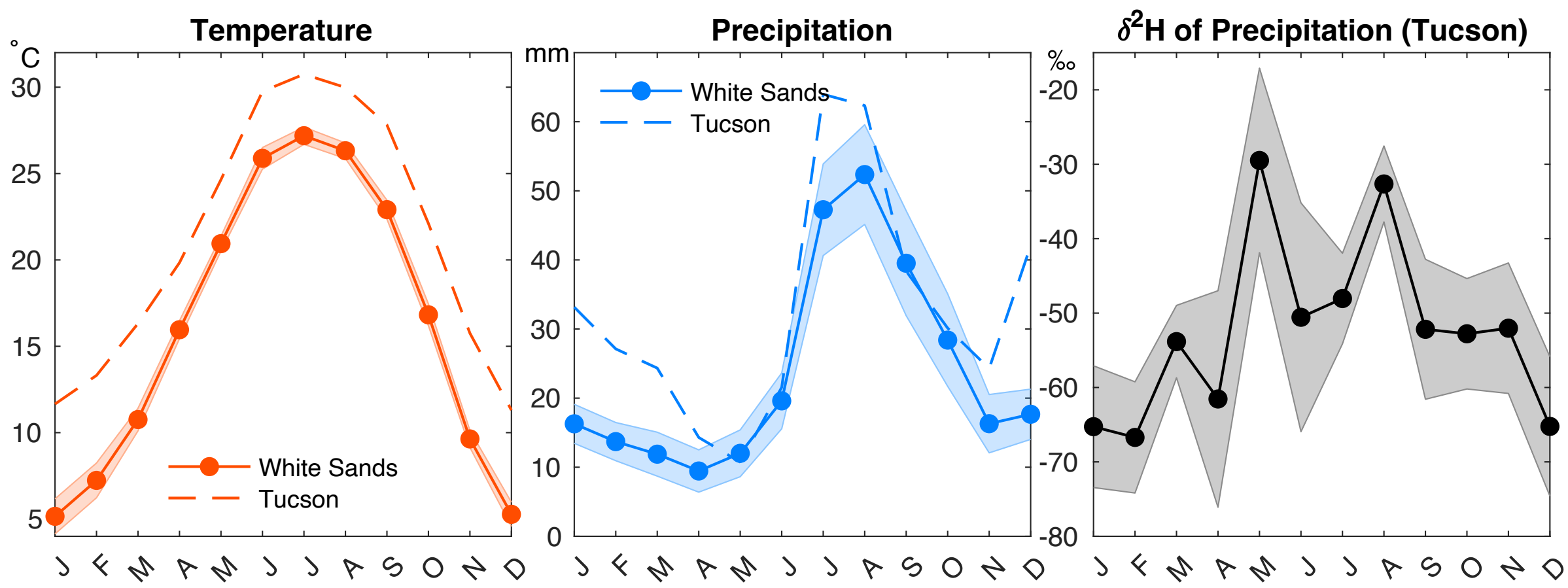

Fig. S24. Temperature and precipitation climatology for the White Sands area and Tucson, AZ, and δ^2H of precipitation from the long-term monitoring station in Tucson (*72*). White Sands temperature data are taken from Holloman Air Force Base (WMO station code 74732.1) and White Sands precipitation data are taken from Alamogordo, NM (WMO station code 74732.10). Temperature, precipitation, and δ^2H of precipitation from Tucson are taken from the GNIP station (WMO code 7227401).

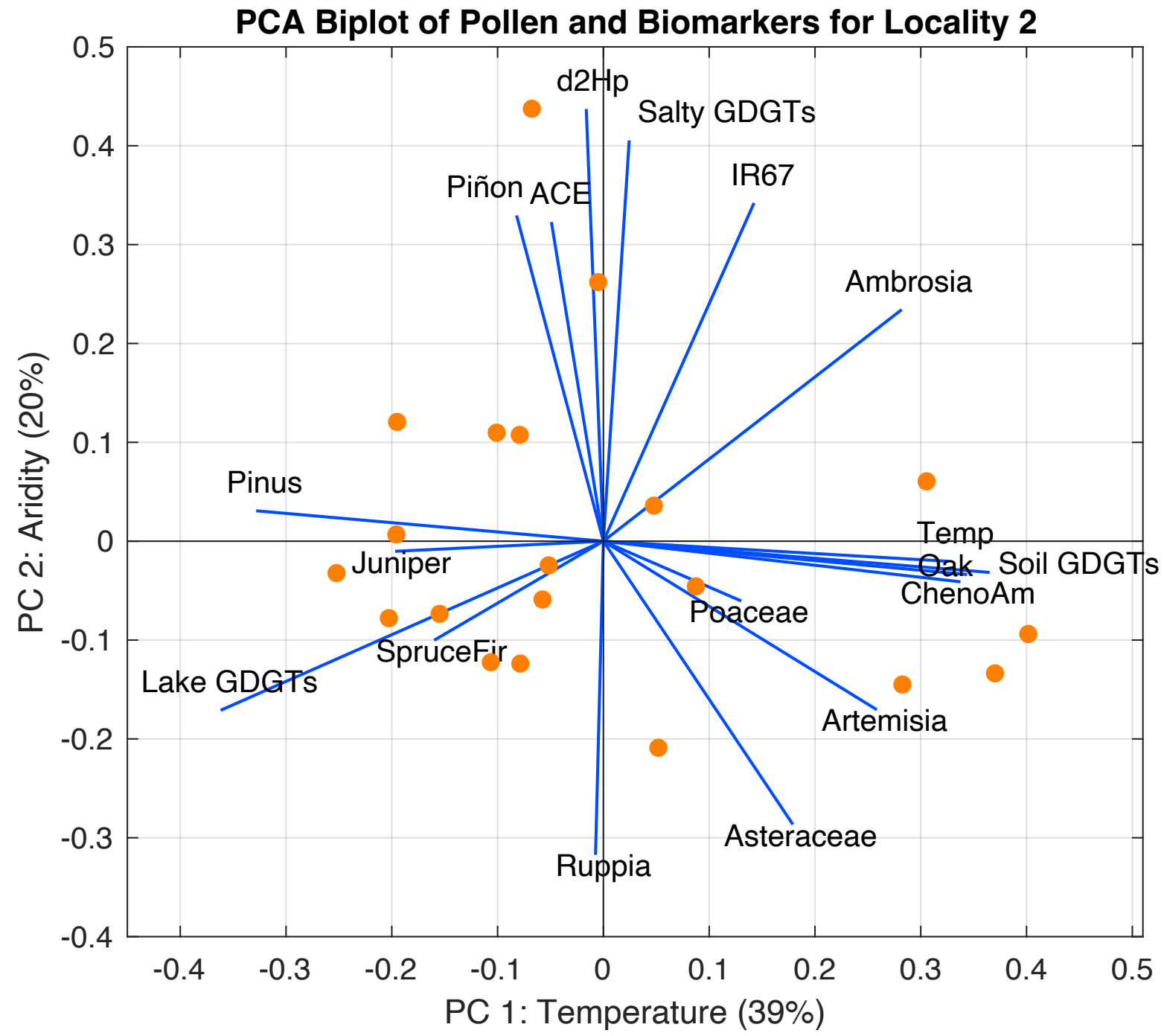

Fig. S25. Principal Components Analysis (PCA) biplot showing the loadings (blue lines) and scores (orange dots) from PCA analysis of the combined pollen and biomarker dataset. The leading two PCs have been varimax-rotated for interpretability. The amount of variance accounted for by each PC is shown in parentheses. The temporal evolution of these PCs is plotted in Figure 4.

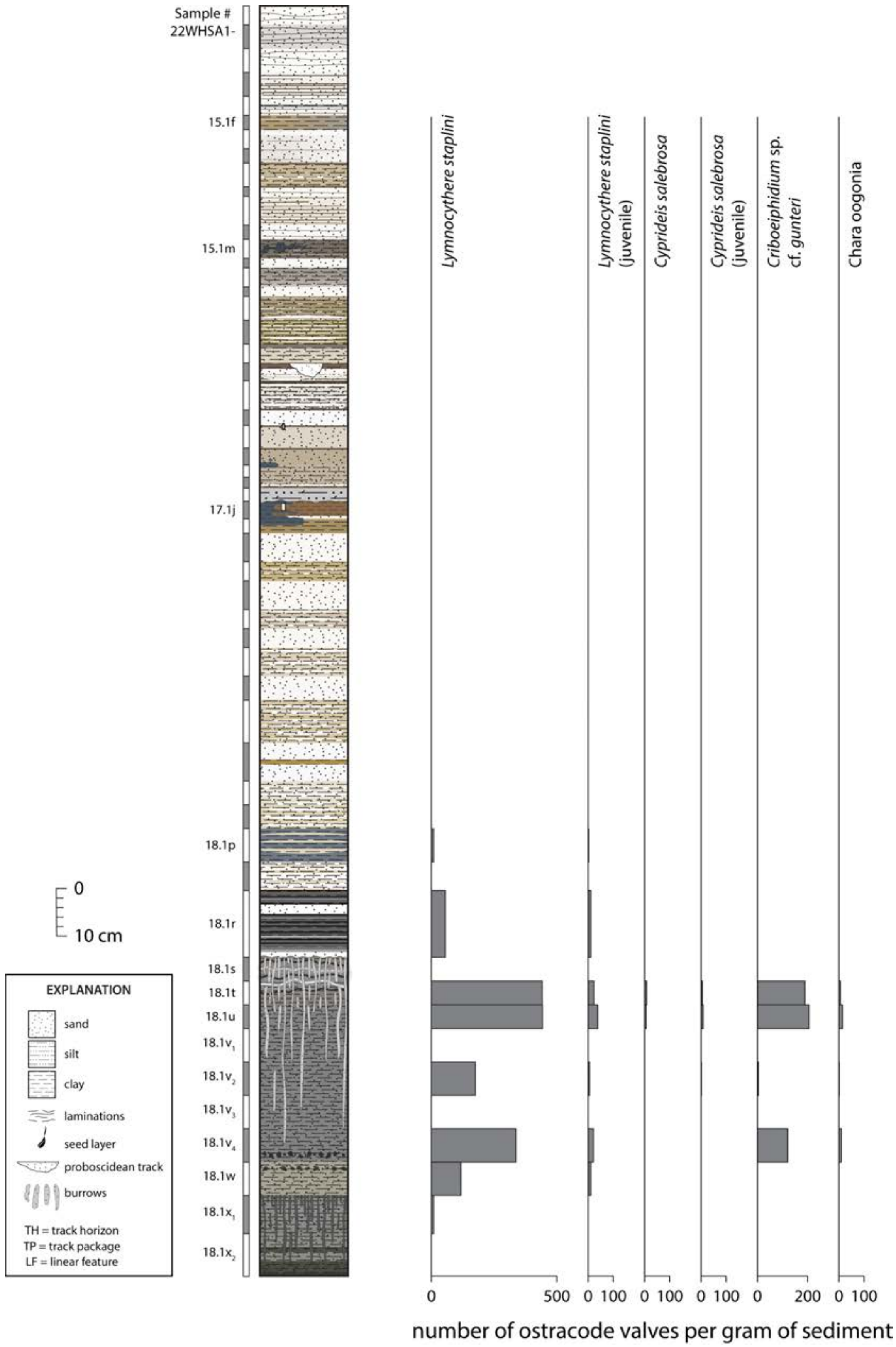

Fig. S26. Composite stratigraphic section, microfaunal samples, and count data for ostracodes, foraminifera, and algae.

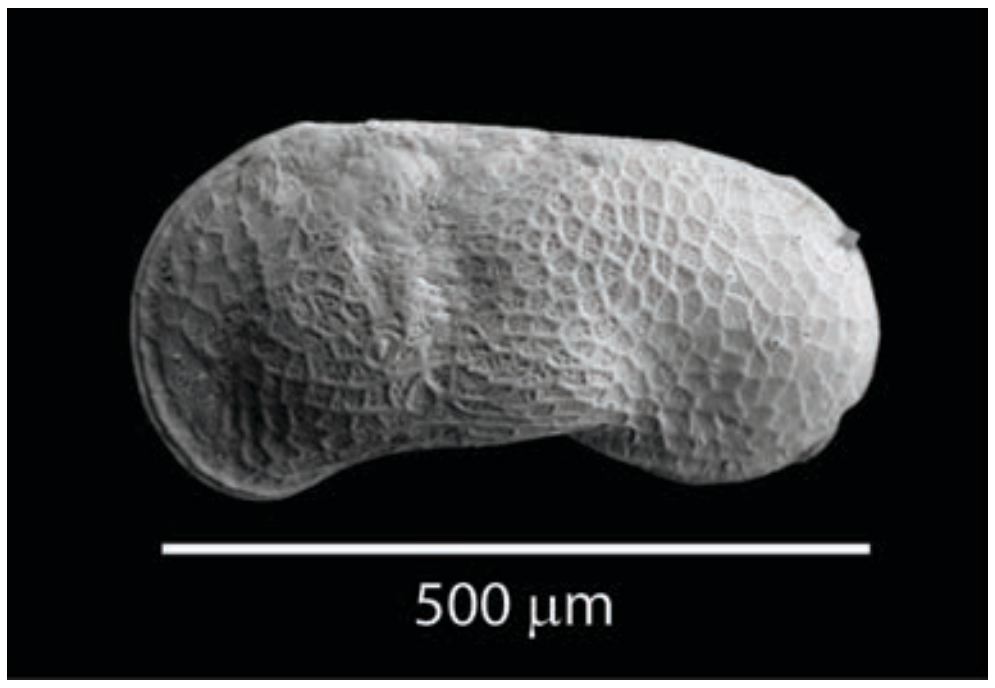

Fig. S27. Scanning electron microscope (SEM) image of *Limnocythere staplini* from WHSA Locality 2.

Table S1: Details of the track-bearing horizons at WHSA Locality 2. All surfaces discussed here are contiguous with the main trench.

TH	Track-makers	Area (m^2)	Human Tracks	R	MNI	T/m^2	I/m^2	Description	Date	Figures
TP16	Human<br>Proboscidea<br>Folivora (ground sloth)<br>Carnivora (canid, ursid?)	1.00	2	32	1	2	1	The top surface of the trench extends for several meters as a desiccated (+cracks) layer with shallow human prints visible in low light and degrading rapidly due to natural erosion. Adjacent to the trench, features interpreted as possible bear tracks have been identified but are unconfirmed due to a lack of unambiguous morphology and potential over-excavation due to poor textural/color contrasts. Sloth tracks have also been identified on this surface.	2022	
TP15	Human	0.80	14	134	5	18	5	This exposure consists of an irregular surface approximately 50 cm wide and 1 m long cut into the south face of the main east-west trench. At least three track surfaces are visible, superimposed on one another. The uppermost track probably lies on TP16 and consists of an indurated track forming positive relief (i.e., dolomite infill).	2022	S7
TP14	Human	0.69	3	52	2	4	2	A reentrant was cut into the north face of the main trench. Three tracks were identified, one of which may show features consistent with being shod (i.e., possible woven sandal impression), but is a single example and remains unconfirmed.	2022	S8
TP13	Human	1.00	5	-	1	5	1	This is an indurated block with an irregular top surface with at least one poor quality track.	2022	
TP12	Human	0.96	11	72	3	11	2	These surfaces were exposed on the southern shoulder of the trench and form an irregular bench which cuts through several footprint layers. The indurated nature of the surface makes separation of individual layers difficult.	2022	S9
TP11-TP10	Human	0.33	4	11	1	12	2	Two closely spaced track surfaces.	2022	S3E, S10
TP 9	Human	0.35	4	16	1	11	1	Surface with tracks and linear features (Bennett et al., 2025). Tracks on top of infilled linear feature.	2022	S11
TP8	Human	0.58	8	145	5	14	7	Bench on south side of the trench. Heavily trampled, lots of surface relief, but tracks have poor individual anatomical definition.	2020<br>2022	
TH7	Human<br>Proboscidea	0.20	1	-	1	5	1	Single track in base of the trench excavated in 2020 (Bennett et al., 2021).	2020	
TH6	Human	0.25	1	-	1	4	1	Single track in base of the trench excavated in 2020 (Bennett et al., 2021).	2020	
TH5	Human	0.34	3	18	1	9	1	Limited number of tracks in base of trench and on south shoulder excavated in 2020 (Bennett et al., 2021).	2020	

TH4	Human	6.70	37	124	4	6	1	Tracks on south shoulder excavated in 2020 (Bennett et al., 2021).	2020	
TH3	Human<br>Proboscidea<br>Carnivora (canid)	1.35	19	138	5	14	3	Range of human and proboscidean tracks, including linear features described by Bennett et al. (2025). Extensively described by Bennett et al. (2021, 2025). Included here are only those tracks adjacent to the main trench.	2020<br>2022	S3C,<br>S12
TH2	Human<br>Proboscidea<br>Carnivora (canid)<br>Aves	3.82	45	180	6	12	1	Range of human and proboscidean tracks, including linear features described by Bennett et al. (2025) as possible travois. Tracks both in positive and negative relief with inverted dolomite fills. Extensively described by Bennett et al. (2021, 2025). Included here are only those tracks adjacent to the main trench.	2020<br>2022	S3C,<br>S12
TH1	Human	0.32	5	32	1	16	3	Range of human tracks located in a north-south trending extension to the main trench. Described and documented in Bennett et al. (2021).	2020	
TH0	Human	0.33	4	11	1	12	2	Low quality tracks. Trampled.	2022	S13
TH(-1)	Human	0.20	1	-	1	5	1	Low quality tracks. Trampled.	2022	
TH(-2)	Human	-	-	-	-	-	-	Low quality tracks. Trample with a potential handprint (?). This layer was removed after documentation so that the bottom of the trench could be exposed.	2022	
TH(-3)	Human	0.26	7	72	4	27	13	Low quality tracks. Heavily trampled. Potential example of superimposed child footprints.	2022	S14

TP = track package
TH = track horizon
R = range of sizes on a tracked surface of similar age
MNI = minimum number of individuals
T/m^2 = tracks per square meter
I/m^2 = individuals per square meter

Table S2

Biometric inferences for sub-populations of human footprints at WHSA Locality 2.

Mean foot size (mm)	Standard error (1σ)	N	Stature (m)	Age (years)
127.1	3.5	8	0.85	<4
180.8	1.8	72	1.21	6.3±0.1
218.8	0.3	12	1.46	11.8±0.2
244.9	2.4	60	1.63	13.8±0.2 (male)<br>14.6±0.2 (female)

Note: Stature is based on foot length being 15% of stature and Age is based on data compiled by (51).

Table S3

Summary of information and carbon-14 data for samples from the Ziegler Reservoir fossil site.

Sample #	AMS #	**$F^{14}C$**
ZR11	USGS-2237	0.0030 ± 0.0002
ZR12	USGS-2294	0.0053 ± 0.0004
ZR13b	USGS-2296	0.0001 ± 0.0003
ZR14	USGS-2297	0.0032 ± 0.0004
ZR15	USGS-2298	0.0000 ± 0.0003
	**Average**	**0.0023 ± 0.0023**[1]

[1] The uncertainty of the average $F^{14}C$ value was calculated as the standard deviation and is reported at the 68% (1σ) confidence level.

Table S4

Summary of information, carbon-14 ages, and calibrated ages for samples collected from the extended trench at WHSA Locality 2.

Sample #	AMS #	Elevation (masl)[1]	Material dated	^{14}C age (ka)	Calibrated age range (ka)[2]	Mean calibrated age (ka)[3]	Source[4]
Track package 16		1198.08					
Track package 15		1197.98					
Track package 14		1197.94					
Track package 13		1197.90					
Track package 12		1197.85					
Track package 11		1197.79					
22WHSA1-15.1jj	USGS-2065	1197.76	*Ruppia cirrhosa*	17.76 ± 0.08	21.91 - 21.25	21.58 ± 0.33	3
22WHSA1-15.1kk	USGS-2066	1197.72	*Ruppia cirrhosa*	18.19 ± 0.08	22.34 - 21.97	22.15 ± 0.18	3
Track package 10		1197.71					
22WHSA1-15.1mm	USGS-2067	1197.64	*Ruppia cirrhosa*	18.41 ± 0.09	22.49 - 22.15	22.32 ± 0.17	3
Track package 9		1197.60					
Track package 8		1197.49					
22WHSA1-15.1rr	USGS-2068	1197.46	*Ruppia cirrhosa*	18.65 ± 0.09	22.89 - 22.39	22.64 ± 0.25	3
Track horizon 7		1197.39					
Track horizon 6		1197.36					
20WHSA1-18.1a	USGS-1803	1197.36	*Ruppia cirrhosa*	17.47 ± 0.09	21.38 - 20.88	21.13 ± 0.25[6]	1
20WHSA1-18.1a[5]	USGS-1828	1197.36	*Ruppia cirrhosa*	17.93 ± 0.09	22.06 - 21.44	21.75 ± 0.31	1
20WHSA1-18.1e	USGS-1806	1197.36	*Ruppia cirrhosa*	18.20 ± 0.11	22.39 - 21.92	22.15 ± 0.23	1
22WHSA1-15.1vv1	USGS-2069	1197.36	*Ruppia cirrhosa*	18.86 ± 0.09	22.98 - 22.52	22.75 ± 0.23	3
20WHSA1-18.1aa	CAMS-190357A	1197.36	pollen	19.25 ± 1.05	25.92 - 20.95	23.4 ± 2.5	2
20WHSA1-18.1b	USGS-1804	1197.30	*Ruppia cirrhosa*	17.83 ± 0.09	21.98 - 21.38	21.68 ± 0.30[6]	1
22WHSA1-15.1vv2	USGS-2070	1197.30	*Ruppia cirrhosa*	18.76 ± 0.09	22.93 - 22.46	22.69 ± 0.24	3
20WHSA1-18.1d	USGS-1805	1197.28	*Ruppia cirrhosa*	18.55 ± 0.10	22.83 - 22.28	22.55 ± 0.28	1
20WHSA1-18.1d[5]	USGS-1829	1197.28	*Ruppia cirrhosa*	18.66 ± 0.10	22.90 - 22.39	22.64 ± 0.26	1
22WHSA1-15.1xx	USGS-2071	1197.28	*Ruppia cirrhosa*	18.64 ± 0.09	22.88 - 22.38	22.63 ± 0.25	3
20WHSA1-18.1dd	CAMS-190358A	1197.28	pollen	18.60 ± 0.96	25.13 - 20.41	22.8 ± 2.4	2
Track horizon 5		1197.27					
20WHSA1-18.1f	USGS-1807	1197.27	*Ruppia cirrhosa*	18.68 ± 0.11	22.91 - 22.39	22.65 ± 0.26	1
20WHSA1-18.1ff	CAMS-190359A	1197.27	pollen	18.50 ± 0.95	24.99 - 20.30	22.6 ± 2.3	2
Track horizon 4		1197.22					
Track horizon 3		1197.19					
Track horizon 2		1197.14					
20WHSA1-18.2a[5]	USGS-1808.1	1197.14	*Ruppia cirrhosa*	18.95 ± 0.11	23.06 - 22.53	22.80 ± 0.26	1
20WHSA1-18.2a	USGS-1808	1197.14	*Ruppia cirrhosa*	19.02 ± 0.11	23.17 - 22.57	22.87 ± 0.30	1
20WHSA1-18.3a[5]	USGS-1830	1197.14	*Ruppia cirrhosa*	18.81 ± 0.10	22.96 - 22.48	22.72 ± 0.24	1
20WHSA1-18.3a	USGS-1810	1197.14	*Ruppia cirrhosa*	19.01 ± 0.12	23.18 - 22.55	22.86 ± 0.32	1
Track horizon 1		1197.08					
22WHSA1-18.1hh	USGS-2330	1196.86	pollen	18.82 ± 0.18	23.04 - 22.39	22.71 ± 0.33	3
22WHSA1-18.1k	USGS-2073	1196.74	*Ruppia cirrhosa*	19.57 ± 0.10	23.81 - 23.26	23.54 ± 0.27	3
Track horizon 0		1196.60					
Track horizon (-1)		1196.52					
22WHSA1-18.1n	USGS-2074	1196.52	*Ruppia cirrhosa*	20.16 ± 0.10	24.53 - 23.89	24.21 ± 0.32	3
22WHSA1-18.1nn	USGS-2331	1196.52	pollen	18.83 ± 0.18	23.05 - 22.39	22.72 ± 0.33	3
Track horizon (-2)		1196.34					
22WHSA1-18.1q	USGS-2075	1196.34	*Ruppia cirrhosa*	20.49 ± 0.11	25.00 - 24.27	24.63 ± 0.37	3
Track horizon (-3)		1196.26					
22WHSA1-18.1r	USGS-2076	1196.26	*Ruppia cirrhosa*	20.64 ± 0.11	25.21 - 24.53	24.87 ± 0.34	3
22WHSA1-18.1rr	USGS-2332	1196.26	pollen	19.52 ± 0.20	23.87 - 23.02	23.44 ± 0.43	3

Uncertainties for the calibrated ages are given at the 2 σ (95%) confidence level; uncertainties for the ^{14}C ages are given at 1 σ (68%).

[1] Track packages vary in thickness, so the values given represent the average elevation of each package. In contrast, track horizons are isochronous surfaces so are accurate at the centimeter scale.

[2] Calibrated age ranges were calculated using OxCal v.4.4.4, IntCal20.14C dataset; limit 55.0 calendar ka B.P.

[3] Uncertainties for the mean calibrated ages represent the difference between the mean age and the upper/lower limits of the age range.

[4] Source of data: 1= Bennett et al., 2021; 2 = Pigati et al., 2023; 3 = this study.

[5] Replicate sample.

[6] Outlier as defined by the `Deposition` model in OxCal4.4.4.

Table S5
Calculation of the average HWE value for *Ruppia* seeds from the extended trench at WHSA Locality 2.

Sample #	AMS #	Elevation (masl)	Pollen ^{14}C age (ka)	Pollen $F^{14}C$	*Ruppia* ^{14}C age (ka)	*Ruppia* $F^{14}C$	HWE correction factor[2]
22WHSA1-18.1hh[1]	USGS-2330	1196.86	18.82 ± 0.18	0.0961 ± 0.0022	19.33 ± 0.21	0.0901 ± 0.0023	0.0059 ± 0.0032
22WHSA1-18.1n/nn	USGS-2074/2331	1196.52	18.83 ± 0.18	0.0959 ± 0.0021	20.16 ± 0.10	0.0813 ± 0.0010	0.0146 ± 0.0024
22WHSA1-18.1r/rr	USGS-2076/2332	1196.26	19.52 ± 0.20	0.0880 ± 0.0022	20.64 ± 0.11	0.0766 ± 0.0010	0.0115 ± 0.0024
						**Average =**	**0.0107 ± 0.0044[3]**

Uncertainties are all given at the 68% (1σ) confidence level.

[1] The modeled ^{14}C age of *Ruppia* seeds for sample 22WHSA1-18.1hh was determined using the entire suite of *Ruppia* seed ^{14}C ages, the elevation of sample 22WHSA1-18.1h (USGS-2330), the `Deposition` function in OxCal v.4.4.4, and the IntCal20 calibration curve; accessed online 09 July 2025.
[2] HWE correction factors for each sample were determined by subtracting the *Ruppia* $F^{14}C$ value from the corresponding pollen $F^{14}C$ value.
[3] The uncertainty of the average HWE correction factor was calculated as the standard deviation and is reported at the 68% (1σ) confidence level.

Table S6
Summary of information used for correcting carbon-14 ages of *Ruppia* seeds from the extended trench at WHSA Locality 2 assuming a constant hard-water effect.

Sample #	AMS #	Elevation (masl)	Cal age (ka)	^{14}C age (ka)	$F^{14}C$	HWE correction factor[2]	Corrected $F^{14}C$[3]	Corrected ^{14}C age (ka)[4]	Corrected calibrated age range (ka)[5]	Corrected mean calibrated age (ka)[6]	HWE offset (^{14}C ka)
22WHSA1-15.1jj	USGS-2065	1197.76	21.58 ± 0.33	17.76 ± 0.08	0.1096 ± 0.0011	0.0107 ± 0.0044	0.1203 ± 0.0045	17.01 ± 0.31	21.42 - 19.64	20.53 ± 0.89	0.75
22WHSA1-15.1kk	USGS-2066	1197.72	22.15 ± 0.18	18.19 ± 0.08	0.1039 ± 0.0010	0.0107 ± 0.0044	0.1146 ± 0.0045	17.40 ± 0.32	22.01 - 20.33	21.17 ± 0.84	0.79
22WHSA1-15.1mm	USGS-2067	1197.64	22.32 ± 0.17	18.41 ± 0.09	0.1011 ± 0.0011	0.0107 ± 0.0044	0.1118 ± 0.0045	17.60 ± 0.33	22.18 - 20.53	21.36 ± 0.83	0.81
22WHSA1-15.1rr	USGS-2068	1197.46	22.64 ± 0.25	18.65 ± 0.09	0.0981 ± 0.0011	0.0107 ± 0.0044	0.1088 ± 0.0045	17.82 ± 0.34	22.42 - 20.79	21.60 ± 0.82	0.83
20WHSA1-18.1a	USGS-1803	1197.36	21.13 ± 0.25	17.47 ± 0.09	0.1136 ± 0.0013	0.0107 ± 0.0044	0.1243 ± 0.0046	16.75 ± 0.30	20.94 - 19.52	20.23 ± 0.71	0.72
20WHSA1-18.1a[1]	USGS-1828	1197.36	21.75 ± 0.31	17.93 ± 0.09	0.1073 ± 0.0012	0.0107 ± 0.0044	0.1180 ± 0.0046	17.17 ± 0.32	21.76 - 19.97	20.86 ± 0.90	0.76
20WHSA1-18.1e	USGS-1806	1197.36	22.15 ± 0.23	18.20 ± 0.11	0.1038 ± 0.0014	0.0107 ± 0.0044	0.1145 ± 0.0046	17.41 ± 0.33	22.04 - 20.32	21.18 ± 0.86	0.79
22WHSA1-15.1vv1	USGS-2069	1197.36	22.75 ± 0.23	18.86 ± 0.09	0.0956 ± 0.0011	0.0107 ± 0.0044	0.1063 ± 0.0045	18.01 ± 0.35	22.62 - 20.89	21.76 ± 0.87	0.85
20WHSA1-18.1b	USGS-1804	1197.30	21.68 ± 0.30	17.83 ± 0.09	0.1087 ± 0.0012	0.0107 ± 0.0044	0.1194 ± 0.0046	17.08 ± 0.31	21.69 - 19.85	20.77 ± 0.92	0.75
22WHSA1-15.1vv2	USGS-2070	1197.30	22.69 ± 0.24	18.76 ± 0.09	0.0968 ± 0.0011	0.0107 ± 0.0044	0.1075 ± 0.0045	17.92 ± 0.35	22.51 - 20.84	21.67 ± 0.84	0.84
20WHSA1-18.1d	USGS-1805	1197.28	22.55 ± 0.28	18.55 ± 0.10	0.0993 ± 0.0012	0.0107 ± 0.0044	0.1100 ± 0.0046	17.73 ± 0.34	22.34 - 20.66	21.50 ± 0.84	0.82
20WHSA1-18.1d[1]	USGS-1829	1197.28	22.64 ± 0.26	18.66 ± 0.10	0.0980 ± 0.0012	0.0107 ± 0.0044	0.1087 ± 0.0046	17.83 ± 0.34	22.43 - 20.80	21.61 ± 0.81	0.83
22WHSA1-15.1xx	USGS-2071	1197.28	22.63 ± 0.25	18.64 ± 0.09	0.0982 ± 0.0011	0.0107 ± 0.0044	0.1089 ± 0.0045	17.81 ± 0.34	22.41 - 20.78	21.60 ± 0.82	0.83
20WHSA1-18.1f	USGS-1807	1197.27	22.65 ± 0.26	18.68 ± 0.11	0.0977 ± 0.0013	0.0107 ± 0.0044	0.1084 ± 0.0046	17.85 ± 0.35	22.46 - 20.79	21.62 ± 0.84	0.83
20WHSA1-18.2a[1]	USGS-1808.1	1197.14	22.80 ± 0.26	18.95 ± 0.11	0.0945 ± 0.0013	0.0107 ± 0.0044	0.1052 ± 0.0046	18.09 ± 0.36	22.80 - 20.98	21.89 ± 0.91	0.86
20WHSA1-18.2a	USGS-1808	1197.14	22.87 ± 0.30	19.02 ± 0.11	0.0937 ± 0.0013	0.0107 ± 0.0044	0.1044 ± 0.0046	18.15 ± 0.36	22.89 - 21.07	21.98 ± 0.91	0.87
20WHSA1-18.3a[1]	USGS-1830	1197.14	22.72 ± 0.24	18.81 ± 0.10	0.0962 ± 0.0012	0.0107 ± 0.0044	0.1069 ± 0.0046	17.96 ± 0.35	22.55 - 20.86	21.71 ± 0.85	0.85
20WHSA1-18.3a	USGS-1810	1197.14	22.86 ± 0.32	19.01 ± 0.12	0.0938 ± 0.0014	0.0107 ± 0.0044	0.1045 ± 0.0046	18.14 ± 0.36	22.87 - 21.06	21.97 ± 0.91	0.87
22WHSA1-18.1k	USGS-2073	1196.74	23.54 ± 0.27	19.57 ± 0.10	0.0875 ± 0.0011	0.0107 ± 0.0044	0.0982 ± 0.0045	18.64 ± 0.38	23.73 - 21.75	22.74 ± 0.99	0.93
22WHSA1-18.1n	USGS-2074	1196.52	24.21 ± 0.32	20.16 ± 0.10	0.0813 ± 0.0010	0.0107 ± 0.0044	0.0920 ± 0.0045	19.17 ± 0.40	24.04 - 22.34	23.19 ± 0.85	0.99
22WHSA1-18.1q	USGS-2075	1196.34	24.63 ± 0.37	20.49 ± 0.11	0.0780 ± 0.0011	0.0107 ± 0.0044	0.0887 ± 0.0045	19.46 ± 0.42	24.47 - 22.50	23.48 ± 0.99	1.03
22WHSA1-18.1r	USGS-2076	1196.26	24.87 ± 0.34	20.64 ± 0.11	0.0766 ± 0.0010	0.0107 ± 0.0044	0.0873 ± 0.0045	19.59 ± 0.43	24.69 - 22.59	23.64 ± 1.05	1.05

Uncertainties for the calibrated ages are given at the 2σ (95%) confidence level; uncertainties for the ^{14}C ages and $F^{14}C$ values are given at 1σ (68%).

[1] Replicate samples.
[2] HWE correction factors were determined in Table S5.
[3] Corrected $F^{14}C$ values were determined by adding the HWE correction factor to the measured $F^{14}C$ value for each sample.
[4] Corrected ^{14}C ages were calculated using the corrected $F^{14}C$ values.
[5] Calibrated age ranges were calculated using OxCal v.4.4.4, IntCal20.14C dataset; limit 55.0 calendar ka B.P.
[6] Uncertainties of the mean calibrated ages represent the difference between the mean age and the upper/lower limits of the age range.

Table S7

Calculation of the individual HWE values for *Ruppia* seeds from the extended trench at WHSA Locality 2.

Sample #	AMS #	Elevation (masl)	Pollen ^{14}C age (ka)	Pollen $F^{14}C$	*Ruppia* ^{14}C age (ka)	*Ruppia* $F^{14}C$	HWE correction factor[3]
22WHSA1-18.1hh	USGS-2330	1196.86	18.82 ± 0.18	0.0961 ± 0.0022	--	--	--
22WHSA1-18.1k[1]	USGS-2073	1196.74	18.82 ± 0.18	0.0960 ± 0.0022	19.57 ± 0.10	0.0875 ± 0.0011	0.0085 ± 0.0024
22WHSA1-18.1n/nn	USGS-2074/2331	1196.52	18.83 ± 0.18	0.0959 ± 0.0021	20.16 ± 0.10	0.0813 ± 0.0010	0.0146 ± 0.0024
22WHSA1-18.1q[2]	USGS-2075	1196.34	19.30 ± 0.19	0.0904 ± 0.0021	20.49 ± 0.11	0.0780 ± 0.0011	0.0124 ± 0.0024
22WHSA1-18.1r/rr	USGS-2076/2332	1196.26	19.52 ± 0.20	0.0880 ± 0.0022	20.64 ± 0.11	0.0766 ± 0.0010	0.0115 ± 0.0024

Uncertainties are all given at the 68% (1σ) confidence level.

[1] The estimated pollen ^{14}C age for sample 22WHSA1-18.1k was determined by linearly interpolating between the pollen ages for samples 22WHSA1-18.1hh and 22WHSA1-18.1nn.

[2] The estimated pollen ^{14}C age for sample 22WHSA1-18.1q was determined by linearly interpolating between the pollen ages for samples 22WHSA1-18.1nn and 22WHSA1-18.1rr.

[3] HWE correction factors for each sample were determined by subtracting the *Ruppia* $F^{14}C$ value from the corresponding pollen $F^{14}C$ value.

Table S8

Summary of information used for correcting carbon-14 ages of *Ruppia* seeds from the extended trench at WHSA Locality 2 assuming a variable hard-water effect.

Sample #	AMS #	Elevation (masl)	Cal age (ka)	^{14}C age (ka)	$F^{14}C$	HWE correction factor[2]	Corrected $F^{14}C$[3]	Corrected ^{14}C age (ka)[4]	Corrected calibrated age range (ka)[5]	Corrected mean calibrated age (ka)[6]	HWE offset (^{14}C ka)
22WHSA1-15.1jj	USGS-2065	1197.76	21.58 ± 0.33	17.76 ± 0.08	0.1096 ± 0.0011	0.0000 ± 0.0000	0.1096 ± 0.0011	17.76 ± 0.08	21.91 - 21.25	21.58 ± 0.33	0.00
22WHSA1-15.1kk	USGS-2066	1197.72	22.15 ± 0.18	18.19 ± 0.08	0.1039 ± 0.0010	0.0000 ± 0.0000	0.1039 ± 0.0010	18.19 ± 0.08	22.34 - 21.97	22.15 ± 0.18	0.00
22WHSA1-15.1mm	USGS-2067	1197.64	22.32 ± 0.17	18.41 ± 0.09	0.1011 ± 0.0011	0.0000 ± 0.0000	0.1011 ± 0.0011	18.41 ± 0.09	22.49 - 22.15	22.32 ± 0.17	0.00
22WHSA1-15.1rr	USGS-2068	1197.46	22.64 ± 0.25	18.65 ± 0.09	0.0981 ± 0.0011	0.0000 ± 0.0000	0.0981 ± 0.0011	18.65 ± 0.09	22.89 - 22.39	22.64 ± 0.25	0.00
20WHSA1-18.1a	USGS-1803	1197.36	21.13 ± 0.25	17.47 ± 0.09	0.1136 ± 0.0013	0.0000 ± 0.0000	0.1136 ± 0.0013	17.47 ± 0.09	21.38 - 20.88	21.13 ± 0.25	0.00
20WHSA1-18.1a[1]	USGS-1828	1197.36	21.75 ± 0.31	17.93 ± 0.09	0.1073 ± 0.0012	0.0000 ± 0.0000	0.1073 ± 0.0012	17.93 ± 0.09	22.06 - 21.44	21.75 ± 0.31	0.00
20WHSA1-18.1e	USGS-1806	1197.36	22.15 ± 0.23	18.20 ± 0.11	0.1038 ± 0.0014	0.0000 ± 0.0000	0.1038 ± 0.0014	18.20 ± 0.11	22.39 - 21.92	22.15 ± 0.23	0.00
22WHSA1-15.1vv1	USGS-2069	1197.36	22.75 ± 0.23	18.86 ± 0.09	0.0956 ± 0.0011	0.0000 ± 0.0000	0.0956 ± 0.0011	18.86 ± 0.09	22.98 - 22.52	22.75 ± 0.23	0.00
20WHSA1-18.1b	USGS-1804	1197.30	21.68 ± 0.30	17.83 ± 0.09	0.1087 ± 0.0012	0.0000 ± 0.0000	0.1087 ± 0.0012	17.83 ± 0.09	21.98 - 21.38	21.68 ± 0.30	0.00
22WHSA1-15.1vv2	USGS-2070	1197.30	22.69 ± 0.24	18.76 ± 0.09	0.0968 ± 0.0011	0.0000 ± 0.0000	0.0968 ± 0.0011	18.76 ± 0.09	22.93 - 22.46	22.69 ± 0.24	0.00
20WHSA1-18.1d	USGS-1805	1197.28	22.55 ± 0.28	18.55 ± 0.10	0.0993 ± 0.0012	0.0000 ± 0.0000	0.0993 ± 0.0012	18.55 ± 0.10	22.83 - 22.28	22.55 ± 0.28	0.00
20WHSA1-18.1d[1]	USGS-1829	1197.28	22.64 ± 0.26	18.66 ± 0.10	0.0980 ± 0.0012	0.0000 ± 0.0000	0.0980 ± 0.0012	18.66 ± 0.10	22.90 - 22.39	22.64 ± 0.26	0.00
22WHSA1-15.1xx	USGS-2071	1197.28	22.63 ± 0.25	18.64 ± 0.09	0.0982 ± 0.0011	0.0000 ± 0.0000	0.0982 ± 0.0011	18.64 ± 0.09	22.88 - 22.38	22.63 ± 0.25	0.00
20WHSA1-18.1f	USGS-1807	1197.27	22.65 ± 0.26	18.68 ± 0.11	0.0977 ± 0.0013	0.0000 ± 0.0000	0.0977 ± 0.0013	18.68 ± 0.11	22.91 - 22.39	22.65 ± 0.26	0.00
20WHSA1-18.2a[1]	USGS-1808.1	1197.14	22.80 ± 0.26	18.95 ± 0.11	0.0945 ± 0.0013	0.0000 ± 0.0000	0.0945 ± 0.0013	18.95 ± 0.11	23.06 - 22.53	22.80 ± 0.26	0.00
20WHSA1-18.2a	USGS-1808	1197.14	22.87 ± 0.30	19.02 ± 0.11	0.0937 ± 0.0013	0.0000 ± 0.0000	0.0937 ± 0.0013	19.02 ± 0.11	23.17 - 22.57	22.87 ± 0.30	0.00
20WHSA1-18.3a[1]	USGS-1830	1197.14	22.72 ± 0.24	18.81 ± 0.10	0.0962 ± 0.0012	0.0000 ± 0.0000	0.0962 ± 0.0012	18.81 ± 0.10	22.96 - 22.48	22.72 ± 0.24	0.00
20WHSA1-18.3a	USGS-1810	1197.14	22.86 ± 0.32	19.01 ± 0.12	0.0938 ± 0.0014	0.0000 ± 0.0000	0.0938 ± 0.0014	19.01 ± 0.12	23.18 - 22.55	22.86 ± 0.32	0.00
22WHSA1-18.1k	USGS-2073	1196.74	23.54 ± 0.27	19.57 ± 0.10	0.0875 ± 0.0011	0.0085 ± 0.0024	0.0960 ± 0.0026	18.82 ± 0.22	23.19 - 22.32	22.75 ± 0.44	0.75
22WHSA1-18.1n	USGS-2074	1196.52	24.21 ± 0.32	20.16 ± 0.10	0.0813 ± 0.0010	0.0146 ± 0.0024	0.0911 ± 0.0027	19.24 ± 0.24	23.79 - 22.68	23.23 ± 0.56	0.92
22WHSA1-18.1q	USGS-2075	1196.34	24.63 ± 0.37	20.49 ± 0.11	0.0780 ± 0.0011	0.0124 ± 0.0024	0.0928 ± 0.0030	19.10 ± 0.26	23.74 - 22.50	23.12 ± 0.62	1.39
22WHSA1-18.1r	USGS-2076	1196.26	24.87 ± 0.34	20.64 ± 0.11	0.0766 ± 0.0010	0.0115 ± 0.0024	0.0870 ± 0.0030	19.62 ± 0.28	24.22 - 22.99	23.61 ± 0.62	1.02

Uncertainties for the calibrated ages are given at the 2σ (95%) confidence level; uncertainties for the ^{14}C ages and $F^{14}C$ values are given at 1σ (68%).

[1] Replicate samples.

[2] HWE correction factors were determined in Table S7.

[3] Corrected $F^{14}C$ values were determined by adding the HWE correction factor to the measured $F^{14}C$ value for each sample.

[4] Corrected ^{14}C ages were calculated using the corrected $F^{14}C$ values.

[5] Calibrated age ranges were calculated using OxCal v.4.4.4, IntCal20.14C dataset; limit 55.0 calendar ka B.P.

[6] Uncertainties of the mean calibrated ages represent the difference between the mean age and the upper/lower limits of the age range.

Table S9a

Summary of information for modeled ages based on carbon-14 ages of *Ruppia* seeds only, uncorrected (Model 1).

TRACK HORIZONS/PACKAGES

	Thickness (cm)	Mean elevation (masl)		Elevation (masl)	Modeled age (ka)	+/- (2σ)
TP16	14	1198.08			21.27	0.37
TP15	5	1197.98			21.44	0.31
TP14	4	1197.94			21.53	0.29
TP13	3	1197.90			21.59	0.27
TP12	7	1197.85			21.69	0.23
TP11	5	1197.79			21.80	0.19
TP10	11	1197.71			22.02	0.13
TP9	12	1197.60			22.21	0.11
TP8	10	1197.49			22.34	0.08
			TH7	1197.39	22.43	0.07
			TH6	1197.36	22.45	0.07
			TH5	1197.27	22.61	0.12
			TH4	1197.22	22.71	0.15
			TH3	1197.19	22.77	0.15
			TH2	1197.14	22.86	0.16
			TH1	1197.08	22.98	0.19
			TH0	1196.60	23.96	0.22
			TH(-1)	1196.52	24.13	0.21
			TH(-2)	1196.34	24.55	0.26
			TH(-3)	1196.26	24.71	0.29
			Top of sequence	1198.15	21.14	0.41
			Base of sequence	1195.48	26.16	0.73

RADIOCARBON SAMPLES

Sample ID	AMS ID	Elevation (masl)	Modeled age (ka)	+/- (2σ)
22WHSA1-15.1j	USGS-2065	1197.76	21.87	0.16
22WHSA1-15.1k	USGS-2066	1197.72	22.02	0.13
22WHSA1-15.1m	USGS-2067	1197.64	22.17	0.11
22WHSA1-15.1rr	USGS-2068	1197.46	22.38	0.07
20WHSA1-18.1a	USGS-1828	1197.36	22.45	0.07
20WHSA1-18.1e	USGS-1806	1197.36	22.45	0.07
22WHSA1-15.1vv1	USGS-2069	1197.36	22.45	0.07
22WHSA1-15.1vv2	USGS-2070	1197.30	22.56	0.11
20WHSA1-18.1d	USGS-1805	1197.28	22.59	0.12
20WHSA1-18.1d	USGS-1829	1197.28	22.59	0.12
22WHSA1-15.1xx	USGS-2071	1197.28	22.59	0.12
20WHSA1-18.1f	USGS-1807	1197.27	22.62	0.13
20WHSA1-18.2a	USGS-1808.1	1197.14	22.85	0.15
20WHSA1-18.2a	USGS-1808	1197.14	22.85	0.15
20WHSA1-18.3a	USGS-1830	1197.14	22.85	0.15
20WHSA1-18.3a	USGS-1810	1197.14	22.85	0.15
22WHSA1-18.1k	USGS-2073	1196.74	23.64	0.18
22WHSA1-18.1n	USGS-2074	1196.52	24.14	0.21
22WHSA1-18.1q	USGS-2075	1196.34	24.55	0.26
22WHSA1-18.1r	USGS-2076	1196.26	24.71	0.29

PROXY SAMPLES

Sample ID	Thickness (cm)	Mean elevation (masl)	Modeled age (ka)	+/- (2σ)	Sample ID	Thickness (cm)	Mean elevation (masl)	Modeled age (ka)	+/- (2σ)	Sample ID	Thickness (cm)	Mean elevation (masl)	Modeled age (ka)	+/- (2σ)
22WHSA1-15.1a	4	1198.13	21.18	0.39										
22WHSA1-15.1b	5	1198.08	21.26	0.37										
22WHSA1-15.1c	5	1198.03	21.35	0.34										
22WHSA1-15.1d	5	1197.98	21.44	0.31										
22WHSA1-15.1e	4	1197.94	21.53	0.29										
22WHSA1-15.1f	3	1197.90	21.59	0.27										
22WHSA1-15.1g	4	1197.87	21.66	0.24										
22WHSA1-15.1h	3	1197.83	21.73	0.22										
22WHSA1-15.1i	5	1197.79	21.80	0.19										
22WHSA1-15.1j	2	1197.76	21.87	0.16										
22WHSA1-15.1k	6	1197.72	22.01	0.13										
22WHSA1-15.1l	3	1197.67	22.10	0.12										
22WHSA1-15.1m	4	1197.64	22.17	0.11										
22WHSA1-15.1n	2	1197.61	22.20	0.11										
22WHSA1-15.1o	4	1197.58	22.24	0.10										
22WHSA1-15.1p	2	1197.55	22.27	0.10										
22WHSA1-15.1q	5	1197.51	22.31	0.09										
22WHSA1-15.1r	5	1197.46	22.37	0.08										
22WHSA1-15.1s	4	1197.42	22.40	0.07	22WHSA1-17.1a	4	1197.42	22.40	0.07					
22WHSA1-15.1t	2	1197.39	22.42	0.07	22WHSA1-17.1b	4	1197.38	22.43	0.07					
22WHSA1-15.1u	2	1197.37	22.44	0.07										
22WHSA1-15.1v	3	1197.35	22.47	0.08	22WHSA1-17.1c	6	1197.33	22.50	0.09					
22WHSA1-15.1w	3	1197.32	22.53	0.10										
22WHSA1-15.1x	3	1197.28	22.58	0.11	22WHSA1-17.1d	3	1197.28	22.58	0.11					
22WHSA1-15.1y	5	1197.24	22.66	0.14	22WHSA1-17.1e	5	1197.24	22.66	0.14					
22WHSA1-15.1z	4	1197.20	22.74	0.15	22WHSA1-17.1f	4	1197.20	22.74	0.15					
					22WHSA1-17.1g	3	1197.17	22.80	0.15					
					22WHSA1-17.1h	2	1197.15	22.84	0.15					
					22WHSA1-17.1i	3	1197.12	22.89	0.17	22WHSA1-18.1a	3	1197.12	22.89	0.17
					22WHSA1-17.1j	4	1197.09	22.96	0.18	22WHSA1-18.1b	2	1197.10	22.94	0.18
										22WHSA1-18.1c	2	1197.08	22.98	0.19
										22WHSA1-18.1d	3	1197.06	23.03	0.20
										22WHSA1-18.1e	6	1197.01	23.12	0.21
										22WHSA1-18.1f	4	1196.96	23.22	0.21
										22WHSA1-18.1g	6	1196.91	23.32	0.22
										22WHSA1-18.1h	4	1196.86	23.42	0.21
										22WHSA1-18.1i	4	1196.82	23.49	0.21
										22WHSA1-18.1j	6	1196.77	23.58	0.19
										22WHSA1-18.1k	5	1196.72	23.69	0.20
										22WHSA1-18.1l	9	1196.65	23.86	0.22
										22WHSA1-18.1m	8	1196.56	24.05	0.22
										22WHSA1-18.1n	5	1196.50	24.19	0.22
										22WHSA1-18.1o	5	1196.45	24.31	0.24
										22WHSA1-18.1p	7	1196.39	24.44	0.25
										22WHSA1-18.1q	7	1196.32	24.59	0.27
										22WHSA1-18.1r	13	1196.22	24.79	0.32
										22WHSA1-18.1s	5	1196.13	24.96	0.37
										22WHSA1-18.1t	5	1196.08	25.06	0.39
										22WHSA1-18.1u	5	1196.03	25.15	0.42
										22WHSA1-18.1v1	7	1195.97	25.26	0.45
										22WHSA1-18.1v2	7	1195.90	25.40	0.49
										22WHSA1-18.1v3	7	1195.83	25.52	0.52
										22WHSA1-18.1v4	7	1195.76	25.66	0.56
										22WHSA1-18.1w	7	1195.69	25.78	0.59
										22WHSA1-18.1x1	8	1195.61	25.92	0.63
										22WHSA1-18.1x2	9	1195.53	26.09	0.68

Table S9b

Summary of information for modeled ages based on carbon-14 ages of *Ruppia* seeds and pollen only, uncorrected (Model 2).

TRACK HORIZONS/PACKAGES

	Thickness (cm)	Mean elevation (masl)		Elevation (masl)	Modeled age (ka)	+/- (2σ)
TP16	14	1198.08			21.34	0.33
TP15	5	1197.98			21.51	0.28
TP14	4	1197.94			21.58	0.26
TP13	3	1197.90			21.64	0.24
TP12	7	1197.85			21.73	0.21
TP11	5	1197.79			21.83	0.17
TP10	11	1197.71			22.03	0.12
TP9	12	1197.60			22.21	0.10
TP8	10	1197.49			22.34	0.08
			TH7	1197.39	22.42	0.07
			TH6	1197.36	22.44	0.06
			TH5	1197.27	22.59	0.11
			TH4	1197.22	22.68	0.14
			TH3	1197.19	22.74	0.15
			TH2	1197.14	22.81	0.16
			TH1	1197.08	22.90	0.18
			TH0	1196.60	23.79	0.19
			TH(-1)	1196.52	23.95	0.16
			TH(-2)	1196.34	24.35	0.18
			TH(-3)	1196.26	24.48	0.20
			Top of sequence	1198.15	21.23	0.37
			Base of sequence	1195.48	25.81	0.58

RADIOCARBON SAMPLES

Sample ID	AMS ID	Elevation (masl)	Modeled age (ka)	+/- (2σ)
22WHSA1-15.1j	USGS-2065	1197.76	21.89	0.15
22WHSA1-15.1k	USGS-2066	1197.72	22.02	0.12
22WHSA1-15.1m	USGS-2067	1197.64	22.17	0.10
22WHSA1-15.1rr	USGS-2068	1197.46	22.37	0.07
20WHSA1-18.1a	USGS-1828	1197.36	22.45	0.06
20WHSA1-18.1e	USGS-1806	1197.36	22.45	0.06
22WHSA1-15.1vv1	USGS-2069	1197.36	22.45	0.06
20WHSA1-18.1aa	CAMS-190357A	1197.36	22.45	0.06
22WHSA1-15.1vv2	USGS-2070	1197.30	22.54	0.09
20WHSA1-18.1d	USGS-1805	1197.28	22.57	0.10
20WHSA1-18.1d	USGS-1829	1197.28	22.57	0.10
22WHSA1-15.1xx	USGS-2071	1197.28	22.57	0.10
20WHSA1-18.1dd	CAMS-190358A	1197.28	22.57	0.10
20WHSA1-18.1f	USGS-1807	1197.27	22.59	0.11
20WHSA1-18.1ff	CAMS-190359A	1197.27	22.59	0.11
20WHSA1-18.2a	USGS-1808.1	1197.14	22.80	0.16
20WHSA1-18.2a	USGS-1808	1197.14	22.80	0.16
20WHSA1-18.3a	USGS-1830	1197.14	22.80	0.16
20WHSA1-18.3a	USGS-1810	1197.14	22.80	0.16
22WHSA1-18.1hh	USGS-2330	1196.86	23.22	0.21
22WHSA1-18.1k	USGS-2073	1196.74	23.53	0.20
22WHSA1-18.1n	USGS-2074	1196.52	23.96	0.16
22WHSA1-18.1nn	USGS-2331	1196.52	23.96	0.16
22WHSA1-18.1q	USGS-2075	1196.34	24.35	0.18
22WHSA1-18.1r	USGS-2076	1196.26	24.48	0.21
22WHSA1-18.1rr	USGS-2332	1196.26	24.48	0.21

PROXY SAMPLES

Sample ID	Thickness (cm)	Mean elevation (masl)	Modeled age (ka)	+/- (2σ)	Sample ID	Thickness (cm)	Mean elevation (masl)	Modeled age (ka)	+/- (2σ)	Sample ID	Thickness (cm)	Mean elevation (masl)	Modeled age (ka)	+/- (2σ)
22WHSA1-15.1a	4	1198.13	21.26	0.36										
22WHSA1-15.1b	5	1198.08	21.34	0.33										
22WHSA1-15.1c	5	1198.03	21.42	0.31										
22WHSA1-15.1d	5	1197.98	21.51	0.28										
22WHSA1-15.1e	4	1197.94	21.58	0.26										
22WHSA1-15.1f	3	1197.90	21.64	0.24										
22WHSA1-15.1g	4	1197.87	21.70	0.22										
22WHSA1-15.1h	3	1197.83	21.76	0.20										
22WHSA1-15.1i	5	1197.79	21.83	0.17										
22WHSA1-15.1j	2	1197.76	21.90	0.15										
22WHSA1-15.1k	6	1197.72	22.01	0.12										
22WHSA1-15.1l	3	1197.67	22.10	0.12										
22WHSA1-15.1m	4	1197.64	22.16	0.10										
22WHSA1-15.1n	2	1197.61	22.20	0.10										
22WHSA1-15.1o	4	1197.58	22.23	0.10										
22WHSA1-15.1p	2	1197.55	22.27	0.09										
22WHSA1-15.1q	5	1197.51	22.31	0.09										
22WHSA1-15.1r	5	1197.46	22.37	0.07										
22WHSA1-15.1s	4	1197.42	22.40	0.07	22WHSA1-17.1a	4	1197.42	22.40	0.07					
22WHSA1-15.1t	2	1197.39	22.42	0.07	22WHSA1-17.1b	4	1197.38	22.43	0.07					
22WHSA1-15.1u	2	1197.37	22.44	0.07										
22WHSA1-15.1v	3	1197.35	22.46	0.07	22WHSA1-17.1c	6	1197.33	22.49	0.08					
22WHSA1-15.1w	3	1197.32	22.52	0.09										
22WHSA1-15.1x	3	1197.28	22.56	0.10	22WHSA1-17.1d	3	1197.28	22.56	0.10					
22WHSA1-15.1y	5	1197.24	22.63	0.12	22WHSA1-17.1e	5	1197.24	22.63	0.12					
22WHSA1-15.1z	4	1197.20	22.71	0.14	22WHSA1-17.1f	4	1197.20	22.71	0.14					
					22WHSA1-17.1g	3	1197.17	22.76	0.15					
					22WHSA1-17.1h	2	1197.15	22.80	0.16					
					22WHSA1-17.1i	3	1197.12	22.84	0.16	22WHSA1-18.1a	3	1197.12	22.84	0.16
					22WHSA1-17.1j	4	1197.09	22.89	0.17	22WHSA1-18.1b	2	1197.10	22.87	0.17
										22WHSA1-18.1c	2	1197.08	22.90	0.18
										22WHSA1-18.1d	3	1197.06	22.94	0.18
										22WHSA1-18.1e	6	1197.01	23.01	0.19
										22WHSA1-18.1f	4	1196.96	23.08	0.20
										22WHSA1-18.1g	6	1196.91	23.15	0.20
										22WHSA1-18.1h	4	1196.86	23.22	0.21
										22WHSA1-18.1i	4	1196.82	23.32	0.21
										22WHSA1-18.1j	6	1196.77	23.43	0.20
										22WHSA1-18.1k	5	1196.72	23.56	0.19
										22WHSA1-18.1l	9	1196.65	23.70	0.19
										22WHSA1-18.1m	8	1196.56	23.87	0.18
										22WHSA1-18.1n	5	1196.50	24.00	0.17
										22WHSA1-18.1o	5	1196.45	24.11	0.18
										22WHSA1-18.1p	7	1196.39	24.24	0.19
										22WHSA1-18.1q	7	1196.32	24.38	0.19
										22WHSA1-18.1r	13	1196.22	24.55	0.23
										22WHSA1-18.1s	5	1196.13	24.71	0.28
										22WHSA1-18.1t	5	1196.08	24.79	0.31
										22WHSA1-18.1u	5	1196.03	24.88	0.33
										22WHSA1-18.1v1	7	1195.97	24.98	0.36
										22WHSA1-18.1v2	7	1195.90	25.10	0.40
										22WHSA1-18.1v3	7	1195.83	25.22	0.43
										22WHSA1-18.1v4	7	1195.76	25.34	0.46
										22WHSA1-18.1w	7	1195.69	25.46	0.49
										22WHSA1-18.1x1	8	1195.61	25.59	0.53
										22WHSA1-18.1x2	9	1195.53	25.73	0.57

Table S9c

Summary of information for modeled ages based on carbon-14 ages of *Ruppia* seeds only, assuming a constant HWE (Model 3).

TRACK HORIZONS/PACKAGES

	Thickness (cm)	Mean elevation (masl)		Elevation (masl)	Modeled age (ka)	+/- (2σ)
TP16	14	1198.08			20.29	0.63
TP15	5	1197.98			20.46	0.57
TP14	4	1197.94			20.54	0.54
TP13	3	1197.90			20.60	0.52
TP12	7	1197.85			20.69	0.49
TP11	5	1197.79			20.80	0.45
TP10	11	1197.71			20.94	0.40
TP9	12	1197.60			21.14	0.36
TP8	10	1197.49			21.33	0.32
			TH7	1197.39	21.49	0.29
			TH6	1197.36	21.54	0.28
			TH5	1197.27	21.71	0.26
			TH4	1197.22	21.80	0.27
			TH3	1197.19	21.86	0.27
			TH2	1197.14	21.95	0.27
			TH1	1197.08	22.05	0.28
			TH0	1196.60	22.90	0.38
			TH(-1)	1196.52	23.04	0.40
			TH(-2)	1196.34	23.37	0.47
			TH(-3)	1196.26	23.52	0.51
			Top of sequence	1198.15	20.16	0.68
			Base of sequence	1195.48	24.94	1.03

RADIOCARBON SAMPLES

Sample ID	AMS ID	Elevation (masl)	Modeled age (ka)	+/- (2σ)
22WHSA1-15.1j	USGS-2065	1197.76	20.86	0.43
22WHSA1-15.1k	USGS-2066	1197.72	20.94	0.40
22WHSA1-15.1m	USGS-2067	1197.64	21.08	0.37
22WHSA1-15.1rr	USGS-2068	1197.46	21.38	0.31
20WHSA1-18.1a	USGS-1828	1197.36	21.55	0.28
20WHSA1-18.1e	USGS-1806	1197.36	21.55	0.28
22WHSA1-15.1vv1	USGS-2069	1197.36	21.55	0.28
22WHSA1-15.1vv2	USGS-2070	1197.30	21.65	0.27
20WHSA1-18.1d	USGS-1805	1197.28	21.69	0.26
20WHSA1-18.1d	USGS-1829	1197.28	21.69	0.26
22WHSA1-15.1xx	USGS-2071	1197.28	21.69	0.26
20WHSA1-18.1f	USGS-1807	1197.27	21.71	0.26
20WHSA1-18.2a	USGS-1808.1	1197.14	21.93	0.27
20WHSA1-18.2a	USGS-1808	1197.14	21.93	0.27
20WHSA1-18.3a	USGS-1830	1197.14	21.93	0.27
20WHSA1-18.3a	USGS-1810	1197.14	21.93	0.27
22WHSA1-18.1k	USGS-2073	1196.74	22.65	0.33
22WHSA1-18.1n	USGS-2074	1196.52	23.04	0.41
22WHSA1-18.1q	USGS-2075	1196.34	23.38	0.47
22WHSA1-18.1r	USGS-2076	1196.26	23.52	0.51

PROXY SAMPLES

Sample ID	Thickness (cm)	Mean elevation (masl)	Modeled age (ka)	+/- (2σ)	Sample ID	Thickness (cm)	Mean elevation (masl)	Modeled age (ka)	+/- (2σ)	Sample ID	Thickness (cm)	Mean elevation (masl)	Modeled age (ka)	+/- (2σ)
22WHSA1-15.1a	4	1198.13	20.19	0.66										
22WHSA1-15.1b	5	1198.08	20.28	0.64										
22WHSA1-15.1c	5	1198.03	20.37	0.60										
22WHSA1-15.1d	5	1197.98	20.46	0.57										
22WHSA1-15.1e	4	1197.94	20.54	0.54										
22WHSA1-15.1f	3	1197.90	20.60	0.52										
22WHSA1-15.1g	4	1197.87	20.66	0.50										
22WHSA1-15.1h	3	1197.83	20.73	0.48										
22WHSA1-15.1i	5	1197.79	20.80	0.45										
22WHSA1-15.1j	2	1197.76	20.86	0.43										
22WHSA1-15.1k	6	1197.72	20.94	0.41										
22WHSA1-15.1l	3	1197.67	21.01	0.39										
22WHSA1-15.1m	4	1197.64	21.07	0.37										
22WHSA1-15.1n	2	1197.61	21.12	0.36										
22WHSA1-15.1o	4	1197.58	21.18	0.35										
22WHSA1-15.1p	2	1197.55	21.23	0.34										
22WHSA1-15.1q	5	1197.51	21.28	0.33										
22WHSA1-15.1r	5	1197.46	21.36	0.31										
22WHSA1-15.1s	4	1197.42	21.44	0.30	22WHSA1-17.1a	4	1197.42	21.44	0.30					
22WHSA1-15.1t	2	1197.39	21.49	0.29	22WHSA1-17.1b	4	1197.38	21.51	0.29					
22WHSA1-15.1u	2	1197.37	21.52	0.28										
22WHSA1-15.1v	3	1197.35	21.56	0.28	22WHSA1-17.1c	6	1197.33	21.59	0.27					
22WHSA1-15.1w	3	1197.32	21.62	0.27										
22WHSA1-15.1x	3	1197.28	21.68	0.26	22WHSA1-17.1d	3	1197.28	21.68	0.26					
22WHSA1-15.1y	5	1197.24	21.75	0.27	22WHSA1-17.1e	5	1197.24	21.75	0.27					
22WHSA1-15.1z	4	1197.20	21.83	0.27	22WHSA1-17.1f	4	1197.20	21.83	0.27					
					22WHSA1-17.1g	3	1197.17	21.88	0.27					
					22WHSA1-17.1h	2	1197.15	21.93	0.27					
					22WHSA1-17.1i	3	1197.12	21.97	0.27	22WHSA1-18.1a	3	1197.12	21.97	0.27
					22WHSA1-17.1j	4	1197.09	22.03	0.28	22WHSA1-18.1b	2	1197.10	22.01	0.28
										22WHSA1-18.1c	2	1197.08	22.05	0.28
										22WHSA1-18.1d	3	1197.06	22.09	0.28
										22WHSA1-18.1e	6	1197.01	22.17	0.29
										22WHSA1-18.1f	4	1196.96	22.26	0.30
										22WHSA1-18.1g	6	1196.91	22.35	0.31
										22WHSA1-18.1h	4	1196.86	22.44	0.31
										22WHSA1-18.1i	4	1196.82	22.51	0.32
										22WHSA1-18.1j	6	1196.77	22.60	0.33
										22WHSA1-18.1k	5	1196.72	22.69	0.34
										22WHSA1-18.1l	9	1196.65	22.82	0.36
										22WHSA1-18.1m	8	1196.56	22.97	0.39
										22WHSA1-18.1n	5	1196.50	23.09	0.41
										22WHSA1-18.1o	5	1196.45	23.18	0.43
										22WHSA1-18.1p	7	1196.39	23.29	0.45
										22WHSA1-18.1q	7	1196.32	23.41	0.48
										22WHSA1-18.1r	13	1196.22	23.59	0.54
										22WHSA1-18.1s	5	1196.13	23.75	0.60
										22WHSA1-18.1t	5	1196.08	23.84	0.63
										22WHSA1-18.1u	5	1196.03	23.94	0.66
										22WHSA1-18.1v1	7	1195.97	24.05	0.70
										22WHSA1-18.1v2	7	1195.90	24.17	0.74
										22WHSA1-18.1v3	7	1195.83	24.29	0.79
										22WHSA1-18.1v4	7	1195.76	24.42	0.83
										22WHSA1-18.1w	7	1195.69	24.54	0.88
										22WHSA1-18.1x1	8	1195.61	24.68	0.92
										22WHSA1-18.1x2	9	1195.53	24.83	0.98

Table S9d

Summary of information for modeled ages based on carbon-14 ages of *Ruppia* seeds only, assuming a variable HWE (Model 4).

TRACK HORIZONS/PACKAGES

	Thickness (cm)	Mean elevation (masl)		Elevation (masl)	Modeled age (ka)	+/- (2σ)
TP16	14	1198.08			21.68	0.25
TP15	5	1197.98			21.78	0.22
TP14	4	1197.94			21.82	0.20
TP13	3	1197.90			21.86	0.19
TP12	7	1197.85			21.91	0.17
TP11	5	1197.79			21.97	0.14
TP10	11	1197.71			22.08	0.10
TP9	12	1197.60			22.22	0.09
TP8	10	1197.49			22.33	0.07
			TH7	1197.39	22.41	0.06
			TH6	1197.36	22.43	0.06
			TH5	1197.27	22.52	0.07
			TH4	1197.22	22.57	0.08
			TH3	1197.19	22.61	0.09
			TH2	1197.14	22.66	0.10
			TH1	1197.08	22.70	0.11
			TH0	1196.60	23.14	0.22
			TH(-1)	1196.52	23.22	0.24
			TH(-2)	1196.34	23.40	0.29
			TH(-3)	1196.26	23.49	0.32
			Top of sequence	1198.15	21.62	0.27
			Base of sequence	1195.48	24.23	0.61

RADIOCARBON SAMPLES

Sample ID	AMS ID	Elevation (masl)	Modeled age (ka)	+/- (2σ)
22WHSA1-15.1j	USGS-2065	1197.76	22.00	0.13
22WHSA1-15.1k	USGS-2066	1197.72	22.08	0.10
22WHSA1-15.1m	USGS-2067	1197.64	22.18	0.09
22WHSA1-15.1rr	USGS-2068	1197.46	22.36	0.07
20WHSA1-18.1a	USGS-1828	1197.36	22.43	0.06
20WHSA1-18.1e	USGS-1806	1197.36	22.43	0.06
22WHSA1-15.1vv1	USGS-2069	1197.36	22.43	0.06
22WHSA1-15.1vv2	USGS-2070	1197.30	22.49	0.06
20WHSA1-18.1d	USGS-1805	1197.28	22.51	0.06
20WHSA1-18.1d	USGS-1829	1197.28	22.51	0.06
22WHSA1-15.1xx	USGS-2071	1197.28	22.51	0.06
20WHSA1-18.1f	USGS-1807	1197.27	22.52	0.07
20WHSA1-18.2a	USGS-1808.1	1197.14	22.65	0.09
20WHSA1-18.2a	USGS-1808	1197.14	22.65	0.09
20WHSA1-18.3a	USGS-1830	1197.14	22.65	0.09
20WHSA1-18.3a	USGS-1810	1197.14	22.65	0.09
22WHSA1-18.1k	USGS-2073	1196.74	23.01	0.19
22WHSA1-18.1n	USGS-2074	1196.52	23.23	0.24
22WHSA1-18.1q	USGS-2075	1196.34	23.41	0.29
22WHSA1-18.1r	USGS-2076	1196.26	23.49	0.32

PROXY SAMPLES

Sample ID	Thickness (cm)	Mean elevation (masl)	Modeled age (ka)	+/- (2σ)	Sample ID	Thickness (cm)	Mean elevation (masl)	Modeled age (ka)	+/- (2σ)	Sample ID	Thickness (cm)	Mean elevation (masl)	Modeled age (ka)	+/- (2σ)
22WHSA1-15.1a	4	1198.13	21.64	0.27										
22WHSA1-15.1b	5	1198.08	21.68	0.25										
22WHSA1-15.1c	5	1198.03	21.73	0.24										
22WHSA1-15.1d	5	1197.98	21.78	0.22										
22WHSA1-15.1e	4	1197.94	21.82	0.20										
22WHSA1-15.1f	3	1197.90	21.86	0.19										
22WHSA1-15.1g	4	1197.87	21.89	0.17										
22WHSA1-15.1h	3	1197.83	21.93	0.16										
22WHSA1-15.1i	5	1197.79	21.97	0.14										
22WHSA1-15.1j	2	1197.76	22.01	0.13										
22WHSA1-15.1k	6	1197.72	22.07	0.10										
22WHSA1-15.1l	3	1197.67	22.13	0.10										
22WHSA1-15.1m	4	1197.64	22.18	0.09										
22WHSA1-15.1n	2	1197.61	22.21	0.09										
22WHSA1-15.1o	4	1197.58	22.24	0.09										
22WHSA1-15.1p	2	1197.55	22.27	0.08										
22WHSA1-15.1q	5	1197.51	22.31	0.08										
22WHSA1-15.1r	5	1197.46	22.36	0.07										
22WHSA1-15.1s	4	1197.42	22.39	0.06	22WHSA1-17.1a	4	1197.42	22.39	0.06					
22WHSA1-15.1t	2	1197.39	22.41	0.06	22WHSA1-17.1b	4	1197.38	22.42	0.06					
22WHSA1-15.1u	2	1197.37	22.42	0.06										
22WHSA1-15.1v	3	1197.35	22.44	0.06	22WHSA1-17.1c	6	1197.33	22.46	0.06					
22WHSA1-15.1w	3	1197.32	22.48	0.06										
22WHSA1-15.1x	3	1197.28	22.51	0.06	22WHSA1-17.1d	3	1197.28	22.51	0.06					
22WHSA1-15.1y	5	1197.24	22.55	0.07	22WHSA1-17.1e	5	1197.24	22.55	0.07					
22WHSA1-15.1z	4	1197.20	22.59	0.08	22WHSA1-17.1f	4	1197.20	22.59	0.08					
					22WHSA1-17.1g	3	1197.17	22.62	0.09					
					22WHSA1-17.1h	2	1197.15	22.64	0.09					
					22WHSA1-17.1i	3	1197.12	22.67	0.10	22WHSA1-18.1a	3	1197.12	22.67	0.10
					22WHSA1-17.1j	4	1197.09	22.69	0.11	22WHSA1-18.1b	2	1197.10	22.69	0.10
										22WHSA1-18.1c	2	1197.08	22.70	0.11
										22WHSA1-18.1d	3	1197.06	22.73	0.12
										22WHSA1-18.1e	6	1197.01	22.77	0.13
										22WHSA1-18.1f	4	1196.96	22.81	0.14
										22WHSA1-18.1g	6	1196.91	22.85	0.15
										22WHSA1-18.1h	4	1196.86	22.90	0.16
										22WHSA1-18.1i	4	1196.82	22.94	0.17
										22WHSA1-18.1j	6	1196.77	22.98	0.18
										22WHSA1-18.1k	5	1196.72	23.03	0.19
										22WHSA1-18.1l	9	1196.65	23.10	0.21
										22WHSA1-18.1m	8	1196.56	23.18	0.23
										22WHSA1-18.1n	5	1196.50	23.25	0.25
										22WHSA1-18.1o	5	1196.45	23.30	0.26
										22WHSA1-18.1p	7	1196.39	23.36	0.28
										22WHSA1-18.1q	7	1196.32	23.43	0.30
										22WHSA1-18.1r	13	1196.22	23.52	0.33
										22WHSA1-18.1s	5	1196.13	23.61	0.37
										22WHSA1-18.1t	5	1196.08	23.66	0.38
										22WHSA1-18.1u	5	1196.03	23.71	0.40
										22WHSA1-18.1v1	7	1195.97	23.77	0.42
										22WHSA1-18.1v2	7	1195.90	23.83	0.44
										22WHSA1-18.1v3	7	1195.83	23.90	0.47
										22WHSA1-18.1v4	7	1195.76	23.97	0.49
										22WHSA1-18.1w	7	1195.69	24.04	0.52
										22WHSA1-18.1x1	8	1195.61	24.11	0.54
										22WHSA1-18.1x2	9	1195.53	24.19	0.57

Table S10
Summary of modern playa biomarker data from White Sands National Park.

SampleID	C26dD	C28dD	C30dD	C32dD	C26dDSE	C28dDSE	C30dDSE	C32dDSE	C26d13C	C28d13C	C30d13C	C32d13C	C26d13CSE	C28d13CSE	C30d13CSE	C32d13CSE
HR10fame	-158.2	-139.8	-146.8	-157.4	0.2	0.8	1.6	0.7	-25.3	-25.0	-24.3	-24.0	0.1	0.1	0.0	0.0
HR11fame	-157.2	-142.1	-148.9	-160.1	1.4	0.9	0.5	1.0	-25.4	-25.0	-24.2	-24.0	0.1	0.1	0.1	0.1

Table S11
Summary of GDGT data from WHSA Locality 2.

LayerID	gExtracted	Elevation	Depth	Age	AgeError	Archaeol	GDGT0	GDGT1	GDGT2	GDGT3	Cren	CrenPr
15.1a	53.775	1198.13	0.00	21.64	0.26	2463017	420880	32710	31143	19672	155875	20731
15.1b	46.723	1198.08	4.50	21.68	0.24	396947	213523	12862	14434	9169	110658	13094
15.1c	55.647	1198.03	9.50	21.73	0.22	359148	54792	6988	7455	4894	57173	6421
15.1d	60.607	1197.98	14.50	21.78	0.21	11238	86080	17923	16559	16402	202131	22629
15.1e	59.725	1197.94	19.00	21.83	0.19	17799	43255	6596	6500	3297	44287	5321
15.1f	27.649	1197.90	22.50	21.86	0.18	164828	129367	19949	23018	14344	212945	20062
15.1g	52.685	1197.87	26.00	21.90	0.17	26687	331373	28872	38537	21281	250468	28204
15.1h	53.207	1197.83	29.50	21.93	0.15	38912	139150	4180	6298	2142	30299	4236
15.1i	51.284	1197.79	33.50	21.97	0.14	62590	174983	8072	19502	4027	91589	7986
15.1j	48.294	1197.76	37.00	22.01	0.12	14404	107767	7925	12117	5586	67540	6373
15.1k	54.48	1197.72	41.00	22.07	0.10	21016	503248	32004	52819	21276	321002	28022
15.1l	54.308	1197.67	45.50	22.13	0.10	91900	123194	5831	12950	3295	59935	5817
15.1m	37.828	1197.64	49.00	22.18	0.09	286101	600190	18430	37209	7344	103106	8977
15.1n	52.09	1197.61	52.00	22.21	0.09	177408	384934	18205	36739	8106	143033	9438
15.1o	60.155	1197.58	55.00	22.24	0.09	77989	805900	21994	61382	8763	166378	6763
15.1p	57.991	1197.55	58.00	22.27	0.08	16931	324516	17184	38995	8366	161105	8573
15.1q	48.906	1197.51	61.50	22.31	0.08	265838	2076045	61953	158882	18904	384434	13212
15.1r	45.233	1197.46	66.50	22.36	0.07	43716	587119	22532	51480	5976	131070	3647
15.1s	59.734	1197.42	71.00	22.39	0.06	1291545	894813	24943	51118	5439	157715	3871
15.1t	57.846	1197.39	73.94	22.41	0.06	15117	356883	7648	26449	1308	57977	1618
15.1u	53.267	1197.37	75.81	22.42	0.06	412814	1021781	25870	47521	2099	76391	2131
15.1v	58.414	1197.35	78.28	22.44	0.06	46980	1164245	35108	55789	3672	123115	2928
15.1w	57.627	1197.32	81.33	22.48	0.06	49781	834031	23551	43945	3457	110086	2492
15.1x	50.03	1197.28	84.48	22.51	0.06	198999	495502	20642	31183	2828	93038	2769
15.1y	62.135	1197.24	88.48	22.55	0.07	214153	665323	27750	47212	3736	160846	2979
15.1z	50.902	1197.20	92.63	22.59	0.08	316355	460463	10881	25856	1246	26537	2019
17.1g	48.069	1197.17	95.67	22.62	0.09	716464	586209	21207	32298	1185	19973	1796
17.1h	49.545	1197.15	98.08	22.65	0.09	347538	381570	6924	18112	834	24904	1686
17.1i	44.49	1197.12	100.60	22.67	0.10	539297	524003	15556	40412	2180	55265	1797
17.1j	36.18	1197.09	103.85	22.70	0.11	11264684	2705007	82877	167285	6313	87137	7820
18.1c	52.406	1197.08	104.78	22.71	0.11	1308206	2695599	118062	153909	23490	261639	19124
18.1d	51.434	1197.06	107.21	22.73	0.12	10523759	3137754	132473	202283	12059	191684	14281
18.1e	59.926	1197.01	111.72	22.77	0.13	7514444	634565	25080	32961	4405	64174	4293
18.1f	44.16	1196.96	116.71	22.82	0.14	203600	1788882	47308	87074	12253	159813	9821
18.1g	57.548	1196.91	121.71	22.86	0.15	5639312	801871	21049	32759	4899	70089	4195
18.1h	55.313	1196.86	126.72	22.91	0.16	18074932	3083173	85488	141327	13425	119314	8035
18.1i	56.77	1196.82	130.72	22.95	0.17	354971	1063990	22355	43321	2475	36352	1205
18.1j	56.769	1196.77	135.72	22.99	0.18	3572763	1700307	42746	74003	3467	79916	2901
18.1k	58.047	1196.72	141.22	23.05	0.20	3472603	865594	29674	41719	5347	83556	3807
18.1l	56.512	1196.65	148.22	23.11	0.21	157365	1305279	24019	54149	2385	48626	1865
18.1m	46.667	1196.56	156.72	23.19	0.23	13584107	3963332	110354	220658	16543	176025	14840
18.1n	53.471	1196.50	163.22	23.25	0.25	6552346	1265137	23327	38023	4591	71977	6002
18.1o	55.551	1196.45	168.22	23.30	0.26	5558581	1631347	30608	48448	3258	58593	2571
18.1p	52.726	1196.39	174.22	23.36	0.28	117702	3620423	75817	195216	5016	104951	5340
18.1q	59.78	1196.32	181.21	23.43	0.30	3685613	1230474	23038	47383	2749	51228	1587
18.1r	45.943	1196.22	191.22	23.53	0.33	1103901	2516321	55873	117534	5442	98732	3597
18.1s	53.071	1196.13	200.22	23.61	0.36	2976897	2034796	25502	42283	1570	25392	1814
18.1t	52.167	1196.08	205.21	23.66	0.38	1449020	1513825	24516	42895	1882	20818	1829
18.1u	46.584	1196.03	210.21	23.71	0.39	6457107	2104491	41711	55493	2646	30107	2744
18.1v1	58.903	1195.97	216.22	23.76	0.41	4392129	1477304	38318	54560	5698	59123	2919
18.1v2	57.119	1195.90	223.22	23.83	0.43	4096373	594712	48119	36445	23613	270174	12858
18.1v3	56.469	1195.83	230.21	23.90	0.46	21729588	504495	52986	45525	34119	308446	19189
18.1v4	51.141	1195.76	237.21	23.96	0.48	2449939	3736421	74755	158610	18432	228622	8624
18.1w	55.878	1195.69	244.22	24.03	0.51	207024	3204484	58599	136402	11647	129248	6406
18.1x1	52.239	1195.61	251.72	24.09	0.53	492602	954927	28769	51278	811	9840	590
18.1x2	54.514	1195.53	260.21	24.17	0.56	20833189	2727218	74207	113895	3646	37634	2357

Table S11 (cont'd)
Summary of GDGT data from WHSA Locality 2.

brGIII5	brGIII6	brGIII7	brGIII8	brGIIIb5	brGIIIb6	brGII5	brGII6	brGII7	brGIIb5	brGIIb6	brGIIc5	brGIIc6
22664	40616	56083	114434	0	48152	38606	126011	76782	0	186024	2636	61227
12841	21580	35064	89514	2220	10070	34788	82683	92350	31880	55125	2607	35553
5563	7416	11574	25262	1730	6214	17501	14805	43677	25431	23860	3267	8558
4909	7584	15348	32379	681	3721	14860	43769	36249	28469	23933	4296	3481
4061	6217	11994	9946	479	9844	6424	12242	73307	11287	20531	1092	6813
11531	9240	27079	57930	3564	7764	50580	76537	73008	82361	29842	11218	7390
18214	19457	52397	76545	3242	20930	35671	61315	130440	59048	92017	9199	16022
8790	10810	22245	26951	2076	2594	24743	27367	46361	28180	8084	5376	1933
15708	25016	53474	116419	5871	6082	43603	80630	112359	57705	15798	7031	3205
10030	5308	25083	48069	4408	3370	45017	62201	57920	63922	10974	9908	4872
64796	58264	115720	185949	27014	16184	224267	213887	302300	317582	56566	54966	21585
16472	19568	34976	44866	3105	3547	40385	31634	76982	35376	8564	5230	2125
17679	35657	44067	102610	5011	3947	37140	79606	100713	38827	9488	4918	1961
17961	26831	52843	70807	5942	2660	58315	52454	123157	68903	5228	9369	872
24658	39026	84985	178376	7235	3661	57733	113936	168896	59672	14578	5228	2409
29209	36699	64031	120905	15134	5164	105068	106057	175864	155387	16132	20929	10331
93239	128908	296216	434476	28018	16389	200108	203038	549321	165963	28018	20570	4601
38797	19614	71573	85326	13839	2805	89769	97182	122616	94693	9169	14705	2648
67429	51596	133840	144819	8516	4964	93041	56254	256798	55089	6892	9037	1398
26103	41838	123928	132502	7626	4322	61114	103628	226096	50081	8498	8143	1497
38761	63397	151693	225297	7975	8358	68487	138343	249224	52042	17720	6560	2558
68368	36072	237904	239326	26413	5236	134983	130489	412235	134360	13062	18095	4302
58171	52351	113920	126716	10386	4251	94566	86014	203687	65875	9764	9720	3424
31291	97623	70106	110601	14784	3119	89309	80511	211600	130196	11505	17308	5603
21861	39767	78889	156162	5700	5481	48406	133987	114999	39719	12641	4494	2384
26363	64012	73550	119507	3810	7210	55592	135838	129625	33946	27944	4374	3715
29191	63178	86625	173123	3289	6900	67798	242994	88179	29476	28635	3173	3740
44894	78628	131022	239120	12960	10391	104584	144845	349347	107483	30042	14386	4596
20579	45214	62156	173465	1719	4475	36292	149436	104886	18063	15921	2022	1683
86179	180141	95549	414303	11801	27139	142795	239935	312039	72102	96860	6779	17484
51377	98646	65027	405947	4685	7418	94772	168699	303877	63448	68696	2685	4820
46018	141823	85095	499498	5668	5237	90666	506717	186034	60287	31144	5810	2682
8777	27935	17834	94214	1613	1556	17453	36963	86353	13375	7547	1583	500
49885	89846	171543	359130	17094	8862	124022	373966	248037	120146	19519	14452	1997
15653	30416	76203	105338	3893	4431	34248	57558	131661	20729	7966	2702	1877
59561	120228	266925	487361	7539	11559	131399	400105	364089	69808	50624	3691	2657
22512	16900	87753	137378	3604	3609	39987	85431	137716	29760	5713	4088	780
30460	47852	82406	211638	5919	3965	64767	154489	167273	52973	15615	5463	1949
62306	77556	99248	159877	23086	9848	159962	181226	204798	174436	20378	23258	7047
42095	41632	100968	191641	7470	4736	80098	148014	134212	42663	9360	5223	1076
52940	103700	211500	398017	5692	9835	124267	386349	235595	53736	38375	5496	2934
14644	24513	68292	114631	3093	4023	32798	64682	117047	17945	10403	1983	1672
17260	27033	56896	129849	3118	2594	34778	103092	82471	20265	11512	1762	2001
121350	117445	290317	546409	64901	14638	283200	455669	608028	386677	41904	48857	5513
21653	23875	64767	115851	4623	4562	39084	91409	103243	34701	10933	3599	2490
134927	122720	207149	446037	60059	19669	317009	679302	197314	420016	73010	49461	61839
126072	110196	198358	220868	28701	5693	230839	116310	442706	164211	20553	28022	7058
159995	206047	192706	288196	38639	16772	287967	208320	562545	270970	63446	42666	42778
167704	526806	327290	493675	43232	36372	302855	661771	660735	335562	142014	49056	107153
80766	402123	283689	263644	23488	22054	123723	427751	529761	148901	76735	20219	43036
16214	70380	73902	93720	4728	6434	27597	112487	158029	43214	10171	6574	3336
11861	64873	88220	81544	6326	5042	32074	54760	207431	64533	8896	12788	2923
292169	877651	532967	1163434	123843	69832	427451	954627	1625657	735256	296553	100105	272303
146455	366704	321361	602044	49006	37227	221374	817121	439107	306507	114244	42777	78668
82039	357912	249229	461620	22887	10274	157902	322847	763707	171844	35916	18275	13204
31333	121506	136098	215041	10968	6180	50491	302743	146139	54756	14749	5365	4519

Table S11 (cont'd)
Summary of GDGT data from WHSA Locality 2.

brGI	brGIPr	brGIb	brGIc	mbt5me	BIT	MI	ir67	ACE	Temp	TempErr1sigma
584807	141726	105543	131458	0.928	0.839	0.321	0.699	0.854	12.963	2.939
550494	154159	80184	39857	0.888	0.864	0.228	0.648	0.650	12.898	2.917
223315	61729	84542	49725	0.870	0.825	0.233	0.586	0.868	13.593	2.729
365292	94578	72673	55138	0.903	0.683	0.185	0.575	0.115	14.889	3.008
90969	26793	24190	25934	0.858	0.730	0.248	0.736	0.292	10.044	2.780
575743	107980	238053	104025	0.852	0.773	0.197	0.484	0.560	15.048	2.734
591996	139891	148050	140653	0.875	0.744	0.241	0.642	0.075	12.698	2.714
218646	49887	50885	25903	0.810	0.906	0.268	0.497	0.219	13.482	2.703
495192	121234	76785	30338	0.823	0.878	0.241	0.566	0.263	12.709	2.851
367641	98705	147087	55624	0.811	0.879	0.257	0.455	0.118	14.459	2.805
1160634	331859	588659	234369	0.742	0.843	0.233	0.433	0.040	12.730	2.800
274927	75512	36627	21079	0.768	0.865	0.251	0.493	0.427	12.204	2.783
421023	90388	40947	17333	0.822	0.851	0.360	0.575	0.323	12.293	2.809
354820	62331	98444	50251	0.758	0.781	0.293	0.484	0.315	12.317	2.682
603969	166358	49243	15767	0.812	0.835	0.347	0.588	0.088	11.897	2.729
637861	207827	281978	115752	0.761	0.850	0.276	0.457	0.050	12.825	2.741
1518972	390176	133934	35057	0.769	0.848	0.376	0.550	0.114	11.169	2.893
473056	92915	100776	31145	0.706	0.846	0.373	0.438	0.069	11.753	2.728
603955	150545	43439	12740	0.739	0.847	0.335	0.504	0.591	11.234	2.885
644182	178121	41050	10698	0.820	0.938	0.373	0.593	0.041	11.948	2.897
802740	170546	40880	10780	0.831	0.936	0.490	0.620	0.288	11.730	2.837
973858	229191	98572	31656	0.743	0.916	0.429	0.519	0.039	11.092	2.776
524094	143337	55924	18460	0.715	0.881	0.387	0.500	0.056	10.642	2.785
514419	160115	176055	79730	0.731	0.897	0.363	0.490	0.287	11.388	2.767
542023	128290	33457	9456	0.830	0.830	0.324	0.604	0.244	11.991	2.813
539832	207544	37692	10291	0.826	0.969	0.571	0.596	0.407	12.151	2.915
721147	427022	28035	6503	0.850	0.983	0.715	0.593	0.550	12.843	2.934
1118903	291446	95232	25220	0.813	0.984	0.493	0.572	0.477	12.222	2.874
546515	360063	14512	4731	0.878	0.935	0.505	0.654	0.507	12.565	2.985
1339175	458861	68141	17234	0.817	0.958	0.730	0.598	0.806	11.677	2.860
1239918	444150	38661	7790	0.856	0.863	0.513	0.634	0.327	12.158	2.795
1767417	693189	44403	11673	0.897	0.930	0.627	0.631	0.770	13.603	2.851
337743	87840	13479	3883	0.892	0.870	0.477	0.660	0.922	12.665	2.912
1254941	297929	119574	40335	0.813	0.922	0.464	0.576	0.102	12.052	2.840
378628	118288	18539	5436	0.839	0.881	0.441	0.632	0.876	11.754	2.743
1533197	336692	64891	9884	0.855	0.950	0.654	0.647	0.854	12.012	2.978
461358	99125	30047	8098	0.833	0.945	0.645	0.609	0.250	11.930	2.791
684458	137056	59297	16162	0.826	0.925	0.592	0.596	0.678	12.337	2.800
722201	102082	253309	89106	0.706	0.935	0.468	0.446	0.800	11.492	2.724
611434	152606	43212	12016	0.790	0.950	0.615	0.557	0.108	11.908	2.687
1352456	359138	54832	12069	0.854	0.920	0.646	0.625	0.774	12.330	2.873
365051	66383	18467	5251	0.847	0.875	0.458	0.643	0.838	11.730	2.854
398211	66929	18608	4405	0.845	0.908	0.574	0.625	0.773	11.916	3.042
1855103	497107	292685	73960	0.711	0.964	0.715	0.504	0.031	10.429	2.766
413916	92432	27496	7229	0.812	0.920	0.581	0.590	0.750	11.537	2.880
1690539	524403	467429	142680	0.701	0.968	0.636	0.449	0.305	11.303	2.745
962259	241315	137662	45048	0.665	0.984	0.718	0.453	0.594	9.984	2.879
1403513	202411	327960	116481	0.698	0.991	0.754	0.475	0.489	10.451	2.779
2489675	129572	408274	157500	0.773	0.993	0.752	0.547	0.754	11.267	2.805
1706991	71376	180342	64487	0.831	0.979	0.614	0.610	0.748	11.967	2.762
634284	37453	55995	20135	0.878	0.761	0.277	0.632	0.873	12.756	2.837
730064	36407	105177	40047	0.873	0.743	0.288	0.607	0.977	13.178	3.034
4253859	293122	834365	335451	0.764	0.967	0.515	0.581	0.396	10.598	2.730
2489586	149858	335641	117576	0.793	0.969	0.604	0.558	0.061	11.848	2.661
2349940	121290	157856	44240	0.849	0.997	0.886	0.618	0.340	12.182	2.921
949645	65536	56678	14458	0.870	0.975	0.827	0.621	0.884	12.723	3.009

Table S11 (cont'd)
Summary of GDGT data from WHSA Locality 2.

Lake_prob	arineSalty_pro	Peat_prob	Soil_prob	Best_pred
0.04	0.03	0.03	0.91	Soil-type
0.07	0.05	0.02	0.86	Soil-type
0.16	0.13	0.09	0.62	Soil-type
0.20	0.07	0.02	0.71	Soil-type
0.20	0.12	0.06	0.62	Soil-type
0.15	0.09	0.04	0.72	Soil-type
0.13	0.12	0.06	0.68	Soil-type
0.28	0.17	0.07	0.48	Soil-type
0.32	0.15	0.06	0.47	Soil-type
0.27	0.11	0.08	0.54	Soil-type
0.30	0.15	0.08	0.48	Soil-type
0.35	0.20	0.07	0.38	Soil-type
0.35	0.25	0.07	0.33	Lake-type
0.29	0.23	0.12	0.36	Soil-type
0.41	0.28	0.08	0.24	Lake-type
0.37	0.17	0.09	0.37	Lake-type
0.39	0.31	0.11	0.18	Lake-type
0.53	0.16	0.10	0.20	Lake-type
0.45	0.28	0.14	0.13	Lake-type
0.56	0.16	0.11	0.17	Lake-type
0.54	0.22	0.10	0.14	Lake-type
0.50	0.17	0.17	0.16	Lake-type
0.58	0.19	0.09	0.14	Lake-type
0.46	0.21	0.13	0.20	Lake-type
0.51	0.24	0.07	0.17	Lake-type
0.72	0.09	0.06	0.14	Lake-type
0.67	0.09	0.07	0.16	Lake-type
0.76	0.05	0.10	0.10	Lake-type
0.40	0.29	0.09	0.22	Lake-type
0.59	0.19	0.08	0.15	Lake-type
0.19	0.53	0.09	0.19	Marine-type
0.29	0.39	0.12	0.20	Marine-type
0.19	0.53	0.12	0.17	Marine-type
0.46	0.20	0.10	0.24	Lake-type
0.29	0.44	0.09	0.18	Marine-type
0.44	0.21	0.14	0.20	Lake-type
0.53	0.13	0.19	0.15	Lake-type
0.49	0.21	0.11	0.19	Lake-type
0.55	0.15	0.11	0.19	Lake-type
0.64	0.13	0.11	0.12	Lake-type
0.27	0.41	0.13	0.20	Marine-type
0.20	0.52	0.09	0.19	Marine-type
0.38	0.29	0.15	0.18	Lake-type
0.68	0.07	0.15	0.10	Lake-type
0.55	0.19	0.12	0.14	Lake-type
0.68	0.07	0.13	0.11	Lake-type
0.71	0.07	0.17	0.05	Lake-type
0.73	0.08	0.14	0.06	Lake-type
0.72	0.07	0.12	0.09	Lake-type
0.73	0.07	0.09	0.10	Lake-type
0.33	0.28	0.07	0.31	Lake-type
0.24	0.31	0.10	0.35	Soil-type
0.66	0.09	0.12	0.13	Lake-type
0.72	0.09	0.08	0.11	Lake-type
0.77	0.05	0.10	0.08	Lake-type
0.67	0.12	0.10	0.11	Lake-type

Table S12

Summary of leaf wax isotope data from WHSA Locality 2.

LayerID	Elevation	Thickness	Depth	Age	AgeError	C28AvgdD	C30AvgdD	C28SEdD	C30SEdD	dAvgC28C30	dAvgSE	dDprecip
15.1a	1198.13	4.00	0.00	21.64	0.26	-161.7	-154.5	1.7	1.5	-158.1	1.6	-64.7
15.1b	1198.08	5.00	4.50	21.68	0.24	-169.3	-164.0	0.2	0.4	-166.6	0.3	-74.2
15.1c	1198.03	5.00	9.50	21.73	0.22	-168.2	-167.2	1.1	1.0	-167.7	1.0	-75.3
15.1d	1197.98	5.00	14.50	21.78	0.21	-173.1	-171.4	0.2	0.3	-172.2	0.3	-80.4
15.1e	1197.94	4.00	19.00	21.83	0.19	-168.4	-162.4	0.3	0.8	-165.4	0.6	-72.8
15.1f	1197.90	3.00	22.50	21.86	0.18	-177.0	-168.7	0.7	1.0	-172.9	0.8	-81.1
15.1g	1197.87	4.00	26.00	21.90	0.17	-178.7	-171.9	0.4	1.0	-175.3	0.7	-83.8
15.1h	1197.83	3.00	29.50	21.93	0.15	-163.1	-153.1	0.7	0.7	-158.1	0.7	-64.7
15.1i	1197.79	5.00	33.50	21.97	0.14	-168.1	-163.7	0.6	0.7	-165.9	0.6	-73.4
15.1j	1197.76	2.00	37.00	22.01	0.12	-167.0	-163.9	1.8	1.1	-165.5	1.5	-72.9
15.1k	1197.72	6.00	41.00	22.07	0.10	-179.0	-175.7	0.6	0.8	-177.4	0.7	-86.1
15.1l	1197.67	3.00	45.50	22.13	0.10	-168.0	-163.7	1.0	1.2	-165.8	1.1	-73.3
15.1m	1197.64	4.00	49.00	22.18	0.09	-165.8	-163.4	0.4	1.0	-164.6	0.8	-71.9
15.1n	1197.61	2.00	52.00	22.21	0.09	-173.4	-167.6	1.5	1.6	-170.5	1.6	-78.5
15.1o	1197.58	4.00	55.00	22.24	0.09	-170.4	-165.1	0.5	0.5	-167.8	0.5	-75.5
15.1p	1197.55	2.00	58.00	22.27	0.08	-177.8	-174.2	0.8	0.3	-176.0	0.6	-84.6
15.1q	1197.51	5.00	61.50	22.31	0.08	-172.7	-168.3	0.8	1.1	-170.5	1.0	-78.5
15.1r	1197.46	5.00	66.50	22.36	0.07	-171.2	-165.8	0.9	1.3	-168.5	1.1	-76.3
15.1s	1197.42	4.00	71.00	22.39	0.06	-158.7	-163.3	0.3	1.1	-161.0	0.8	-68.0
15.1t	1197.39	1.88	73.94	22.41	0.06	-162.8	-165.7	0.3	1.7	-164.2	1.2	-71.5
15.1u	1197.37	1.88	75.81	22.42	0.06	-166.3	-165.3	0.4	1.7	-165.8	1.2	-73.3
15.1v	1197.35	3.05	78.28	22.44	0.06	-171.3	-161.7	0.2	2.0	-166.5	1.4	-74.1
15.1w	1197.32	3.05	81.33	22.48	0.06	-163.5	-168.0	0.4	1.1	-165.8	0.8	-73.2
15.1x	1197.28	3.25	84.48	22.51	0.06	-168.4	-161.9	1.0	1.6	-165.1	1.3	-72.5
15.1y	1197.24	4.75	88.48	22.55	0.07	-160.8	-165.7	1.3	1.3	-163.2	1.3	-70.4
15.1z	1197.20	3.55	92.63	22.59	0.08	-170.2	-164.7	1.1	1.2	-167.5	1.1	-75.1
17.1g	1197.17	2.54	95.67	22.62	0.09	-168.0	-163.9	0.7	1.0	-165.9	0.8	-73.4
17.1h	1197.15	2.28	98.08	22.65	0.09	-171.7	-171.6	2.3	1.9	-171.7	2.1	-79.8
17.1i	1197.12	2.75	100.60	22.67	0.10	-164.1	-158.8	1.4	1.1	-161.5	1.3	-68.5
17.1j	1197.09	3.74	103.85	22.70	0.11	-159.9	-158.1	0.8	1.4	-159.0	1.1	-65.7
18.1c	1197.08	1.87	104.78	22.71	0.11	-167.7	-162.6	0.6	1.7	-165.2	1.3	-72.6
18.1d	1197.06	3.00	107.21	22.73	0.12	-171.1	-169.3	1.6	1.3	-170.2	1.5	-78.2
18.1e	1197.01	6.00	111.72	22.77	0.13	-166.9	-163.7	1.4	1.9	-165.3	1.6	-72.7
18.1f	1196.96	4.00	116.71	22.82	0.14	-168.1	-164.8	0.3	0.6	-166.4	0.5	-74.0
18.1g	1196.91	6.00	121.71	22.86	0.15	-163.9	-160.2	1.3	0.3	-162.0	0.9	-69.1
18.1h	1196.86	4.00	126.72	22.91	0.16	-165.6	-159.3	1.0	1.4	-162.5	1.2	-69.6
18.1i	1196.82	4.00	130.72	22.95	0.17	-159.1	-162.1	0.7	1.6	-160.6	1.2	-67.5
18.1j	1196.77	6.00	135.72	22.99	0.18	-167.0	-160.3	0.4	0.8	-163.7	0.6	-70.9
18.1k	1196.72	5.00	141.22	23.05	0.20	-178.3	-173.7	1.6	2.1	-176.0	1.9	-84.6
18.1l	1196.65	9.00	148.22	23.11	0.21	-166.1	-161.3	0.7	0.5	-163.7	0.6	-71.0
18.1m	1196.56	8.00	156.72	23.19	0.23	-154.2	-150.1	0.9	0.9	-152.2	0.9	-58.1
18.1n	1196.50	5.00	163.22	23.25	0.25	-153.4	-149.3	0.9	0.3	-151.4	0.7	-57.2
18.1o	1196.45	5.00	168.22	23.30	0.26	-161.4	-157.1	0.6	0.7	-159.2	0.7	-66.0
18.1p	1196.39	7.00	174.22	23.36	0.28	-172.5	-170.4	2.4	0.9	-171.4	1.8	-79.5
18.1q	1196.32	7.00	181.21	23.43	0.30	-164.7	-161.2	1.4	1.4	-162.9	1.4	-70.1
18.1r	1196.22	13.00	191.22	23.53	0.33	-172.6	-168.3	0.6	1.5	-170.4	1.1	-78.4
18.1s	1196.13	5.00	200.22	23.61	0.36	-164.4	-165.2	1.3	1.5	-164.8	1.4	-72.2
18.1t	1196.08	5.00	205.21	23.66	0.38	-170.1	-166.5	1.1	2.3	-168.3	1.8	-76.0
18.1u	1196.03	5.00	210.21	23.71	0.39	-174.7	-162.8	0.2	1.7	-168.7	1.2	-76.5
18.1v1	1195.97	7.00	216.22	23.76	0.41	-174.6	-183.4	0.7	1.7	-179.0	1.3	-87.9
18.1v2	1195.90	7.00	223.22	23.83	0.43	-175.2	-174.4	0.5	0.2	-174.8	0.4	-83.3
18.1v3	1195.83	7.00	230.21	23.90	0.46	-164.4	-168.4	0.4	0.5	-166.4	0.4	-73.9
18.1v4	1195.76	7.00	237.21	23.96	0.48	-172.4	-174.2	1.3	1.2	-173.3	1.2	-81.6
18.1w	1195.69	7.00	244.22	24.03	0.51	-173.2	-173.6	0.4	0.6	-173.4	0.5	-81.7
18.1x1	1195.61	8.00	251.72	24.09	0.53	-178.2	-178.1	1.3	0.9	-178.1	1.1	-87.0
18.1x2	1195.53	9.00	260.21	24.17	0.56	-171.3	-170.5	1.1	0.8	-170.9	0.9	-78.9

Table S13

Summary of pollen counts for samples collected from the extended trench at WHSA Locality 2.

Sample #	Elevation (masl)	Modeled age (ka)	Indeterminable	*Pinus* indeterminate	*Pinus diploxylon*	*Pinus haploxylon*	*Piñon*	*Artemisia*	Cheno-Am	Poaceae	Asteraceae
22WHSA1-15.1b	1198.08	21.68 ± 0.25	1	6	19	0	4	101	10	20	15
22WHSA1-15.1d	1197.98	21.78 ± 0.22	1	10	17	1	2	66	15	21	18
22WHSA1-15.1f	1197.90	21.86 ± 0.19	1	3	11	1	3	79	14	38	24
22WHSA1-15.1g	1197.87	21.89 ± 0.17	6	12	3	0	0	81	11	33	23
22WHSA1-15.1j	1197.76	22.01 ± 0.13	2	15	26	1	2	45	7	14	9
22WHSA1-15.1k	1197.72	22.07 ± 0.10	2	14	31	4	3	78	8	12	19
22WHSA1-15.1m	1197.64	22.18 ± 0.09	0	4	29	4	4	75	4	23	17
22WHSA1-15.1r	1197.46	22.36 ± 0.07	1	1	45	2	12	81	8	17	17
20WHSA1-18.2aa	1197.15	22.65 ± 0.09	0	4	56	2	7	73	9	12	21
22WHSA1-17.1j	1197.09	22.69 ± 0.11	1	5	52	10	9	61	3	26	16
22WHSA1-18.1f	1196.96	22.81 ± 0.14	0	2	33	5	13	64	6	13	14
22WHSA1-18.1k	1196.72	23.03 ± 0.19	2	7	25	5	15	54	7	38	8
22WHSA1-18.1m	1196.56	23.18 ± 0.23	0	7	35	12	9	58	6	20	9
22WHSA1-18.1n	1196.50	23.25 ± 0.25	1	5	72	2	57	52	10	20	2
22WHSA1-18.1q	1196.32	23.43 ± 0.30	1	7	49	2	20	49	1	15	10
22WHSA1-18.1r	1196.22	23.52 ± 0.33	1	4	54	18	4	66	1	25	8
22WHSA1-18.1s	1196.13	23.61 ± 0.37	0	5	66	16	11	49	2	27	10
22WHSA1-18.1u	1196.03	23.71 ± 0.40	2	14	53	21	3	51	3	17	15
22WHSA1-18.1w	1195.69	24.04 ± 0.52	1	5	40	19	8	50	4	20	14
22WHSA1-18.1x2	1195.53	24.19 ± 0.57	0	10	42	0	36	60	4	21	15

Table S13 (cont'd)

Sample #	*Ambrosia*	*Juniperus*	*Quercus*	*Abies*	*Picea*	*Pseudotsuga*	*Populus*	*Sarcobatus*	*Ephedra*	Lactuceae	*Cercocarpus*	Rosaceae
22WHSA1-15.1b	13	11	6	0	6	1	0	1	0	0	0	0
22WHSA1-15.1d	4	1	15	0	2	0	1	0	0	0	0	0
22WHSA1-15.1f	6	15	14	0	0	2	0	0	0	0	0	0
22WHSA1-15.1g	4	24	9	0	1	1	0	1	0	0	1	0
22WHSA1-15.1j	6	22	3	1	3	0	0	0	0	0	2	0
22WHSA1-15.1k	0	19	5	0	4	1	0	2	0	0	0	0
22WHSA1-15.1m	6	32	3	0	3	0	0	2	1	0	0	0
22WHSA1-15.1r	2	26	2	0	8	2	0	0	1	0	0	1
20WHSA1-18.2aa	2	22	0	1	7	1	0	0	0	0	0	0
22WHSA1-17.1j	3	21	1	0	4	0	1	0	0	0	0	0
22WHSA1-18.1f	4	33	2	0	6	1	0	1	0	0	0	1
22WHSA1-18.1k	5	27	2	0	3	1	0	1	0	0	0	1
22WHSA1-18.1m	6	27	3	0	4	0	0	0	0	0	0	1
22WHSA1-18.1n	8	27	4	0	6	0	0	2	0	0	0	0
22WHSA1-18.1q	0	29	1	0	5	0	0	1	0	0	0	0
22WHSA1-18.1r	1	22	0	1	4	1	1	2	0	0	0	0
22WHSA1-18.1s	2	19	1	1	5	0	0	0	0	0	0	0
22WHSA1-18.1u	1	25	0	0	1	0	0	1	0	0	0	0
22WHSA1-18.1w	2	28	2	0	6	0	0	2	0	0	1	0
22WHSA1-18.1x2	3	12	0	1	3	0	0	1	0	0	0	0

Table S13 (cont'd)

Sample #	Lamiaceae	Apiaceae	Liliaceae	Caryophyllaceae	Polemoniaceae	*Shepherdia*	*Yucca*	Campanulaceae	Ericaceae	Urticaceae	*Typha*	*Polygonum*
22WHSA1-15.1b	0	0	0	0	0	0	0	0	0	0	0	0
22WHSA1-15.1d	0	0	0	0	0	0	0	0	0	0	0	0
22WHSA1-15.1f	1	0	0	0	0	0	0	0	0	0	0	0
22WHSA1-15.1g	0	0	0	0	0	0	0	0	0	0	0	0
22WHSA1-15.1j	0	0	0	0	0	0	1	0	0	0	0	0
22WHSA1-15.1k	0	0	0	0	0	0	0	1	0	0	0	0
22WHSA1-15.1m	0	0	0	0	0	0	0	0	0	0	1	0
22WHSA1-15.1r	0	1	0	0	0	0	0	0	0	0	0	0
20WHSA1-18.2aa	0	0	0	0	0	0	0	0	0	0	0	0
22WHSA1-17.1j	0	0	0	0	0	0	0	0	0	0	0	0
22WHSA1-18.1f	0	0	0	0	0	0	0	0	0	0	0	0
22WHSA1-18.1k	0	0	0	0	0	0	0	0	0	0	0	0
22WHSA1-18.1m	0	0	0	0	0	0	0	0	0	0	0	0
22WHSA1-18.1n	0	0	0	0	1	0	0	0	0	0	0	0
22WHSA1-18.1q	0	0	0	0	0	1	0	0	0	0	0	0
22WHSA1-18.1r	0	0	0	0	0	0	0	0	0	0	0	0
22WHSA1-18.1s	0	0	0	0	0	0	0	0	0	0	0	0
22WHSA1-18.1u	0	0	0	0	0	0	0	0	0	0	0	0
22WHSA1-18.1w	0	0	0	0	0	0	0	0	0	0	0	0
22WHSA1-18.1x2	0	0	0	0	0	0	0	0	0	0	0	0

Table S13 (cont'd)

Sample #	*Lonicera*	*Juglans*	*Salix*	*Alnus*	*Celtis*	Cyperaceae	*Thalictrum*	*Plantago*	Ranunculaceae	*Ruppia*
22WHSA1-15.1b	0	0	0	0	0	0	0	0	4	0
22WHSA1-15.1d	0	0	0	0	1	0	0	0	4	3
22WHSA1-15.1f	0	0	0	0	0	3	0	0	0	151
22WHSA1-15.1g	0	1	0	0	0	1	0	0	0	237
22WHSA1-15.1j	0	0	0	0	0	4	0	0	0	70
22WHSA1-15.1k	0	0	0	0	0	0	0	0	0	147
22WHSA1-15.1m	0	0	0	0	0	2	0	0	0	33
22WHSA1-15.1r	0	0	0	0	0	2	0	0	0	115
20WHSA1-18.2aa	0	0	0	0	0	3	0	0	0	135
22WHSA1-17.1j	0	0	0	0	0	0	0	0	0	0
22WHSA1-18.1f	0	0	0	0	0	6	0	0	0	155
22WHSA1-18.1k	0	0	0	0	0	0	0	0	0	324
22WHSA1-18.1m	0	0	0	0	0	1	0	0	0	0
22WHSA1-18.1n	0	0	0	0	0	2	0	0	0	0
22WHSA1-18.1q	0	0	0	0	0	3	0	0	0	2
22WHSA1-18.1r	0	0	0	0	0	0	0	0	0	20
22WHSA1-18.1s	0	0	0	0	0	1	0	0	0	137
22WHSA1-18.1u	0	0	0	0	0	2	0	0	0	6
22WHSA1-18.1w	0	0	0	0	0	3	1	0	0	16
22WHSA1-18.1x2	0	0	0	0	0	2	0	0	0	1

Table S14

Summary of PCA matrix data.

Elevation	Depth	Age	ir67	ACE	d2Hp	Temp	LakeGDGTs	SoilGDGTs	SaltyGDGTs
1198.08	4.50	21.68	0.6483	0.6502	-74.2123	12.8977	0.0733	0.8610	0.0507
1197.98	14.50	21.78	0.5746	0.1155	-80.3897	14.8888	0.1967	0.7117	0.0700
1197.90	22.50	21.86	0.4837	0.5603	-81.1234	15.0478	0.1530	0.7200	0.0903
1197.87	26.00	21.90	0.6421	0.0745	-83.8195	12.6982	0.1317	0.6820	0.1243
1197.76	37.00	22.01	0.4555	0.1179	-72.8970	14.4588	0.2743	0.5377	0.1097
1197.72	41.00	22.07	0.4328	0.0401	-86.1075	12.7304	0.3010	0.4767	0.1450
1197.64	49.00	22.18	0.5752	0.3228	-71.9428	12.2930	0.3523	0.3260	0.2547
1197.46	66.50	22.36	0.4377	0.0693	-76.2873	11.7529	0.5337	0.2027	0.1613
1197.15	98.08	22.65	0.5722	0.4767	-79.8153	12.2224	0.7583	0.0980	0.0480
1197.09	103.85	22.70	0.5980	0.8064	-65.6861	11.6775	0.5907	0.1450	0.1883
1196.96	116.71	22.82	0.5763	0.1022	-73.9743	12.0523	0.4590	0.2423	0.2020
1196.72	141.22	23.05	0.4457	0.8005	-84.6323	11.4924	0.5533	0.1923	0.1463
1196.56	156.72	23.19	0.6248	0.7741	-58.1099	12.3304	0.2663	0.2013	0.4073
1196.50	163.22	23.25	0.6431	0.8382	-57.2339	11.7302	0.2030	0.1943	0.5177
1196.32	181.21	23.43	0.5899	0.7497	-70.0733	11.5369	0.5520	0.1433	0.1890
1196.22	191.22	23.53	0.4489	0.3049	-78.4233	11.3028	0.6803	0.1143	0.0720
1196.13	200.22	23.61	0.4533	0.5940	-72.1649	9.9835	0.7053	0.0533	0.0710
1196.03	210.21	23.71	0.5465	0.7542	-76.5445	11.2666	0.7163	0.0930	0.0740
1195.69	244.22	24.03	0.5581	0.0607	-81.7262	11.8481	0.7220	0.1080	0.0867
1195.53	260.21	24.17	0.6209	0.8842	-78.9346	12.7228	0.6713	0.1100	0.1200

Table S14 (cont'd)

Summary of PCA matrix data.

Pinus	Piñon	Artemisia	ChenoAm	Poaceae	Asteraceae	Ambrosia	Juniper	Oak	SpruceFir	Ruppia
11.7925	1.8868	47.6415	4.7170	9.4340	7.0755	6.1321	5.1887	2.8302	3.3019	0.0000
16.2791	1.1628	38.3721	8.7209	12.2093	10.4651	2.3256	0.5814	8.7209	1.1628	1.6484
7.1429	1.4286	37.6190	6.6667	18.0952	11.4286	2.8571	7.1429	6.6667	0.9524	41.2568
7.4257	0.0000	40.0990	5.4455	16.3366	11.3861	1.9802	11.8812	4.4554	0.9901	52.7840
27.2727	1.2987	29.2208	4.5455	9.0909	5.8442	3.8961	14.2857	1.9481	2.5974	30.0429
24.7475	1.5152	39.3939	4.0404	6.0606	9.5960	0.0000	9.5960	2.5253	2.5253	42.0000
18.1373	1.9608	36.7647	1.9608	11.2745	8.3333	2.9412	15.6863	1.4706	1.4706	13.5802
21.5247	5.3812	36.3229	3.5874	7.6233	7.6233	0.8969	11.6592	0.8969	4.4843	33.4302
28.5714	3.2258	33.6406	4.1475	5.5300	9.6774	0.9217	10.1382	0.0000	4.1475	38.0282
31.7536	4.2654	28.9100	1.4218	12.3223	7.5829	1.4218	9.9526	0.4739	1.8957	0.0000
20.4082	6.6327	32.6531	3.0612	6.6327	7.1429	2.0408	16.8367	1.0204	3.5714	43.1755
18.7817	7.6142	27.4112	3.5533	19.2893	4.0609	2.5381	13.7056	1.0152	2.0305	61.7143
27.5510	4.5918	29.5918	3.0612	10.2041	4.5918	3.0612	13.7755	1.5306	2.0408	0.0000
29.8113	21.5094	19.6226	3.7736	7.5472	0.7547	3.0189	10.1887	1.5094	2.2642	0.0000
30.8511	10.6383	26.0638	0.5319	7.9787	5.3191	0.0000	15.4255	0.5319	2.6596	1.0204
36.3636	1.9139	31.5789	0.4785	11.9617	3.8278	0.4785	10.5263	0.0000	2.8708	8.5837
40.6542	5.1402	22.8972	0.9346	12.6168	4.6729	0.9346	8.8785	0.4673	2.8037	38.9205
43.1373	1.4706	25.0000	1.4706	8.3333	7.3529	0.4902	12.2549	0.0000	0.4902	2.7907
32.3232	4.0404	25.2525	2.0202	10.1010	7.0707	1.0101	14.1414	1.0101	3.0303	7.2072
25.1208	17.3913	28.9855	1.9324	10.1449	7.2464	1.4493	5.7971	0.0000	1.9324	0.4739

Table S14 (cont'd)

Summary of PCA loading data.

Variable	PC1Temp	PC2Aridity
ir67	0.14	0.34
ACE	-0.05	0.32
d2Hp	-0.02	0.44
Temp	0.33	-0.02
LakeGDGTs	-0.36	-0.17
SoilGDGTs	0.37	-0.03
SaltyGDGTs	0.02	0.41
Pinus	-0.33	0.03
Piñon	-0.08	0.33
Artemisia	0.26	-0.17
ChenoAm	0.34	-0.04
Poaceae	0.13	-0.06
Asteraceae	0.18	-0.29
Ambrosia	0.28	0.23
Juniper	-0.20	-0.01
Oak	0.34	-0.03
SpruceFir	-0.16	-0.10
Ruppia	-0.01	-0.32

Table S14 (cont'd)

Summary of PCA time series data.

Elevation	Depth	Age	PC1Temp	PC2Aridity
1198.08	4.50	21.68	3.9949	0.7918
1197.98	14.50	21.78	5.2522	-1.2278
1197.90	22.50	21.86	4.8403	-1.7480
1197.87	26.00	21.90	3.6955	-1.8955
1197.76	37.00	22.01	1.1427	-0.5975
1197.72	41.00	22.07	0.6793	-2.7322
1197.64	49.00	22.18	0.6251	0.4695
1197.46	66.50	22.36	-1.0266	-1.6193
1197.15	98.08	22.65	-1.3916	-1.6014
1197.09	103.85	22.70	-1.3188	1.4348
1196.96	116.71	22.82	-0.6755	-0.3197
1196.72	141.22	23.05	-0.7523	-0.7712
1196.56	156.72	23.19	-0.0649	3.4269
1196.50	163.22	23.25	-0.8840	5.7189
1196.32	181.21	23.43	-2.5495	1.5774
1196.22	191.22	23.53	-2.6513	-1.0180
1196.13	200.22	23.61	-3.2983	-0.4223
1196.03	210.21	23.71	-2.5589	0.0891
1195.69	244.22	24.03	-2.0238	-0.9610
1195.53	260.21	24.17	-1.0343	1.4054

Table S15
Summary of ostracode data (in valves per gram) for samples collected from the extended trench at WHSA Locality 2.

Sample #	Elevation (masl)	Modeled age (ka)	*Limnocythere staplini*	*Limnocythere staplini* (juvenile)	*Cyprideis salebrosa*	*Cyprideis salebrosa* (juvenile)	*Criboelphidium* sp. cf. *gunteri*	Chara oogonia
22WHSA1-15.1f	1197.90	21.86 ± 0.19	0	0	0	0	0	0
22WHSA1-15.1m	1197.64	22.18 ± 0.09	0	0	0	0	0	0
22WHSA1-17.1j	1197.09	22.69 ± 0.11	0	0	0	0	0	0
22WHSA1-18.1p	1196.39	23.36 ± 0.28	8	2	0	0	0	0
22WHSA1-18.1r	1196.22	23.52 ± 0.33	55	11	0	0	0	0
22WHSA1-18.1s	1196.13	23.61 ± 0.37	0	0	0	0	0	0
22WHSA1-18.1t	1196.08	23.66 ± 0.38	442	23	8	6	189	6
22WHSA1-18.1u	1196.03	23.71 ± 0.40	443	38	6	8	205	14
22WHSA1-18.1v1	1195.97	23.77 ± 0.42	0	0	0	0	0	0
22WHSA1-18.1v2	1195.90	23.83 ± 0.44	175	5	0	1	4	1
22WHSA1-18.1v3	1195.83	23.90 ± 0.47	0	0	0	0	0	0
22WHSA1-18.1v4	1195.76	23.97 ± 0.49	337	21	0	0	120	10
22WHSA1-18.1w	1195.69	24.04 ± 0.52	118	11	0	0	0	0
22WHSA1-18.1x1	1195.61	24.11 ± 0.54	8	0	0	0	0	0
22WHSA1-18.1x2	1195.53	24.19 ± 0.57	0	0	0	0	0	0

REFERENCES

1. M. R. Bennett, D. Bustos, J. S. Pigati, K. B. Springer, T. M. Urban, V. T. Holliday, S. C. Reynolds, M. Budka, J. S. Honke, A. M. Hudson, B. Fenerty, C. Connelly, P. J. Martinez, V. L. Santucci, D. Odess, Evidence of humans in North America during the Last Glacial Maximum. *Science* **373**, 1528–1531 (2021).

2. M. R. Bennett, T. M. Urban, D. Bustos, S. C. Reynolds, E. A. Jolie, H. C. Strehlau, D. Odess, K. B. Springer, J. S. Pigati, The ichnology of White Sands (New Mexico): Linear traces and human footprints, evidence of transport technology? *Quat. Sci. Adv.* **17**, 100274 (2025).

3. J. S. Pigati, K. B. Springer, J. S. Honke, D. B. Wahl, M. R. Champagne, S. R. H. Zimmerman, H. J. Gray, V. L. Santucci, D. Odess, D. Bustos, M. R. Bennett, Independent age estimates resolve the controversy of ancient human footprints at White Sands. *Science* **382**, 73–75 (2023).

4. P. U. Clark, A. S. Dyke, J. D. Shakun, A. E. Carlson, J. Clark, B. Wohlfarth, J. X. Mitrovica, S. W. Hostetler, A. M. McCabe, The Last Glacial Maximum. *Science* **325**, 710–714 (2009).

5. D. B. Madsen, L. G. Davis, D. Rhode, C. G. Oviatt, Comment on "Evidence of humans in North America during the Last Glacial Maximum". *Science* **375**, eabm4678 (2022).

6. C. G. Oviatt, D. B. Madsen, D. Rhode, L. G. Davis, A critical assessment of claims that human footprints in the Lake Otero basin, New Mexico date to the Last Glacial Maximum. *Quatern. Res.* **111**, 138–147 (2022).

7. C. V. Haynes Jr., Evidence for humans at White Sands National Park during the Last Glacial Maximum could actually be for Clovis people ∼13,000 years ago. *PaleoAmerica* **8**, 95–98 (2022).

8. D. M. Rachal, K. Zeigler, R. Dello-Russo, C. Solfisburg, Lake levels and trackways: An alternative model to explain the timing of human-megafauna trackway intersections, Tularosa Basin, New Mexico. *Quat. Sci. Adv.* **3**, 100024 (2021).

9. D. M. Rachal, J. I. Mead, R. Dello-Russo, M. T. Cuba, Deep-water delivery model of *Ruppia* seeds to a nearshore/terrestrial setting and its chronological implications for Late Pleistocene footprints, Tularosa Basin, New Mexico. *Geoarchaeology* **37**, 922–933 (2022).

10. D. M. Rachal, R. Dello-Russo, M. Cuba, The Pleistocene footprints are younger than we thought: Correcting the radiocarbon dates of *Ruppia* seeds, Tularosa Basin, New Mexico. *Quat. Res.* **117**, 67–78 (2024).

11. D. Rhode, C. M. Neudorf, D. Rachal, L. G. Davis, D. B. Madsen, R. Dello-Russo, Unresolved: Persistent problems with the White Sands Locality 2 geochronology. *PaleoAmerica* **10**, 10–27 (2024).

12. V. T. Holliday, J. D. Windingstad, J. Bright, B. G. Phillips, J. B. Butler, R. Breslawski, J. E. Bowman, Paleolake geochronology supports Last Glacial Maximum (LGM) age for human tracks at White Sands, New Mexico. *Sci. Adv.* **11**, eadv4951 (2025).

13. J. S. Pigati, K. B. Springer, M. R. Bennett, D. Bustos, T. M. Urban, V. T. Holliday, S. C. Reynolds, D. Odess, Response to Comment on “Evidence of humans in North America during the Last Glacial Maximum”. *Science* **375**, eabm6987 (2022).

14. J. S. Pigati, K. B. Springer, H. J. Gray, D. Bustos, M. R. Bennett, The geochronology of White Sands Locality 2 is resolved. *PaleoAmerica* **10**, 28–44 (2024).

15. J. S. Pigati, K. B. Springer, V. T. Holliday, M. R. Bennett, D. Bustos, T. M. Urban, S. C. Reynolds, D. Odess, Reply to “Evidence for humans at White Sands National Park during the Last Glacial Maximum could actually be for Clovis People ∼13,000 years ago” by C. Vance Haynes, Jr. *PaleoAmerica* **8**, 99–101 (2022).

16. S. A. Mahan, H. J. Gray, J. S. Pigati, J. Wilson, N. A. Lifton, J. B. Paces, M. Blaauw, A geochronologic framework for the Ziegler Reservoir fossil site, Snowmass Village, Colorado. *Quat. Res.* **82**, 490–503 (2014).

17. E. M. Keaveney, P. J. Reimer, Understanding the variability in freshwater radiocarbon reservoir offsets: A cautionary tale. *J. Archaeol. Sci.* **39**, 1306–1316 (2012).

18. I. L. Hendy, J. P. Kennett, E. B. Roark, B. L. Ingram, Apparent synchroneity of submillennial scale climate events between Greenland and Santa Barbara Basin, California from 30–10ka. *Quat. Sci. Rev.* **21**, 1167–1184 (2002).

19. J. L. Oster, N. P. Kelley, Tracking regional and global teleconnections recorded by western North American speleothem records. *Quat. Sci. Rev.* **149**, 18–33 (2016).

20. K. B. Springer, C. R. Manker, J. S. Pigati, Dynamic response of desert wetlands to abrupt climate change. *Proc. Natl. Acad. Sci. U.S.A.* **112**, 14522–14526 (2015).

21. K. K. Andersen, A. Svensson, S. J. Johnsen, S. O. Rasmussen, M. Bigler, R. Röthlisberger, U. Ruth, M.-L. Siggaard-Andersen, J. P. Steffensen, D. Dahl-Jensen, B. M. Vinther, H. B. Clausen, The Greenland Ice Core Chronology 2005, 15–42 ka. Part 1: Constructing the time scale: Constructing the time scale. *Quat. Sci. Rev.* **25**, 3246–3257 (2006).

22. W. Ebisuzaki, A method to estimate the statistical significance of a correlation when the data are serially correlated. *J. Climate* **10**, 2147–2153 (1997).

23. D. Sachse, I. Billault, G. J. Bowen, Y. Chikaraishi, T. E. Dawson, S. J. Feakins, K. H. Freeman, C. R. Magill, F. A. McInerney, M. T. J. van der Meer, P. Polissar, R. J. Robins, J. P. Sachs, H. L. Schmidt, A. L. Sessions, J. W. C. White, J. B. West, A. Kahmen, Molecular paleohydrology: Interpreting the hydrogen-isotopic composition of lipid biomarkers from photosynthesizing organisms. *Annu. Rev. Earth Planet. Sci.* **40**, 221–249 (2012).

24. E. D. Crampton-Flood, J. E. Tierney, F. Peterse, F. M. Kirkels, J. S. S. Damsté, BayMBT: A Bayesian calibration model for branched glycerol dialkyl glycerol tetraethers in soils and peats. *Geochim. Cosmochim. Acta* **268**, 142–159 (2020).

25. P. Martínez-Sosa, J. E. Tierney, I. C. Stefanescu, E. D. Crampton-Flood, B. N. Shuman, C. Routson, A global Bayesian temperature calibration for lacustrine brGDGTs. *Geochim. Cosmochim. Acta* **305**, 87–105 (2021).

26. C. Turich, K. H. Freeman, Archaeal lipids record paleosalinity in hypersaline systems. *Org. Geochem.* **42**, 1147–1157 (2011).

27. H. Wang, W. Liu, Y. He, A. Zhou, H. Zhao, H. Liu, Y. Cao, J. Hu, B. Meng, J. Jiang, M. Kolpakova, S. Krivonogov, Z. Liu, Salinity-controlled isomerization of lacustrine brGDGTs impacts the associated MBT'_{5ME} terrestrial temperature index. *Geochim. Cosmochim. Acta* **305**, 33–48 (2021).

28. P. Martínez-Sosa, J. E. Tierney, L. C. Pérez-Angel, I. C. Stefanescu, J. Guo, F. Kirkels, J. Sepúlveda, F. Peterse, B. N. Shuman, A. V. Reyes, Development and application of the branched and isoprenoid GDGT machine learning classification algorithm (BIGMaC) for paleoenvironmental reconstruction. *Paleoceanogr. Paleoclimatol.* **38**, e2023PA004611 (2023).

29. J. J. Scott, L. A. Buatois, M. G. Mangano, Lacustrine environments. *Dev. Sedimentol.* **64**, 379–417 (2012).

30. M. B. Osman, J. E. Tierney, J. Zhu, R. Tardif, G. J. Hakim, J. King, C. J. Poulsen, Globally resolved surface temperatures since the Last Glacial Maximum. *Nature* **599**, 239–244 (2021).

31. T. D. Herbert, J. D. Schuffert, D. Andreasen, L. Heusser, M. Lyle, A. Mix, A. C. Ravelo, L. D. Scott, J. C. Herguera, Collapse of the California Current during glacial maxima linked to climate change on land. *Science* **293**, 71–76 (2001).

32. S. R. Hemming, Heinrich events: Massive late Pleistocene detritus layers of the North Atlantic and their global climate imprint. *Rev. Geophys.* **42**, RG1005 (2004).

33. D. McGee, E. Moreno-Chamarro, J. Marshall, E. D. Galbraith, Western U.S. lake expansions during Heinrich stadials linked to Pacific Hadley circulation. *Sci. Adv.* **4**, eaav0118 (2018).

34. J. L. Oster, S. Macarewich, M. Lofverstrom, C. de Wet, I. Montañez, J. M. Lora, C. Skinner, C. Tabor, North Atlantic meltwater during Heinrich Stadial 1 drives wetter climate with more atmospheric rivers in western North America. *Sci. Adv.* **9**, eadj2225 (2023).

35. C. Tabor, M. Lofverstrom, J. Oster, B. Wortham, C. de Wet, I. Montañez, A. Rhoades, C. Zarzycki, C. He, Z. Liu, A mechanistic understanding of oxygen isotopic changes in the Western United States at the Last Glacial Maximum. *Quat. Sci. Rev.* **274**, 107255 (2021).

36. T. Bhattacharya, J. E. Tierney, J. A. Addison, J. W. Murray, Ice-sheet modulation of deglacial North American monsoon intensification. *Nat. Geosci.* **11**, 848–852 (2018).

37. Y. Asmerom, V. J. Polyak, S. J. Burns, Variable winter moisture in the southwestern United States linked to rapid glacial climate shifts. *Nat. Geosci.* **3**, 114–117 (2010).

38. K. B. Springer, J. S. Pigati, C. R. Manker, S. A. Mahan, "The Las Vegas formation" (Professional Paper 1839, US Geological Survey, 2018); https://doi.org/10.3133/pp1839.

39. K. B. Springer, J. S. Pigati, Climatically driven displacement on the Eglington fault, Las Vegas, Nevada, USA. *Geology* **48**, 574–578 (2020).

40. J. Kim, Y. Kimura, B. Puchala, T. Yamazaki, U. Becker, W. Sun, Dissolution enables dolomite crystal growth near ambient conditions. *Science* **382**, 915–920 (2023).

41. B. D. Allen, R. Y. Anderson, A continuous, high-resolution record of late Pleistocene climate variability from the Estancia basin, New Mexico. *Geol. Soc. Am. Bull.* **112**, 1444–1458 (2000).

42. K. M. Menking, V. J. Polyak, R. Y. Anderson, Y. Asmerom, Climate history of the southwestern United States based on Estancia Basin hydrologic variability from 69 to 10 ka. *Quat. Sci. Rev.* **200**, 237–252 (2018).

43. A. M. Hudson, J. Quade, V. T. Holliday, F. Fenerty, J. E. Bright, H. J. Gray, S. A. Mahan, Paleohydrologic history of pluvial lake San Agustin, New Mexico: Tracking changing effective moisture in southwest North America through the last glacial transition. *Quat. Sci. Rev.* **310**, 108110 (2023).

44. P. L. Falkingham, K. T. Bates, M. Avanzini, M. Bennett, E. M. Bordy, B. H. Breithaupt, D. Castanera, P. Citton, I. Díaz-Martínez, J. O. Farlow, A. R. Fiorillo, S. M. Gatesy, P. Getty, K. G. Hatala, J. J. Hornung, J. A. Hyatt, H. Klein, J. N. Lallensack, A. J. Martin, D. Marty, N. A. Matthews, C. A. Meyer, J. Milàn, N. J. Minter, N. L. Razzolini, A. Romilio, S. W. Salisbury, L. Sciscio, I. Tanaka, A. L. A. Wiseman, L. D. Xing, M. Belvedere, A standard protocol for documenting modern and fossil ichnological data. *Palaeontology* **61**, 469–480 (2018).

45. M. R. Bennett, M. Budka, *Digital Technology for Forensic Footwear Analysis and Vertebrate Ichnology* (Springer Publishing, 2019); https://doi.org/10.1007/978-3-319-93689-5.

46. M. R. Bennett, D. Bustos, D. Odess, T. M. Urban, J. N. Lallensack, M. Budka, V. L. Santucci, P. Martinez, A. L. A. Wiseman, S. C. Reynolds, Walking in mud: Remarkable Pleistocene human trackways from White Sands National Park (New Mexico). *Quat. Sci. Rev.* **249**, 106610 (2020).

47. D. Webb, M. Robu, O. Moldovan, S. Constantin, B. Tomus, I. Neag, Ancient human footprints in Ciur-Izbuc Cave, Romania. *Am. J. Phys. Anthropol.* **155**, 128–135 (2014).

48. M. R. Bennett, S. C. Reynolds, "Inferences from footprints: Archaeological best practice," in *Reading Prehistoric Human Tracks: Methods & Material*, A. Pastoors, T. Lenssen-Erz, Eds. (Springer, 2021), pp. 15–39; https://doi.org/10.1007/978-3-030-60406-6.

49. M. Belvedere, M. Budka, A. L. A. Wiseman, M. R. Bennett, When is enough, enough? Questions of sampling in vertebrate ichnology. *Palaeontology* **64**, 661–672 (2021).

50. A. L. A. Wiseman, I. De Groote, One size fits all? Stature estimation from footprints and the effect of substrate and speed on footprint creation. *Anat. Rec.* **305**, 1692–1700 (2022).

51. R. G. Snyder, L. W. Schneider, C. L. Owings, H. M. Reynolds, D. H. Golomb, M. A. Schork, "Anthropometry of infants, children and youths to age 18 for product safety design" (US Government Printing Office, 1977).

52. K. G. Hatala, W. E. H. Harcourt-Smith, A. D. Gordon, B. W. Zimmer, B. G. Richmond, B. L. Pobiner, D. J. Green, A. Metallo, V. Rossi, C. M. Liutkus-Pierce, Snapshots of human anatomy, locomotion, and behavior from Late Pleistocene footprints at Engare Sero, Tanzania, Tanzania. *Sci. Rep.* **10**, 7740 (2020).

53. J. S. Vogel, J. R. Southon, D. E. Nelson, T. A. Brown, Performance of catalytically condensed carbon for use in accelerator mass spectrometry. *Nucl. Instrum. Methods Phys. Res. B: Beam Interact. Mater. At.* **5**, 289–293 (1984).

54. D. J. Donahue, T. W. Linick, A. J. T. Jull, Isotope-ratio and background corrections for accelerator mass spectrometry radiocarbon measurements. *Radiocarbon* **32**, 135–142 (1990).

55. J. S. Vogel, D. E. Nelson, J. R. Southon, ^{14}C background levels in an accelerator mass spectrometry system. *Radiocarbon* **29**, 323–333 (1987).

56. T. A. Brown, J. R. Southon, Corrections for contamination background in AMS ^{14}C measurements. *Nucl. Instrum. Methods Phys. Res. B: Beam Interact. Mater. At.* **123**, 208–213 (1997).

57. C. Bronk Ramsey, OxCal 4.4.4 (online software) (2025); https://c14.arch.ox.ac.uk/oxcal.html.

58. P. J. Reimer, W. E. N. Austin, E. Bard, A. Bayliss, P. G. Blackwell, C. B. Ramsey, M. Butzin, H. Cheng, R. L. Edwards, M. Friedrich, P. M. Grootes, T. P. Guilderson, I. Hajdas, T. J. Heaton, A. G. Hogg, K. A. Hughen, B. Kromer, S. W. Manning, R. Muscheler, J. G. Palmer, C. Pearson, J. van der Plicht, R. W. Reimer, D. A. Richards, E. M. Scott, J. R. Southon, C. S. M. Turney, L. Wacker, F. Adolphi, U. Büntgen, M. Capano, S. M. Fahrni, A. Fogtmann-Schulz, R. Friedrich, P. Köhler, S. Kudsk, F. Miyake, J. Olsen, F. Reinig, M. Sakamoto, A. Sookdeo, S. Talamo, The IntCal20 Northern Hemisphere radiocarbon age calibration curve (0-55 cal kBP). *Radiocarbon* **62**, 725–757 (2020).

59. E. C. Hopmans, S. Schouten, J. S. S. Damsté, The effect of improved chromatography on GDGT-based palaeoproxies. *Org. Geochem.* **93**, 1–6 (2016).

60. C. Huguet, E. C. Hopmans, W. Febo-Ayala, D. H. Thompson, J. S. S. Damsté, S. Schouten, An improved method to determine the absolute abundance of glycerol dibiphytanyl glycerol tetraether lipids. *Org. Geochem.* **37**, 1036–1041 (2006).

61. H. Lee, S. J. Feakins, Z. Lu, A. Schimmelmann, A. L. Sessions, J. E. Tierney, T. J. Williams, Comparison of three methods for the methylation of aliphatic and aromatic compounds. *Rapid Commun. Mass Spectrom.* **31**, 1633–1640 (2017).

62. B. S. Hansen, E. J. Cushing, Identification of pine pollen of late Quaternary age from the Chuska Mountains, New Mexico. *Geol. Soc. Am. Bull.* **84**, 1181–1200 (1973).

63. B. F. Jacobs, Identification of pine pollen from the southwestern United States. *AASP Contrib. Ser.* **16**, 155–168 (1985).

64. R. M. Forester, "Nonmarine calcareous microfossil sample preparation and data acquisition procedures" (Technical Procedure HP-78, R1, US Geological Survey, 1988).

65. A. J. Smith, L. D. Delorme, "Ostracoda," in *Ecology and Classification of North American Freshwater Invertebrates*, J. H. Thorpe, A. P. Covich, Eds. (Elsevier Inc., 2010), pp. 725–771.

66. A. J. Smith, D. J. Horne, "Ostracoda of the Nearctic," in *Ecology and General Biology: Thorp and Covich's Freshwater Invertebrates*, J. Thorp, D. C. Rogers, Eds. (Academic Press, 2016), vol. 2, chap. 16, pp. 477-513.

67. R. M. Forester, A. J. Smith, D. F. Palmer, B. B. Curry, North American Non-Marine Ostracode Database "NANODe" version 1, Kent State University, Kent, OH, USA (2005); www.kent.edu/nanode.

68. J. W. Williams, E. G. Grimm, J. Blois, D. F. Charles, E. Davis, S. J. Goring, R. Graham, A. J. Smith, M. Anderson, J. Arroyo-Cabrales, A. C. Ashworth, J. L. Betancourt, B. W. Bills, R. K. Booth, P. Buckland, B. Curry, T. Giesecke, S. Hausmann, S. T. Jackson, C. Latorre, J. Nichols, T. Purdum, R. E. Roth, M. Stryker, H. Takahara, The Neotoma Paleoecology Database: A multi-proxy, international community-curated data resource. *Quatern. Res.* **89**, 156–177 (2018).

69. M. Blaauw, J. A. Christen, Flexible paleoclimate age-depth models using an autoregressive gamma process. *Bayesian Anal.* **6**, 457–474 (2011).

70. C. De Jonge, E. C. Hopmans, C. I. Zell, J. H. Kim, S. Schouten, J. S. S. Damsté, Occurrence and abundance of 6-methyl branched glycerol dialkyl glycerol tetraethers in soils: Implications for palaeoclimate reconstruction. *Geochim. Cosmochim. Acta* **141**, 97–112 (2014).

71. M. Stute, J. F. Clark, P. Schlosser, W. S. Broecker, G. Bonani, A 30,000 yr continental paleotemperature record derived from noble gases dissolved in groundwater from the San Juan Basin, New Mexico. *Quat. Res.* **43**, 209–220 (1995).

72. C. J. Eastoe, D. L. Dettman, Isotope amount effects in hydrologic and climate reconstructions of monsoon climates: Implications of some long-term data sets for precipitation. *Chem. Geol.* **40**, 78–89 (2016).

73. S. J. Feakins, A. L. Sessions, Controls on the D/H ratios of plant leaf waxes in an arid ecosystem. *Geochim. Cosmochim. Acta* **74**, 2128–2141 (2010).
